# Emerging properties from mechanical tethering within a post-synthetically functionalised catenane scaffold†

**DOI:** 10.1039/d2sc04101d

**Published:** 2022-09-16

**Authors:** Nadia Hoyas Pérez, Peter S. Sherin, Victor Posligua, Jake L. Greenfield, Matthew J. Fuchter, Kim E. Jelfs, Marina K. Kuimova, James E. M. Lewis

**Affiliations:** Department of Chemistry, Imperial College London, Molecular Sciences Research Hub 82 Wood Lane London W12 0BZ UK james.lewis@imperial.ac.uk

## Abstract

Maintaining close spatial proximity of functional moieties within molecular systems can result in fascinating emergent properties. Whilst much work has been done on covalent tethering of functional units for myriad applications, investigations into mechanically linked systems are relatively rare. Formation of the mechanical bond is usually the final step in the synthesis of interlocked molecules, placing limits on the throughput of functionalised architectures. Herein we present the synthesis of a bis-azide [2]catenane scaffold that can be post-synthetically modified using CuAAC ‘click’ chemistry. In this manner we have been able to access functionalised catenanes from a common precursor and study the properties of electrochemically active, emissive and photodimerisable units within the mechanically interlocked system in comparison to non-interlocked analogues. Our data demonstrates that the greater (co-)conformational flexibility that can be obtained with mechanically interlocked systems compared to traditional covalent tethers paves the way for developing new functional molecules with exciting properties.

## Abstract 2

Using a post-synthetic modification strategy we have prepared a series of functionalised [2]catenanes to study the impact of mechanically-enforced proximity on functional group properties, including emission, electrochemistry and photoreactivity.

## Graphical abstract

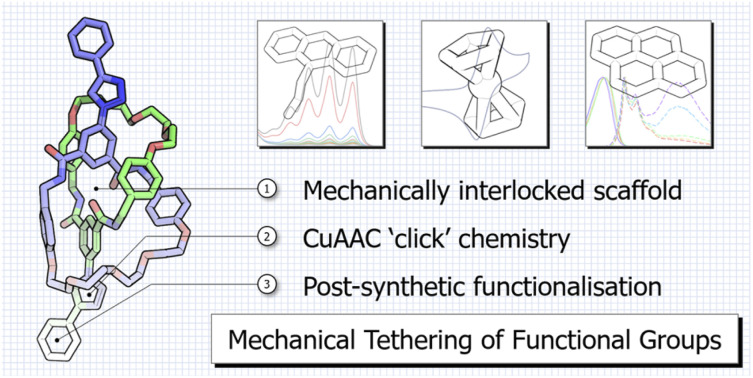

## Introduction

Mechanically interlocked molecules (MIMs)^1^ – particularly rotaxanes and catenanes – have been investigated for their utility in molecular machinery,^2^ drug delivery,^3^ catalysis,^4^ gel materials^5^ and spintronics,^6^ amongst other applications. The mechanical entanglement of components provides a route to maintain moieties in close proximity without requiring covalent modification to tether units together. Additionally, with the greater degree of (co-)conformational flexibility available to mechanically interlocked systems over covalently-linked units, there is wider scope for fine-tuning of properties and the introduction of stimuli-responsive mechanisms for modulating these. Consequently, confinement of functional moieties within interlocked molecules can result in properties that emerge not simply as an additive effect of the constituent components, but from interactions constrained by the mechanical bond. Stoddart and co-workers, for example, have recently reported [2]catenanes composed of rigid cyclophanes incorporating the organic fluorophores anthracene^7^ and pyrene.^8^ The latter displayed an increased rate of photocatalytic oxidation of 2-chloroethyl ethyl sulfide (a sulfur mustard simulant) in comparison to the non-interlocked constituent macrocycle due to enhanced ^1^O_2_ production resulting from the influence of the mechanical bond. There is a paucity of examples of such phenomena in the literature, however, that can at least be partly explained as a result of taxing synthetic routes.

A wealth of methodologies has now been elaborated to access mechanically interlocked species in high yields.^9^ These can be broadly split into two categories: passive templates (PTs), which utilise non-covalent interactions such as hydrogen-bonding,^10^ π–π interactions,^11^ anion-binding,^12^ radical-pairing^13^ or coordination bonds^14^ to pre-organise components, and active metal templates (AMTs),^15^ wherein metal ion templates catalyse covalent bond formation to trap the mechanical bond.

Mechanical bond formation is often the final step in a synthesis, with post-synthetic modification (PSM) of interlocked structures relatively underexplored.^16^ Despite this, PSM offers two key advantages: (i) functional groups incompatible with the often delicate balance of non-covalent interactions involved in templating the mechanical bond can be installed, and (ii) changes to functionality can be made without the need to synthesise new precursors and re-optimise the mechanical bond forming step.

Although there have been a handful of reports in which ‘click’ chemistry has been used to attach additional functionality to a pre-formed interlocked scaffold,^17^ there has not been, to the best of our knowledge, a systematic study to explore the extent of the utility of this approach. Such a strategy was envisaged to be of utility in probing the effects of mechanically-constrained proximity on functional groups and emergent properties arising from the mechanical bond.^18^ Herein we report the synthesis of a homocircuit [2]catenane with aryl azide head units on the constituent macrocycles. Using facile ‘click’ chemistry, a range of functional units was able to be installed following formation of the mechanical bond. For these functionalised systems, the effect of the mechanical bond on the properties of the functional moiety was investigated in comparison to their non-interlocked, macrocyclic analogues.

## Results and discussion

### Scaffold synthesis

We have previously reported the self-templated synthesis of tetraamide [2]catenanes, readily accessible from the pseudo-high dilution amide condensation^19^ between bis-benzylamines and commercially available isophthaloyl chloride.^20^ Attempts to install functional units directly onto the isophthalamide head group were unsuccessful, presumably due to increased steric bulk inhibiting appropriate pre-organisation of the components. It was hypothesised that a smaller functional handle, that could undergo subsequent reactions, might be compatible with the template motif.

**Scheme 1:**
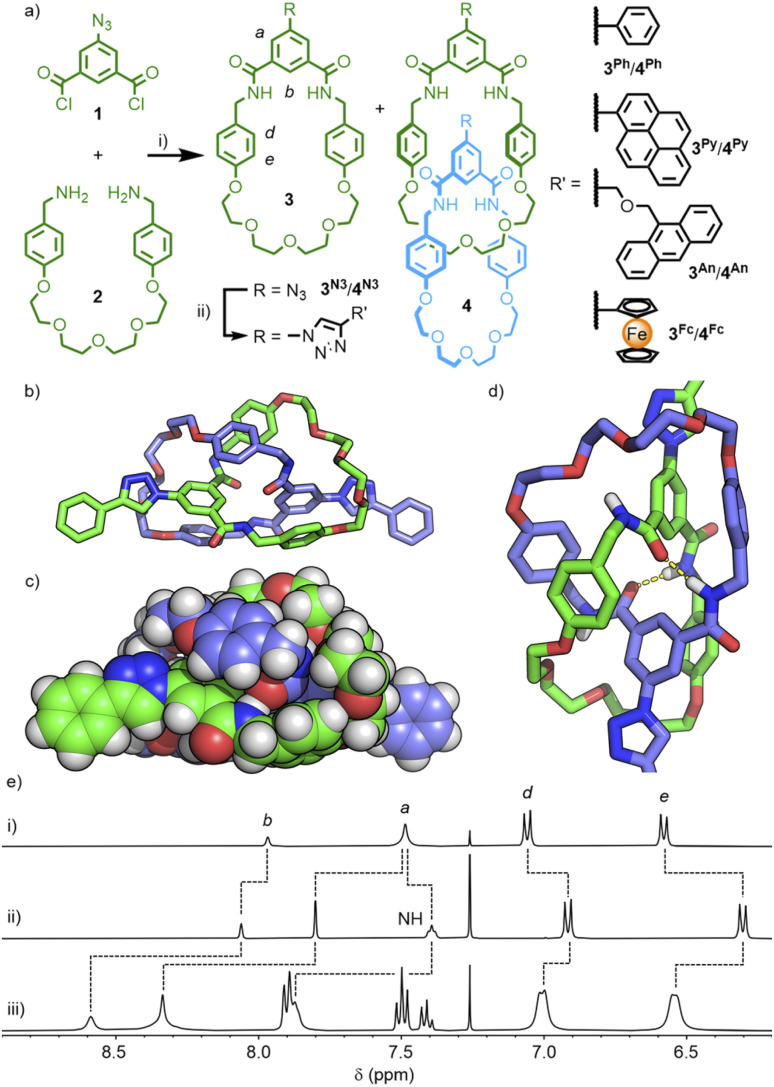
(a) Synthesis of azide-functionalised macrocycle 3^N3^ and catenane 4^N3^ and their post-synthetic CuAAC functionalisation. Reagents and conditions: (i) CHCl_3_, NEt_3_, rt, 36% (3^N3^), 34% (4^N3^); (ii) [Cu(MeCN)_4_](PF_6_), TBTA, CHCl_3_, rt, 86% (3^Ph^); 85% (4^Ph^); 41% (3^Py^); 63% (4^Py^); 71% (3^An^); 56% (4^An^); 83% (3^Fc^), 80% (4^Fc^). DFT-optimised structure (B3LYP/6-311G(d,p)) of [2]catenane 4^Ph^ shown as (b) stick model, (c) space-fill, and (d) partial structure showing inter-component hydrogen-bonds (yellow dashes). (e) Partial ^1^H NMR spectra (400 MHz, CDCl_3_, 298 K) of (i) 3^N3^, (ii) 4^N3^, and (iii) 4^Ph^.

The reaction of 5-azidoisophthaloyl chloride, 1, with bis-benzylamine 2, pleasingly, yielded a mixture of macrocycle 3^N3^ and [2]catenane 4^N3^ (Scheme 1a), that could be easily separated by column chromatography (each isolated in ∼35% yield; the interlocked identity of the catenane was confirmed by MSMS – see Fig. S15 & S16†). This allowed us to investigate the use of the copper(i)-catalysed azide–alkyne cycloaddition (CuAAC) ‘click’ reaction^21^ to introduce functional units onto the catenane scaffold.

The reaction of 4^N3^ with phenylacetylene, under standard CuAAC conditions with TBTA,^22^ resulted in conversion to the 4-phenyl-1,2,3-triazolyl substituted catenane, 4^Ph^. The simple set of NMR signals (Scheme 1e) were broadened in comparison to 4^N3^, suggesting that free pirouetting of the constituent macrocycles about each other was inhibited, to some extent, by the steric bulk of the 4-phenyl-1,2,3-triazole unit. Successful PSM of the catenane scaffold, however, had been demonstrated, with installation of a bulky group that could otherwise have prevented formation of the interlocked molecule if present in the precursor. Interestingly, the ^1^H NMR spectrum of macrocycle 3^Ph^ in CDCl_3_ was also quite broad (Fig. S22†), whilst a *d*_6_-DMSO solution gave a sharp spectrum (Fig. S17†). These observations could be explained by weak hydrogen bonding interactions between the exohedral 1,2,3-triazole unit of one macrocycle and the cavity of another (which may also explain the broad ^1^H NMR spectrum of 4^Ph^), akin to a previous report of a single amide unit being used to template rotaxane formation with a related isophthalamide macrocycle.^23^ Based on this initial success, three further macrocycle/catenane pairs were synthesised, incorporating pyrene (3^Py^/4^Py^), anthracene (3^An^/4^An^), and ferrocene (3^Fc^/4^Fc^) units. All compounds were characterised by NMR and mass spectrometry (MS); despite multiple attempts, all efforts to grow X-ray quality crystals were unsuccessful. The macrocycle/catenane pairs were then investigated to determine the effect of the mechanical-enforced proximity on the photophysical, photodimerisation and electrochemical properties, respectively, of these functional units.

Covalent tethering of functional groups is often used to augment their physical or chemical properties. For example, there has been much interest in studying electronic interactions between bridged organic units^24^ and metal centres^25^ and multi-metallocene systems,^26^ and multi-topic ligands can be used for assembling both discrete^27^ and polymeric coordination frameworks.^28^ The way these functional groups will be able to interact and operate together will largely depend upon the manner in which they are linked. Traditional covalent tethers hold moieties within close spatial proximity and can be designed to allow cooperation/communication. The use of mechanical tethering between functional groups, however, is relatively underexplored, despite the potential for introducing novel behaviour resulting from the dynamic nature of the mechanical bond.

### Photophysical properties of pyrene-functionalised systems

Fluorescent macrocycles, including those incorporating pyrene moieties, are attractive systems for various applications resulting from a combination of their host–guest chemistry and photophysical properties.^29^ Pyrene fluorescence is characterised by monomer emission at approximately 380–400 nm and structureless excimer emission at ∼480–500 nm, arising from an excimer formed from close spatial proximity of two pyrene moieties, one in an excited state and the second in its ground state.^30^ We were motivated to investigate whether mechanical entanglement of two pyrene-functionalised macrocycles would lead to properties distinct from the non-interlocked component.

**Fig. 1:**
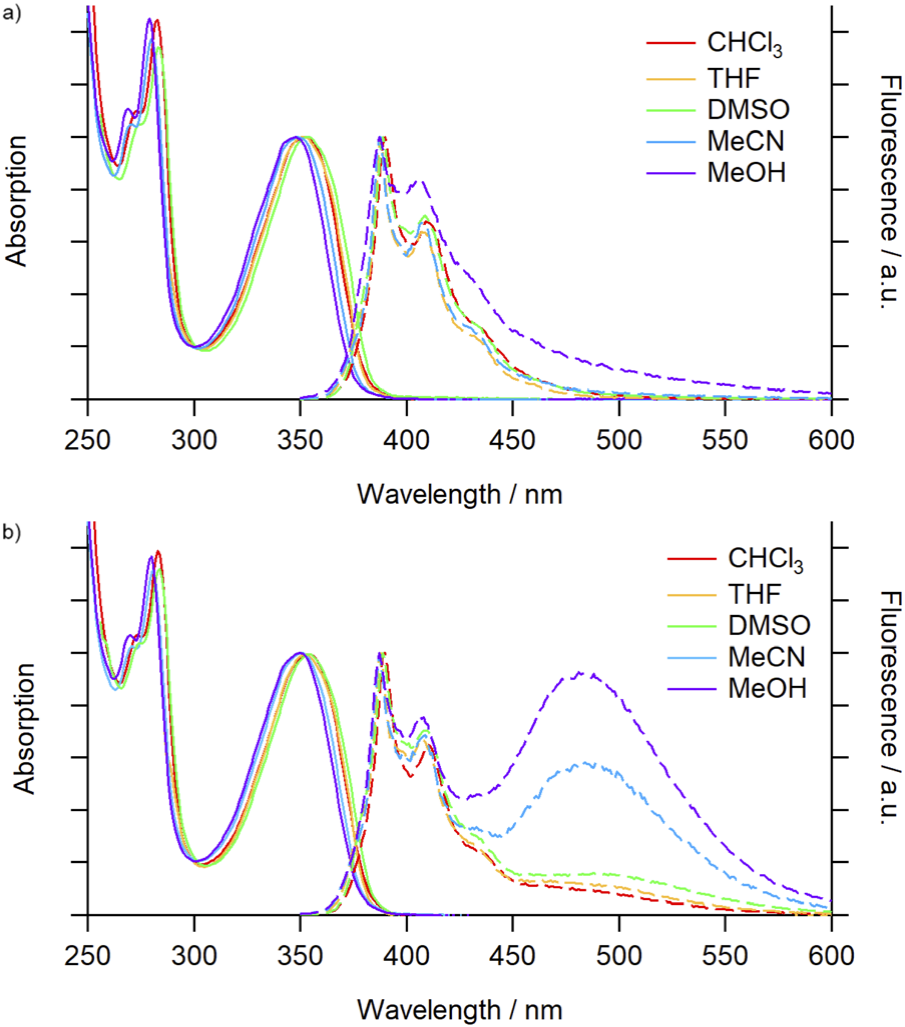
Absorption (solid trace) and emission (dashed trace) spectra (air-equilibrated) of (a) 3^Py^ (5.2 × 10^−6^ M) and (b) 4^Py^ (2.6 × 10^−6^ M) in various solvents.

Initially a comparison of the electronic absorption and emission spectra of 3^Py^ and 4^Py^ in a variety of solvents was undertaken to observe any solvatochromic effects. The absorption spectra of the macrocycle and catenane exhibited minimal shifts across the solvents investigated (Fig. 1). No significant differences between the two species were observed, suggesting minimal impact of the mechanical bond on the ground state properties of the pyrene chromophore.

For 3^Py^ the steady-state emission spectra (*λ*_ex_ = 340 nm, Fig. 1a) were again minimally perturbed upon changing solvent and were dominated by characteristic pyrene monomer emission peaks (*λ*_em_ = 387–440 nm). Increasing the concentration (3–25 μM) of the solution also had no noticeable impact on the emission (Fig. S71†). For the [2]catenane, 4^Py^ (Fig. 1b), in low polarity, aprotic solvents (toluene, CHCl_3_ and THF) monomer emission (*λ*_em_ = 388 nm) was accompanied by a weak, broad band centred at ∼490 nm, assigned to excimer formation. In the more polar DMSO and DMF solvents a slight enhancement of this emission was seen. In MeCN and MeOH, however, excimer emission was significant, reaching intensities commensurate with the monomer (Table 1). The ratio of the excimer and monomer emission intensities (*I*_E_/*I*_M_) was highly sensitive to the presence of polar protic solvents in binary mixtures with MeCN, and could be tuned from 0.6 (100% MeCN) to 1.6 (20% MeOH; Fig. S73†) and 2.2 (29% H_2_O; Fig. S70 and S73†). The emission profile in MeCN did not vary significantly over two orders of magnitude change in concentration (1–100 μM, Fig. S74†), suggesting that the excimer emission arose essentially exclusively from unimolecular excimer formation between the two mechanically interlocked pyrene moieties. Thus, through mechanical bonding, we have been able to decouple the process of excimer formation from the bulk concentration of pyrene units. Additionally, excimer emission displays no clear correlation with solvent polarity or viscosity, suggesting a complex interplay between the properties of the solvents and their interactions with both the pyrene unit and the 4^Py^ scaffold that affects the emission profile.

**Table 1:** Emission data for catenane 4^Py^ (2.6 × 10^−6^ M) in various solvents, including ratio of excimer to monomer emission intensities (*I*_E_/*I*_M_), fluorescence quantum yield (*Φ*_F_), and lifetimes for the two components of excimer emission (*τ*_e1_ and *τ*_e2_)

Solvent	*I*_E_/*I*_M_	*Φ*_F_	*τ*_e1_ (ns)	*τ*_e2_ (ns)
Toluene	—a	0.427	15.0	20.7
CHCl_3_	—a	0.387 (0.615)^b^	16.0	27.0
THF	0.110	0.294 (0.871)^b^	14.3	28.6
DMF	0.144	0.437	17.7	32.7
DMSO	0.135	0.646	23.8	62.1
MeCN	0.627	0.212 (0.526)^b^	12.3 (27.3)^b^	14.2 (62.5)^b^
MeOH	0.963	0.039 (0.056)^b^	8.6	22.1

a Excimer emission too weak to determine.

b N_2_-purged solution.

As a concentration-independent parameter, fluorescence lifetime offers significant advantages for applications such as sensing, compared to fluorescence intensity. The time-resolved fluorescence decay of the macrocycle monomer emission could be fitted by a monoexponential decay function. The catenane excimer emission, however, was best fitted by a triexponential decay: one component for the quenching of monomer emission (Table S1,†*τ*_m_) and two components resulting from the formation of long-lived excimers (Table 1, *τ*_e1_ and *τ*_e2_). Two component time resolved decay is a hallmark of excimer formation from free pyrene;^31^ the appearance of the three-component decay must be a consequence of the mechanical bond restricting the (co-)conformational flexibility of the current molecule, leading to coexistence of two different excimer (co-)conformations. The emission lifetimes were commensurate with previously reported covalently-linked pyrene dimers, and varied slightly with solvent, reaching a maximum in DMSO (*τ*_e1_ = 23.8 ns, *τ*_e2_ = 62.1 ns).

**Fig. 2:**
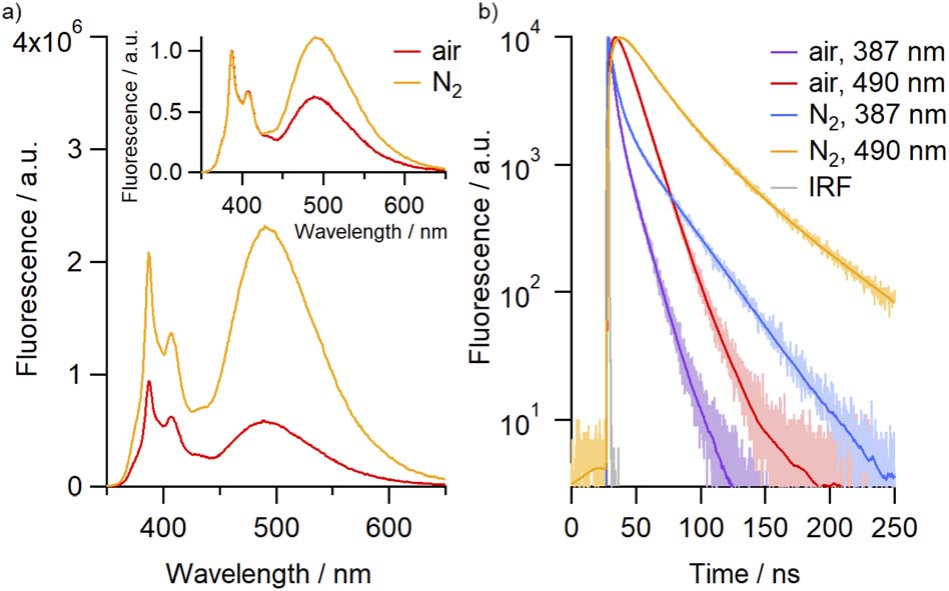
(a) Emission spectra of 4^Py^ in air-equilibrated (red) and N_2_-saturated (orange) MeCN (2.6 × 10^−6^ M). Inset: the same spectra after normalization. (b) Fluorescence time profiles recorded with 4^Py^ in air-equilibrated and N_2_-saturated MeCN at emission wavelengths corresponding to the emission from monomer (387 nm) and excimer (490 nm), including IRF (instrument response function). Smooth lines are the best fits of three-exponential global analysis.

Molecular oxygen is known to act as an efficient quencher of pyrene fluorescence,^32^ prompting us to investigate the effect of O_2_ on the emission profile of the catenane. The intensity of both monomer and excimer emissions were observed to increase following purging of the solutions with N_2_, with the effect most pronounced for the excimer band at 490 nm (Fig. 2). Indeed, the quantum yield for the excimer emission consistently increased, in some cases by up to almost three-fold, between air-saturated and N_2_-purged samples across the solvents investigated, alongside significant enhancements in the emission lifetime (Table 1, Fig. S75†). This data suggests that these dyes can serve as oxygen sensors, both by fluorescence lifetime and by ratiometric detection, with both measurements being independent of the catenane concentration.

**Fig. 3:**
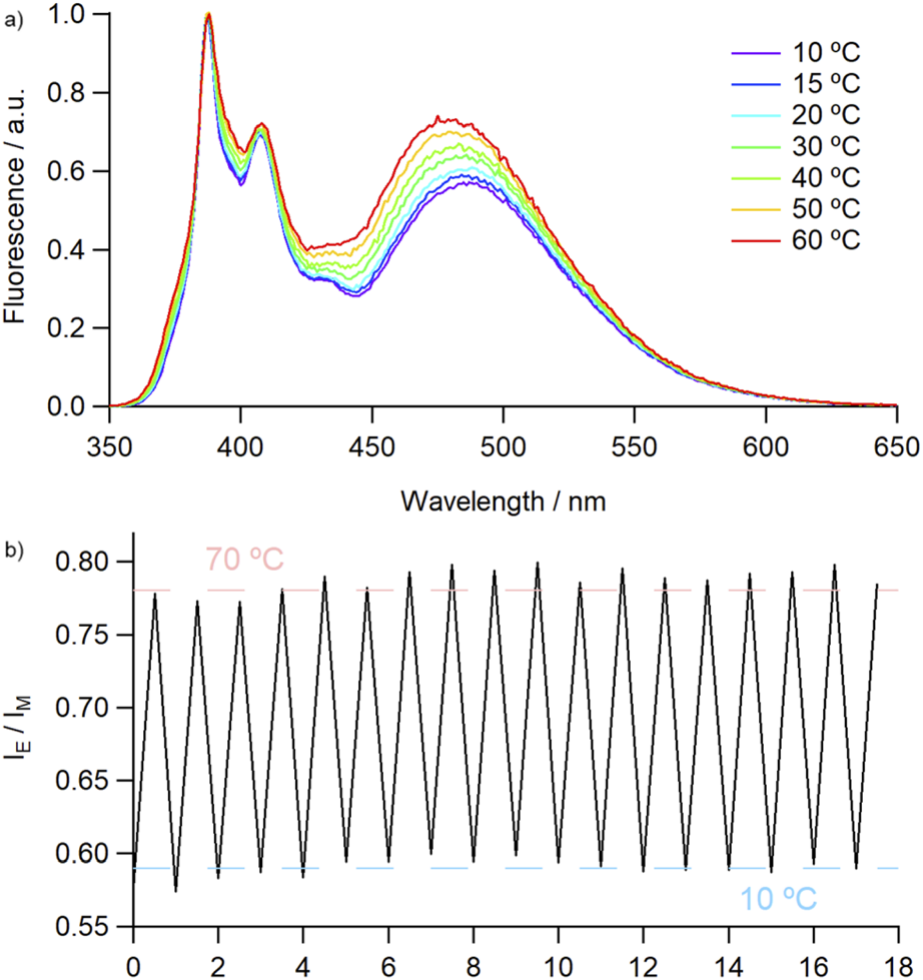
(a) Temperature-dependent emission of 4^Py^ in MeCN (2.6 × 10^−6^ M), normalised to monomer emission peak and (b) *I*_E_/*I*_M_ ratio during thermal cycling (10 ⇌ 70 °C).

Covalently-linked pyrene dimers have been previously shown to exhibit thermoresponsive behaviour, with an increase in *I*_E_/*I*_M_ values resulting from a combination of thermal quenching of monomer emission and increased facilitation of excimer formation due to decreased bulk viscosity.^33^ This led us to investigate the effect of temperature on the emission profile of 4^Py^ in various solvents. In each instance, although the absolute intensities of both monomer and excimer emission were found to be inversely proportional to temperature, they exhibited different rates of change (Fig. S76 and S77†). This allowed us to employ the *I*_E_/*I*_M_ value as a ratiometric measure of temperature, with an approximately linear increase in this value observed over the temperature range measured (10 to 60–80 °C). The robustness of the catenane framework was examined over multiple thermal cycles in MeCN, with no deterioration in the fluorescence output (Fig. 3).

Without any covalent modification, we have demonstrated the ability to dramatically alter the photophysical properties of a pyrene-functionalised macrocycle through mechanical entanglement. The enforced proximity of pyrene units brought about through the mechanical bond promoted excimer formation which, whilst unaffected by concentration, was found to be sensitive to a range of environmental changes. Although MIMs have been used as sensors for cations and anions,^34^ their use as sensors of neutral species or environmental conditions has been very limited. In this work, 4^Py^ displayed utility as a molecular thermometer, oxygen sensor and probe of bulk medium composition. The greater (co-)conformational freedom available to mechanically interlocked arrays in comparison to traditional covalent systems opens the door for fine-tuning sophisticated functional systems.

### Electrochemical properties of ferrocene-functionalised systems

Ferrocene – the archetypal metallocene – is an organometallic moiety of interest for its use in catalysis,^35^ drug delivery,^36^ materials,^37^ molecular electronics,^38^ and as a “ball-bearing” in molecular machines.^39^ It has previously been incorporated into MIMs for the electrochemical sensing of charged species^40^ and as a component for switchable electron-transfer processes.^41^ To the best of our knowledge, however, there has yet to be an investigation into the potential for electronic communication between ferrocenyl units across a mechanical bond.^42^

CuAAC reaction of 3^N3^ and 4^N3^ with ethynylferrocene successfully generated the desired functionalised macrocycle and catenane (3^Fc^ and 4^Fc^, respectively). Previous reports have demonstrated solvent dependent electronic communication between ferrocene units across a hydrogen bonded dimer,^43^ and the ability to use this to electrochemically control dimer assembly/disassembly.^44^ As such, ferrocene units appended to the macrocycles of 4^Fc^ might allow reversible, electrochemical control over co-conformation of the catenane.^45^

Cyclic voltammetry (CV) of the ferrocene-appended scaffolds, 3^Fc^ and 4^Fc^, in MeCN each showed single, completely reversible oxidation processes (Fig. S78†) at similar potentials (Table 2). As MeCN could disrupt hydrogen bonding between the interlocked macrocycles in 4^Fc^, we subsequently investigated the electrochemical properties in apolar CH_2_Cl_2_, as a change in co-conformation could bring the two ferrocenyl moieties into closer proximity. As in MeCN, CV of both species in CH_2_Cl_2_ demonstrated single, reversible oxidation processes (Fig. S79†) with minimal differences in the potentials for the macrocycle and catenane.

**Table 2:** Electrochemical data for macrocycle 3^Fc^ and catenane 4^Fc^ (10^−4^ M). TBAPF_6_ electrolyte at 0.1 M under argon atmosphere. Potentials are references against Fc/Fc^+^

	*E*/V	
Solvent	3^Fc^	4^Fc^
MeCN	0.057	0.023
CH_2_Cl_2_	0.077	0.041

**Fig. 4:**
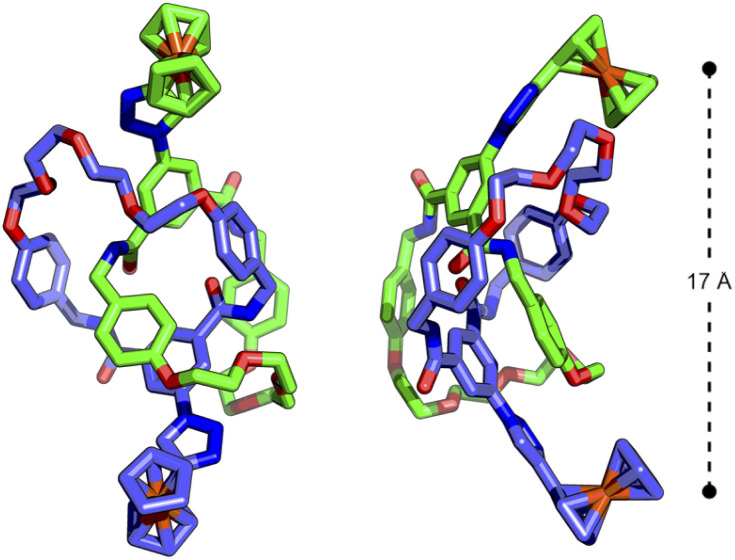
DFT-optimised structure (B3LYP/6-311G(d,p)) of [2]catenane 4^Fc^ showing distance between interlocked ferrocenyl units.

The similarity between the processes in 3^Fc^ and 4^Fc^ disappointingly suggested a lack of communication between the ferrocene units in the catenane. Presumably, despite being tethered by the mechanical bond, the ferrocene units are not held in an appropriate manner to effect any significant electrostatic interactions. Indeed, the DFT-optimised structure (Fig. S90 and S91†) of 4^Fc^ showed the Fe ions to be located ∼17 Å apart (Fig. 4).

The isophthalamide motif has been used extensively for binding anions in acyclic,^46^ cyclic^47^ and mechanically interlocked receptors.^48^ Although we were not able to observe electronic communication between the ferrocene units in 4^Fc^, successful installation of electroactive moieties onto the catenane scaffold could potentially enable these systems to be used for anion sensing.^49^ Efforts towards investigating this are ongoing within our lab, although outside the scope of this report.

### Photodimerisation with anthracene-functionalised systems

Turning to macrocycle 3^An^ and catenane 4^An^, photoirradiation of anthracenes with an appropriate wavelength serves to effect a [4π + 4π] photocycloaddition, with reversion to the monomer usually achieved under thermal or photochemical conditions.^50^ This reaction has been used previously in MIMs for topological interconversion^51^ and reversible polymerisation.^52^

**Fig. 5:**
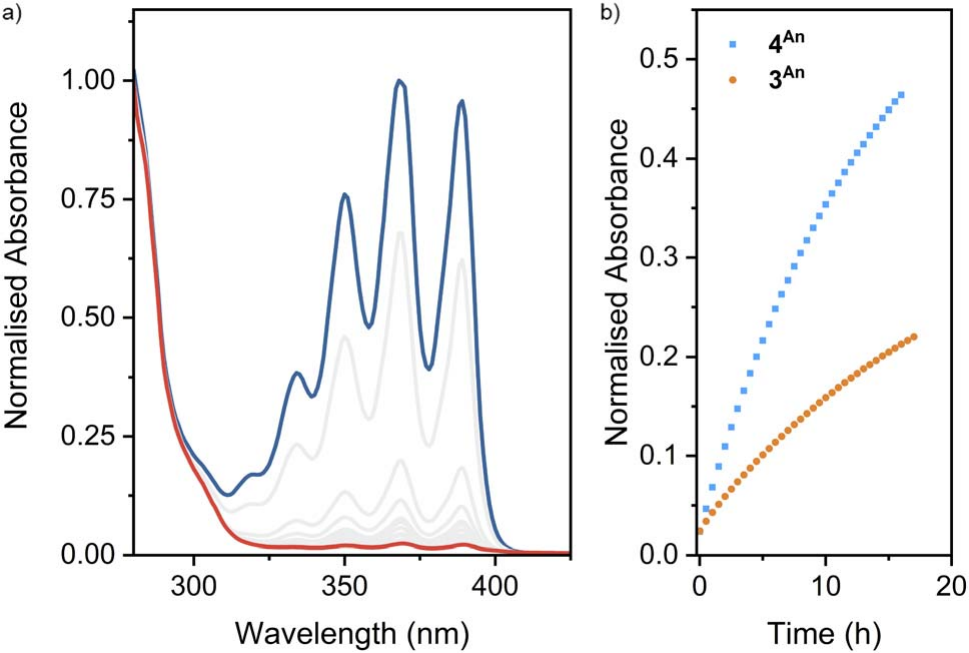
(a) Absorption spectra of 4^An^ (2.5 × 10^−4^ M) in DMSO before (blue) and after (red) 15 min irradiation at 365 nm and (b) thermal cycloreversion (*λ*_abs_ = 389 nm) of 3^An^ (5 × 10^−4^ M) and 4^An^ (2.5 × 10^−4^ M) at room temperature.

**Fig. 6:**
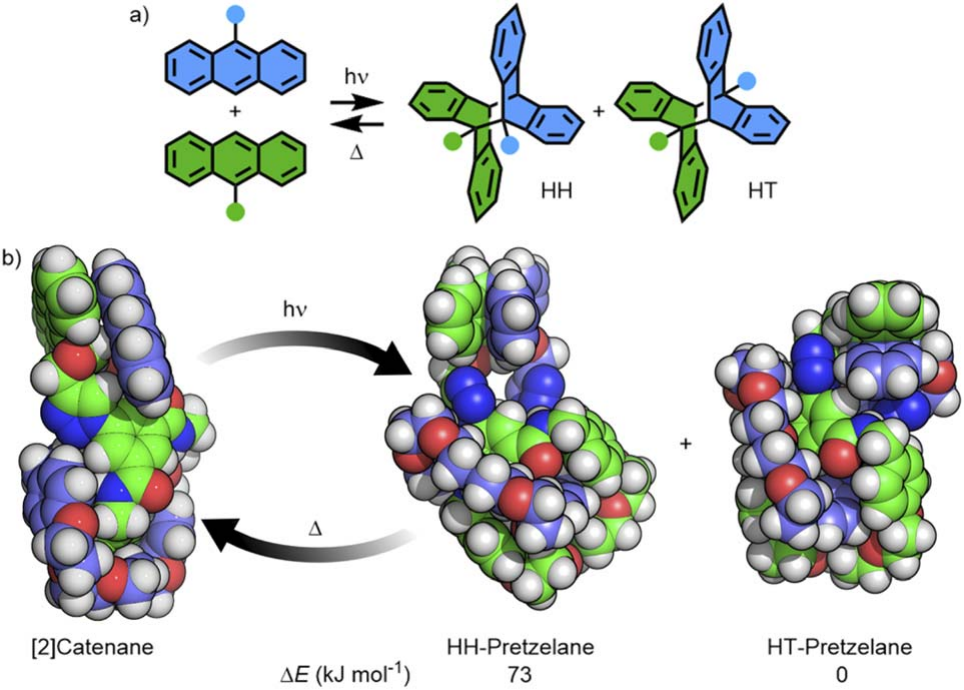
(a) Reversible photoisomerisation of 9-substituted anthracenes giving HH and HT dimer isomers; (b) [2]catenane 4^An^ forming a mixture of the HH- and HT-pretzelanes, the former of which underwent thermal cycloreversion at room temperature.

The photoreactivities of macrocycle 3^An^ and catenane 4^An^ were examined by UV-vis spectroscopy. Irradiation of dilute (5 × 10^−4^ M with respect to the anthracene moiety) solutions in degassed DMSO with 365 nm light resulted in loss of the long-wavelength anthracene absorption band (∼325–400 nm), indicative of successful photodimerisation (Fig. 5). As might be expected, photodimerisation of the catenated system appeared to occur more rapidly (<4 min and >10 min for >95% conversion for the catenane and macrocycle, respectively; Table S3†), likely a result of the increased relative concentration of anthracene units within the mechanically interlocked system. This was supported by the marked increased in quantum yield of dimerisation for the catenane compared to the macrocycle – 71% *vs.* 15% (Fig. S85 and S86†). On the assumption that at this low concentration in solution the photodimerisation of 4^An^ occurred exclusively in an intramolecular fashion, irradiation serves to effect conversion between catenane and pretzelane^53^ topologies (Fig. 6b).

Thermal cycloreversion was observed for both compounds at room temperature (Fig. 5, S82 and S83†), with the anthracene absorption band returning after 16 h to approximately 49% and 23% of its original intensity for the catenane and macrocycle, respectively. Interestingly, the catenane displayed a markedly higher rate of reversion (1.1 × 10^−5^ s^−1^) compared to the macrocycle (3.9 × 10^−6^ s^−1^).

Photodimerisation of 9-substituted anthracenes can proceed to give head-to-head (HH) and head-to-tail (HT) isomers (Fig. 6a). Traditionally the HT isomer is more thermally stable, with cycloreversion of HH isomers previously reported to occur at room temperature.^54^ Indeed, from the calculated structures of the HH and HT photodimers of 3^An^, the former was found to be higher in energy by approximately 18 kJ mol^−1^ (Table S5†).

The ground-state structure of 4^An^ was calculated using DFT methods (Fig. S94†) and the anthracene substituents observed to form π-stacking interactions in a head-to-head arrangement (Fig. 6b). Furthermore, the calculated structures of the HH- and HT-pretzelane photoadducts suggested the former was significantly (73 kJ mol^−1^) higher in energy than the latter – consistent with previous calculations on non-interlocked systems.^55^ The combined experimental and calculated data suggest that (co-)conformational restrictions imposed by the mechanical bond enhanced formation of the less thermally stable HH cycloadduct (HH-pretzelane) in comparison to the non-interlocked macrocycle, which would explain the greater degree and rate of cycloreversion for the interlocked system. Disappointingly, attempts to probe the formation of different cycloadduct isomers by ^1^H NMR were hampered by complex spectra and poor signal-to-noise ratios at the concentrations investigated (Fig. S87†).

## Conclusions

In summary, using a post-synthetic modification strategy, we have prepared a range of functionalised homocircuit [2]catenane systems. These include interlocked systems incorporating pyrene, anthracene and ferrocene substituents, for which we investigated the effect of the mechanical bond on the photophysical, photoswitchable and electrochemical properties, respectively. With myriad synthetic techniques making mechanically interlocked scaffolds readily accessible, exploration of their applications is expanding. Herein we have demonstrated the utility of a post-synthetic modification strategy to derive functional interlocked molecules from a common precursor. This obviates the need for synthesising a raft of functionalised precursors and allows access to systems for which bulky starting materials would have inhibited formation of the mechanical bond. Through a comparison of the properties of interlocked systems and their non-interlocked analogues we have also been able to glean some fundamental understanding of the impact of mechanical bonding on chemical and physical properties that are not simply an additive effect of the constituent macrocycles. The ability to design systems with emergent behaviours arising from mechanical tethering will allow access to new, sophisticated functional materials for wide-ranging applications.

## Data availability

Data are available upon request from the authors.

## Author contributions

NHP – Synthesis & characterisation; PSS – spectroscopy; VP – computational simulations; JLG – electrochemistry & spectroscopy; MJF, KEJ and MKK – supervision; JEML – conceptualisation, molecule design, supervision, analysis & writing – original draft. All authors contributed to data analysis & editing and reviewing of the manuscript.

## Conflicts of interest

There are no conflicts to declare.

## Supplementary Material

SC-013-D2SC04101D-s001

SC-013-D2SC04101D-s002

SC-013-D2SC04101D-s003

SC-013-D2SC04101D-s004

SC-013-D2SC04101D-s005

SC-013-D2SC04101D-s006

SC-013-D2SC04101D-s007

SC-013-D2SC04101D-s008
