# Supplementary material for: Emerging properties from mechanical tethering within a post-synthetically functionalised catenane scaffold
Source: Chem Sci. 2022 Sep 16;13(38):11368–75. doi: 10.1039/d2sc04101d (PMC9533469; doi:10.1039/d2sc04101d)
Supplement: SC-013-D2SC04101D-s001 [file SC-013-D2SC04101D-s001.pdf]

Electronic Supplementary Material (ESI) for Chemical Science.
This journal is © The Royal Society of Chemistry 2022

Electronic Supporting Information

Emerging properties from mechanical tethering within a post-synthetically functionalised catenane scaffold

Nadia Hoyas Pérez,[a] Peter S. Sherin,[a] Victor Posligua,[a] Jake L. Greenfield,[a,b] Matthew J. Fuchter,[a] Kim E. Jelfs,[a] Marina K. Kuimova[a] and James E. M. Lewis[a,c]*

[a]Department of Chemistry, Imperial College London, Molecular Sciences Research Hub,

82 Wood Lane, London W12 0BZ, UK

[b]Center for Nanosystems Chemistry (CNC), Universität Würzburg, Würzburg 97074, Germany;

Institut für Organische Chemie, Universität Würzburg, Würzburg 97074, Germany

[c]School of Chemistry, University of Birmingham, Edgbaston, Birmingham B15 2TT, UK

*j.e.m.lewis@bham.ac.uk

Contents

1. General Experimental 3
2. Synthetic Procedures 4
  Synthesis of **S2** 4
  Synthesis of **1** 5
  Synthesis of $\mathbf{3^{N3}}$ and $\mathbf{4^{N3}}$ 6
  General CuAAC Procedure 14
  Synthesis of $\mathbf{3^{Ph}}$ 14
  Synthesis of $\mathbf{4^{Ph}}$ 18
  Synthesis of $\mathbf{3^{Py}}$ 22
  Synthesis of $\mathbf{4^{Py}}$ 26
  Synthesis of $\mathbf{3^{An}}$ 30
  Synthesis of $\mathbf{4^{An}}$ 34
  Synthesis of $\mathbf{3^{Fc}}$ 37
  Synthesis of $\mathbf{4^{Fc}}$ 41
3. Spectroscopic and Photophysical Data of $\mathbf{3^{Py}}$ & $\mathbf{4^{Py}}$ 45
  Experimental procedures 45
  Spectroscopic and Photophysical Data 46
4. Electrochemical data for $\mathbf{3^{Fc}}$ and $\mathbf{4^{Fc}}$ 55
5. Photoirradiation data for $\mathbf{3^{An}}$ and $\mathbf{4^{An}}$ 56
6. Computational studies of catenanes 62
7. References 66

1. General Experimental

Synthesis: Unless otherwise stated, all reagents, including anhydrous solvents, were purchased from commercial sources and used without further purification. $CDCl_3$ and NEt_3 were stored over 4 Å molecular sieves prior to use. All reactions were carried out under an atmosphere of N_2 using degassed, anhydrous solvents unless otherwise stated. Petrol refers to the fraction of petroleum ether boiling in the range 40-60 °C. Analytical TLC was performed on pre-coated silica gel plates (0.25 mm thick, 60F254, Merck, Germany) and observed under UV light. EDTA solution refers to a 0.1 M solution of EDTA-Na_2 in 3% $NH_{3(aq)}$.

Analysis: NMR spectra were recorded on Bruker AV400 or AV500 instrument, at a constant temperature of 300 K. Chemical shifts are reported in parts per million from low to high field and referenced to residual solvent. Standard abbreviations indicating multiplicity were used as follows: m = multiplet, quint = quintet, q = quartet, t = triplet, d = doublet, s = singlet, app. = apparent, br. = broad. Signal assignment was carried out using 2D NMR methods (HSQC, HMBC, COSY, NOESY) where necessary. In the case of some signals absolute assignment was not possible. Here indicative either/or assignments (e.g. H_A/H_B for H_A or H_B) are provided. All melting points were determined using a hot stage apparatus and are uncorrected. Mass spectrometry was carried out by the Imperial College London, Department of Chemistry Mass Spectroscopy Service using Waters LCT Premier for HR-ESI-MS and Thermo Scientific Q-Exactive for tandem MS.

S1 was synthesised according to a literature procedure.[1]

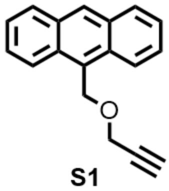

S1

2. Synthetic Procedures

Synthesis of S2

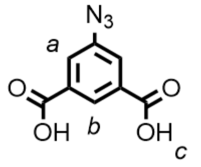

To 5-aminobenzene-1,3-dicarboxylic acid (0.905 g, 5 mmol, 1 eq.) in 1M HCl (40 mL) at 0 °C was added a solution of $NaNO_2$ (0.517 g, 7.5 mmol, 1.5 eq.) in the smallest amount of H_2O. After 30 minutes a solution of NaN_3 (0.65 g, 10 mmol, 2 eq.) in the smallest amount of water was added dropwise. The reaction mixture was stirred, allowing to warm to rt, overnight. The resultant precipitate was filtered on paper and washed with water, giving **S2** as a pale brown solid (0.85 g, 82%) that was used without further purification. ^{1}H NMR (400 MHz, d_6-DMSO) δ: 13.56 (br. s, 2H, H_c), 8.24 (s, 1H, H_b), 7.77 (s, 2H, H_a). Spectroscopic data were consistent with literature data.[2]

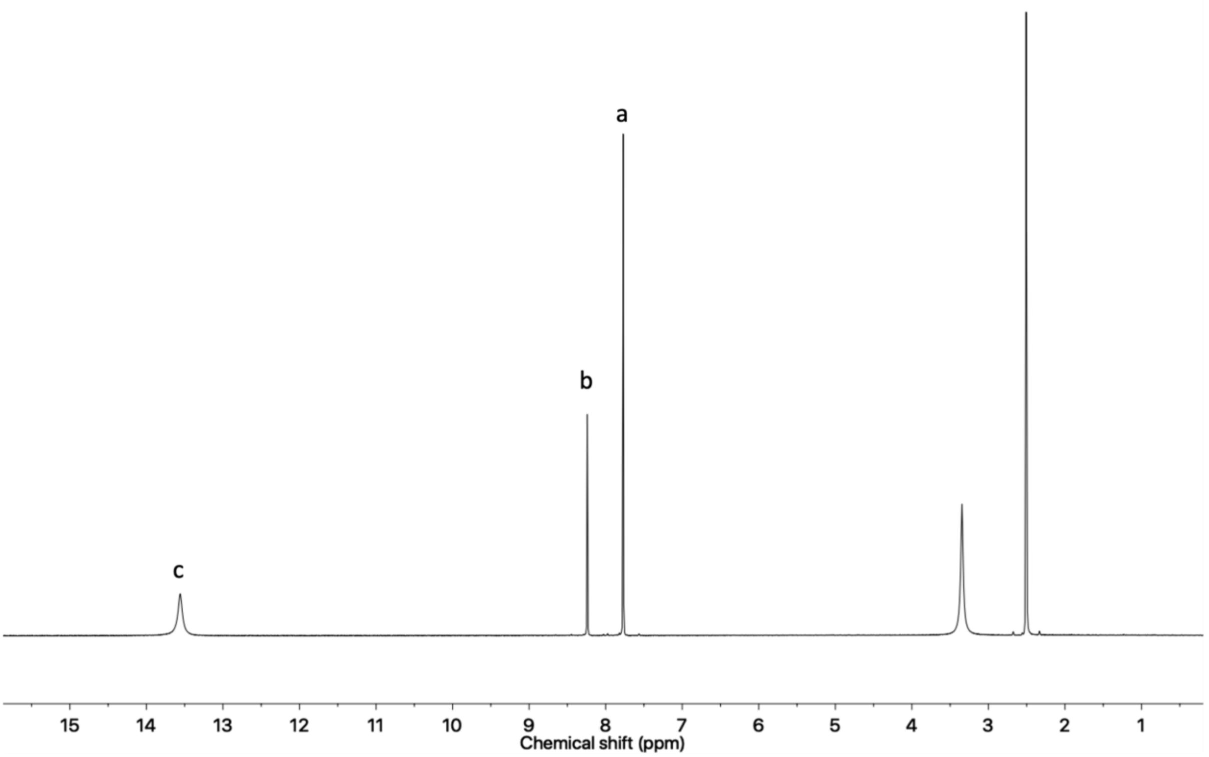

Figure S1 ^{1}H NMR (d_6-DMSO, 400 MHz) of **S2**.

Synthesis of 1

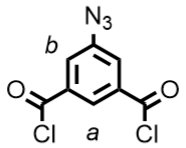

To a suspension of **S2** in CH_2Cl_2 (12 mL) was added oxalyl chloride (0.12 mL, 1.5 mmol, 3 eq.) followed by DMF (2 drops) and the reaction mixture stirred at rt for 40 minutes. The solvent was removed *in vacuo* giving **1** as a yellow solid that was used without further purification. ^{1}H NMR (400 MHz, d_6-DMSO) δ: 8.55 (s, 1H, H_a), 7.65 (s, 2H, H_b).

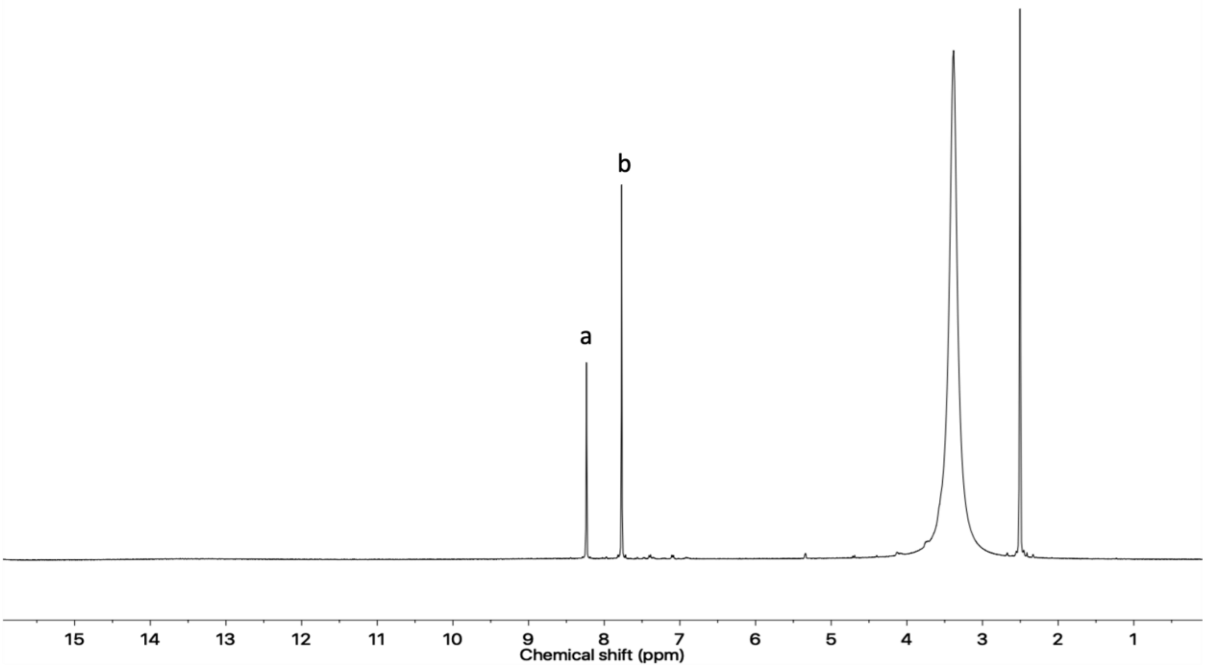

Figure S2 ^{1}H NMR (d_6-DMSO, 400 MHz) of **1**.

Synthesis of 3^{N3} and 4^{N3}

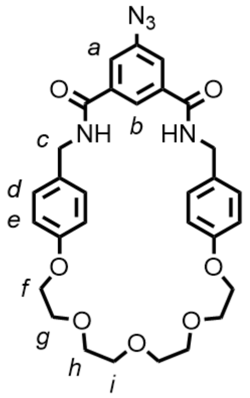

A solution of **1** (0.122 g, 0.5 mmol, 1 eq.) in $CHCl_3$ (40 mL) and a solution of **2** (0.202 g, 0.5 mmol, 1 eq.) in $CHCl_3$ (40 mL) were added simultaneously over 2 h via syringe pump into a solution of NEt_3 (0.17 mL, 1.25 mmol, 2.5 eq.) in $CHCl_3$ (40 mL) and the reaction mixture subsequently stirred overnight. The solvent was removed *in vacuo* and the products purified by column chromatography on silica to give $\mathbf{3^{N3}}$ (1:4 acetone/CH_2Cl_2) as a yellow solid (103.7 mg, 36%) and $\mathbf{4^{N3}}$ (2:3 acetone/CH_2Cl_2) as a pale brown solid (97.8 mg, 34%).

$\mathbf{3^{N3}}$

^{1}H NMR (400 MHz, $CDCl_3$) δ: 7.97 (s, 1H, H_b), 7.49 (app. s, 4H, H_a, H_{NH}), 7.06 (d, J = 8.6 Hz, 4H, H_d), 6.58 (d, J = 8.6 Hz, 4H, H_e), 4.38 (d, J = 4.9 Hz, 4H, H_c), 3.79-3.72 (m, 16H, H_f, H_g, H_h, H_i).

^{13}C NMR (101 MHz, $CDCl_3$) δ: 165.7, 158.0, 141.1, 134.7, 129.7, 129.5, 122.1, 120.6, 114.6, 70.9 (×2), 69.8, 67.2, 44.3.

HR-ESI-MS *m/z* = 598.2283 $[M+Na]^+$ calc. 598.2297.

M.p. 166±1 °C.

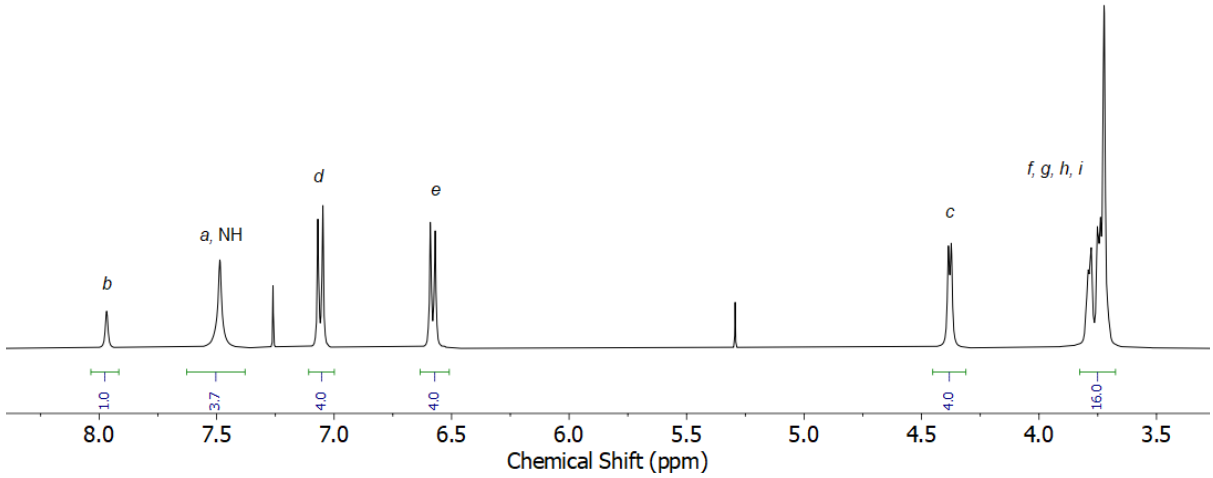

Figure S3 ^{1}H NMR ($CDCl_3$, 400 MHz) of $\mathbf{3^{N3}}$

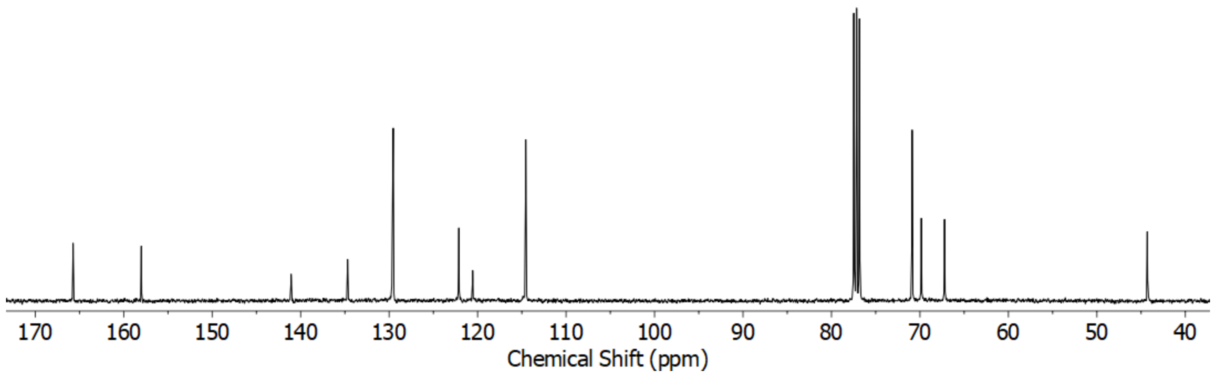

Figure S4 ^{13}C NMR ($CDCl_3$, 101 MHz) of **3**$^{\mathbf{N3}}$

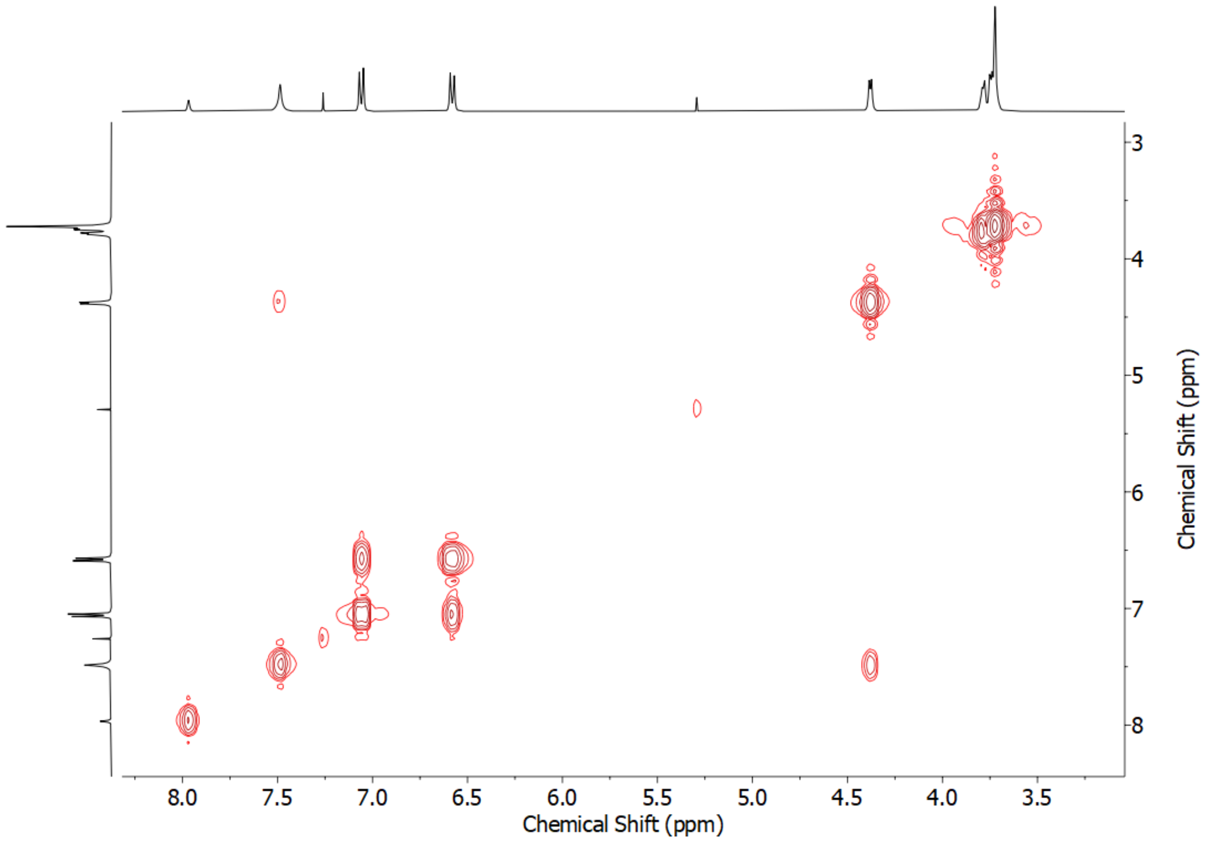

Figure S5 COSY NMR ($CDCl_3$) of **3**$^{\mathbf{N3}}$

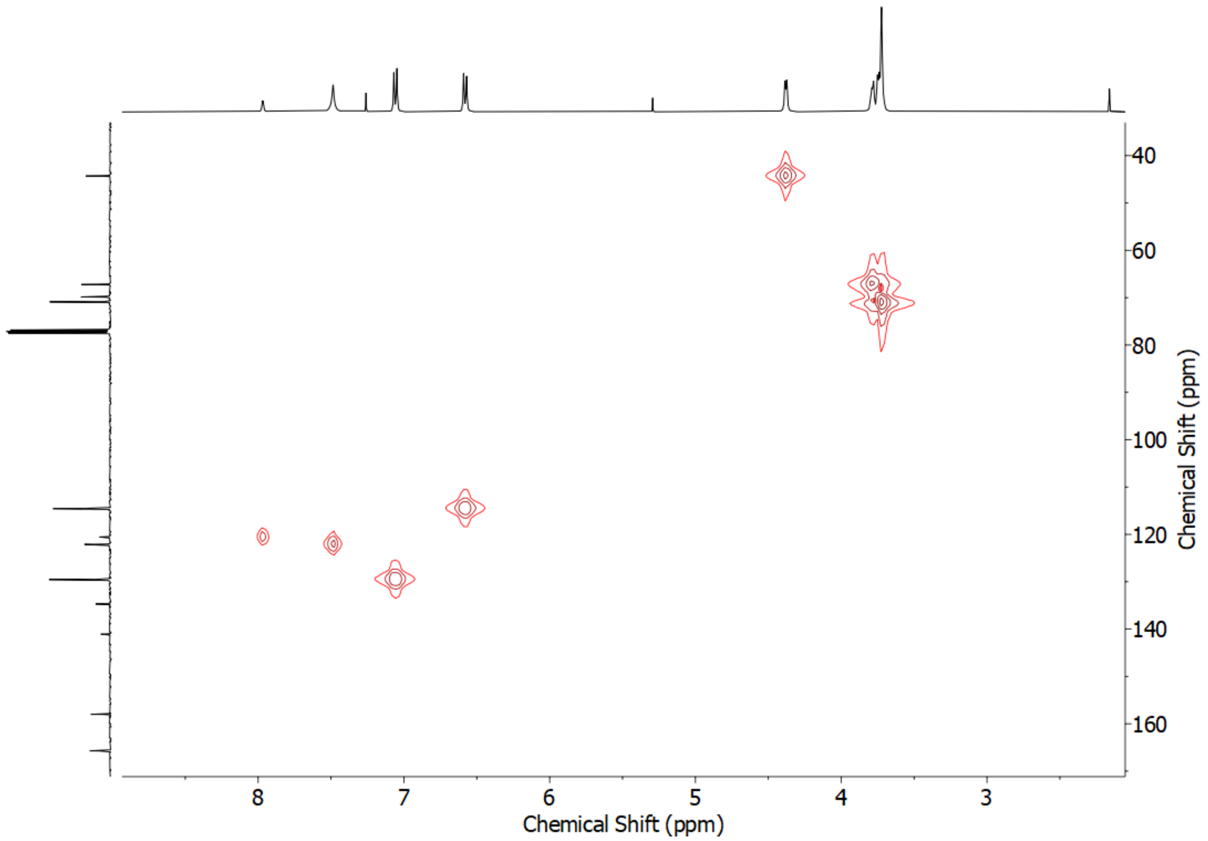

Figure S6 HSQC NMR ($CDCl_3$) of $\mathbf{3^{N3}}$

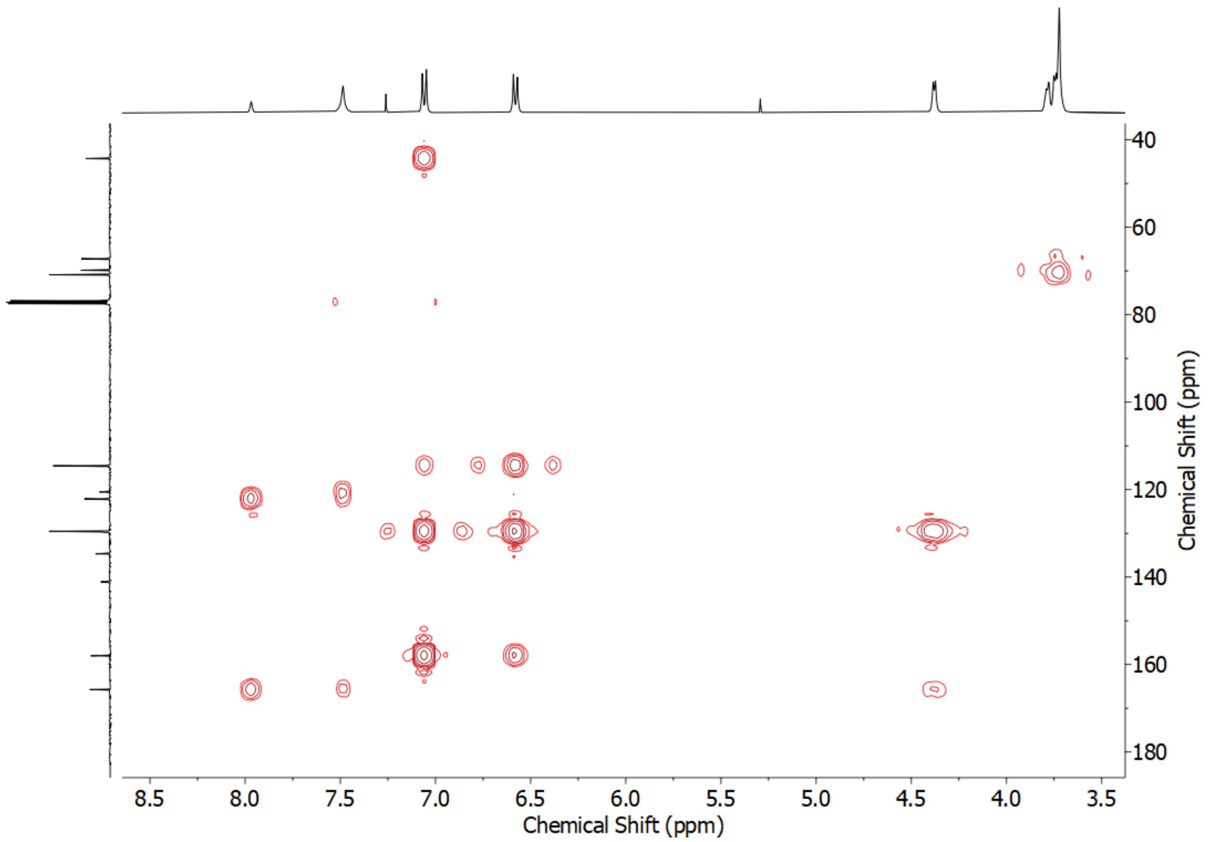

Figure S7 HMBC NMR ($CDCl_3$) of $\mathbf{3^{N3}}$

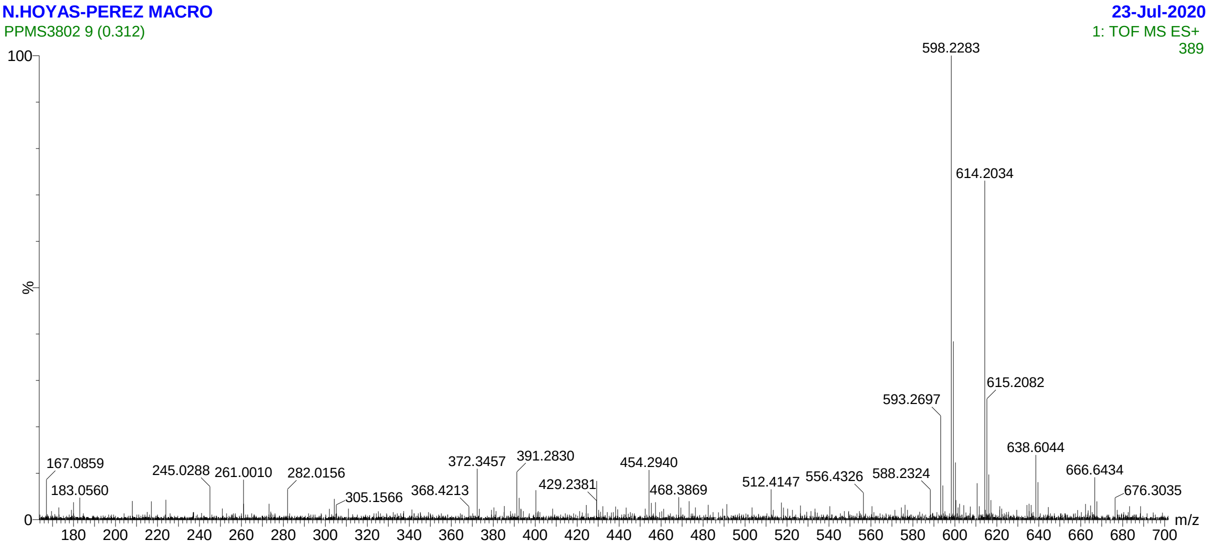

Figure S8 HR-ESI-MS of $\mathbf{3^{N3}}$.

4^N3^

^{1}H NMR (400 MHz, $CDCl_3$) δ: 8.06 (s, 2H, H_b), 7.80 (s, 4H, H_a), 7.39 (t, J = 4.9 Hz, 4H, H_{NH}), 6.92 (d, J = 8.6 Hz, 8H, H_d), 6.30 (d, J = 8.6 Hz, 8H, H_e), 4.37 (d, J = 4.7 Hz, 8H, H_c), 3.75-3.74 (m, 8H, H_f), 3.69-3.67 (m, 8H, H_g), 3.51-3.46 (m, 16H, H_h, H_i).

^{13}C NMR (101 MHz, $CDCl_3$) δ: 165.0, 157.5, 141.6, 135.8, 129.8, 129.5, 122.2, 119.6, 113.9, 70.7, 70.5, 69.7, 67.1, 44.3.

HR-ESI-MS *m/z* = 1151.4863 $[M+H]^+$ calc. 1151.4833.

M.p. 145±1 °C.

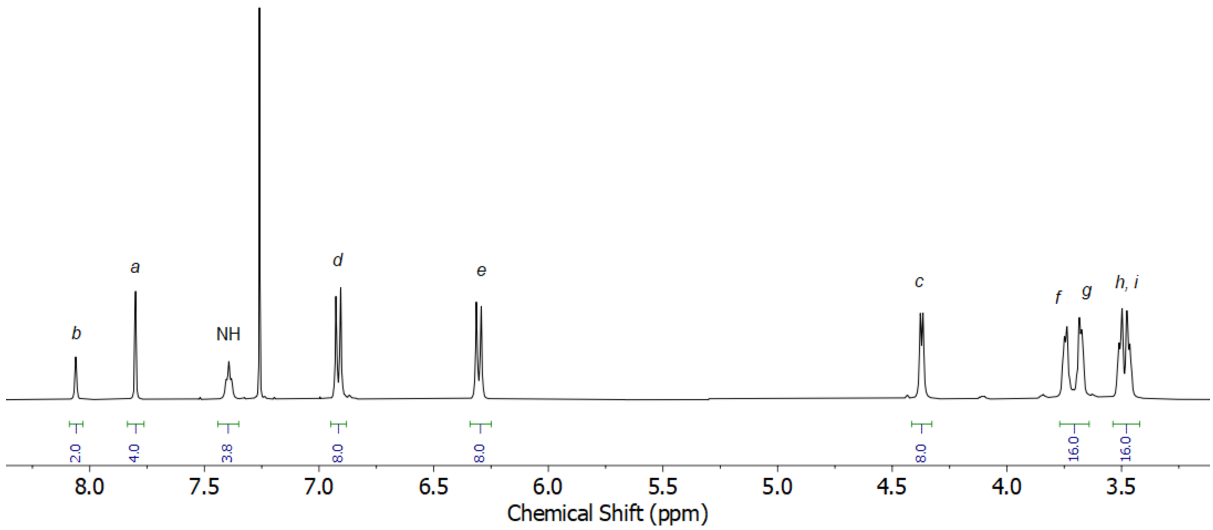

Figure S9 ^{1}H NMR ($CDCl_3$, 400 MHz) of **4^N3^**

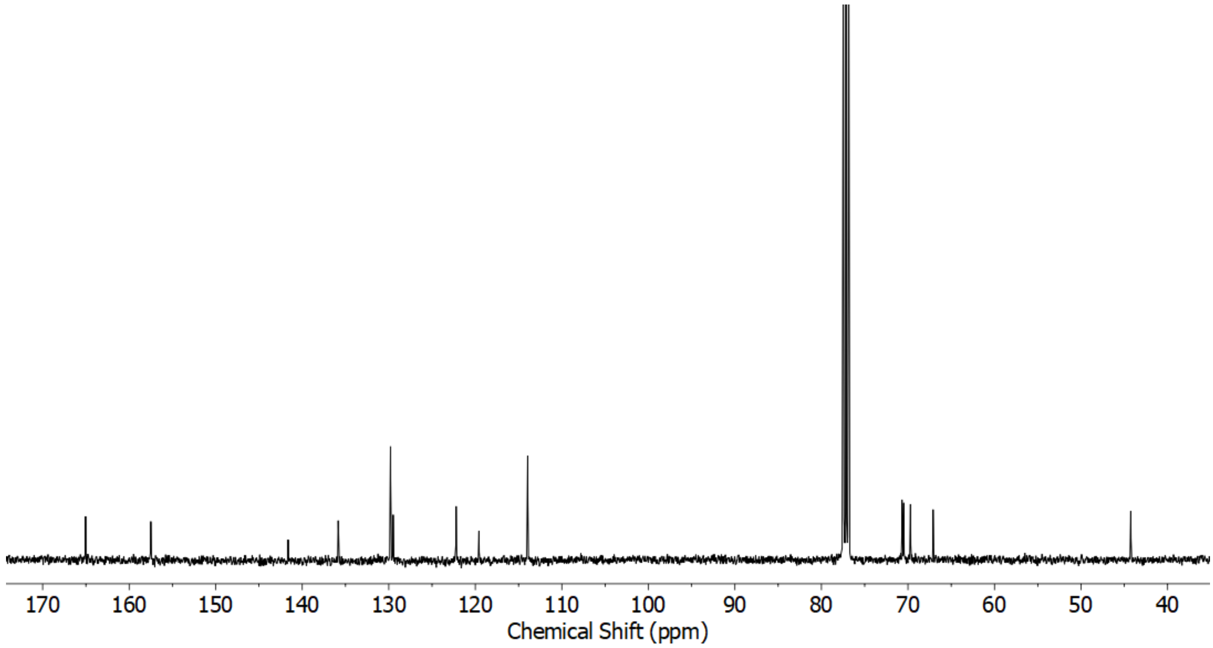

Figure S10 ^{13}C NMR ($CDCl_3$, 101 MHz) of **4^N3^**

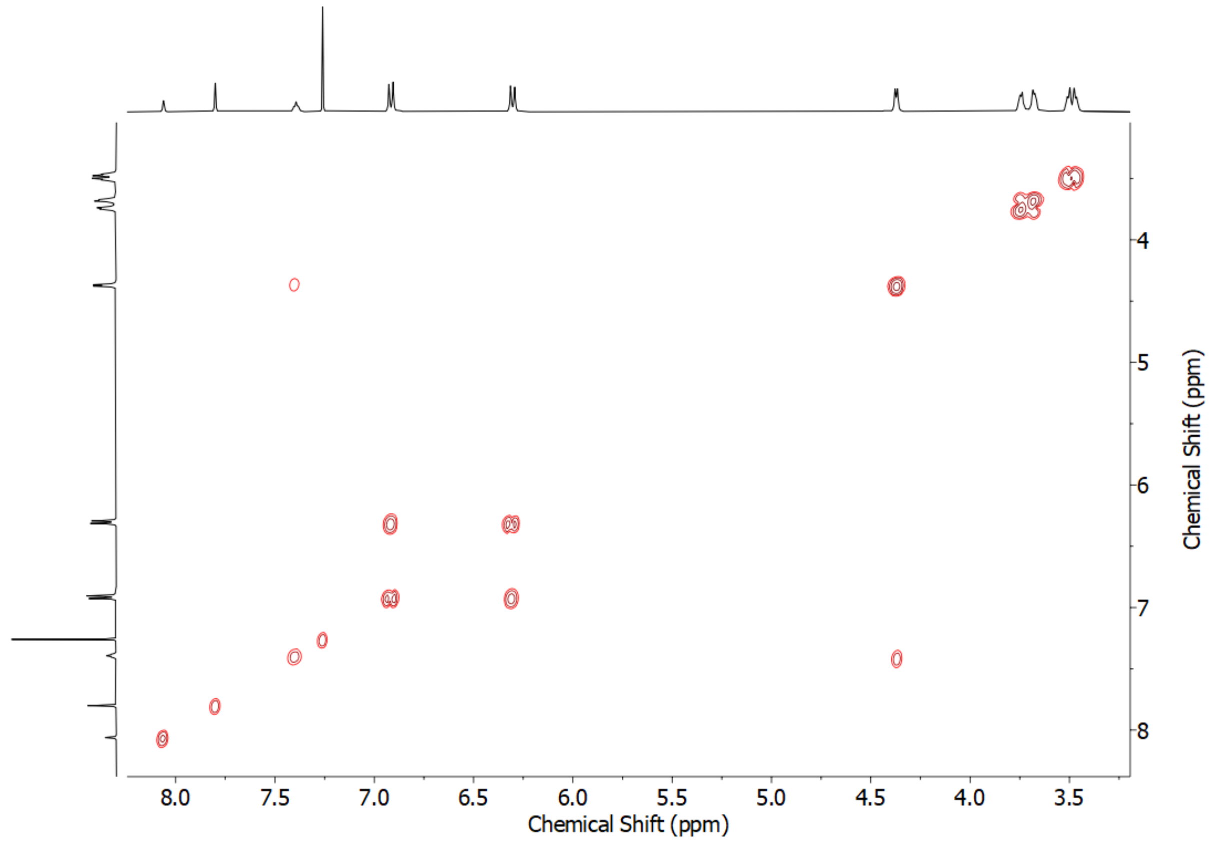

Figure S11 COSY NMR ($CDCl_3$) of **4**$^{\mathbf{N3}}$

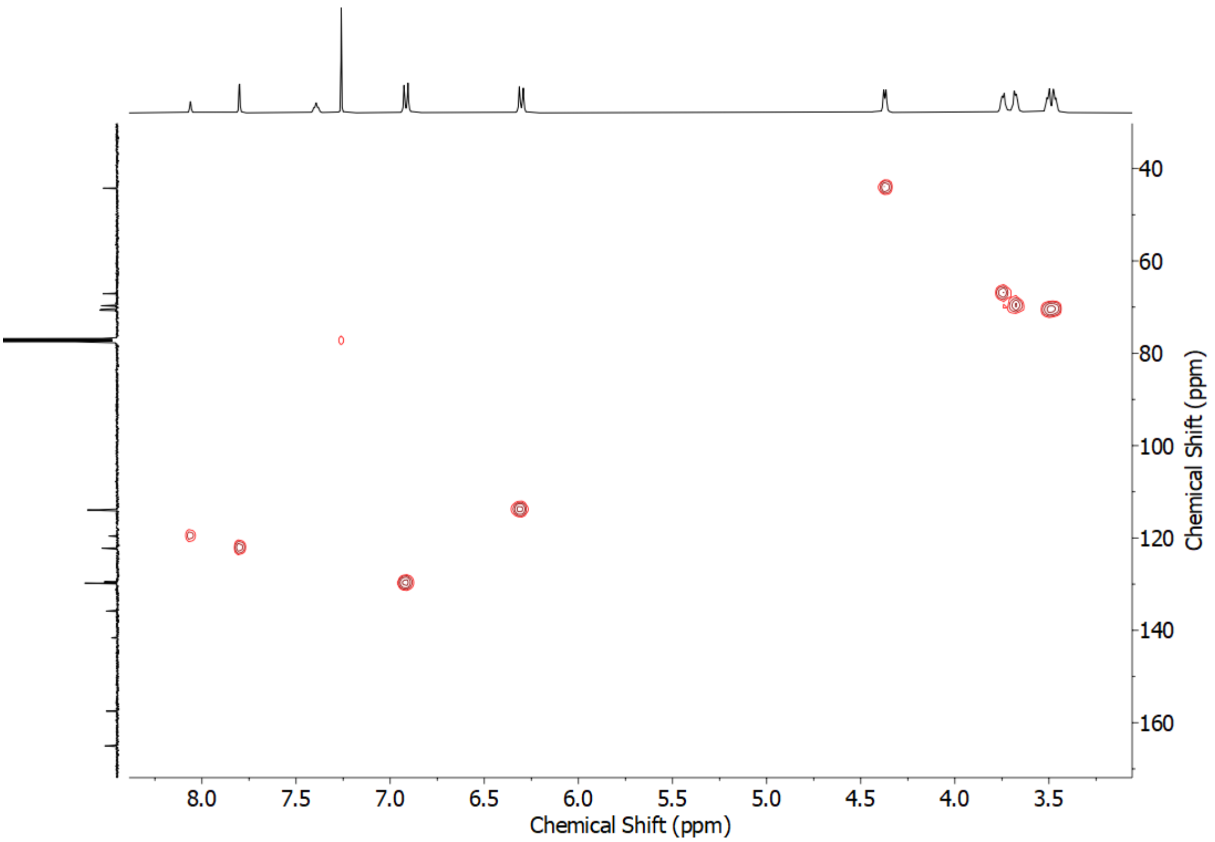

Figure S12 HSQC NMR ($CDCl_3$) of **4**$^{\mathbf{N3}}$

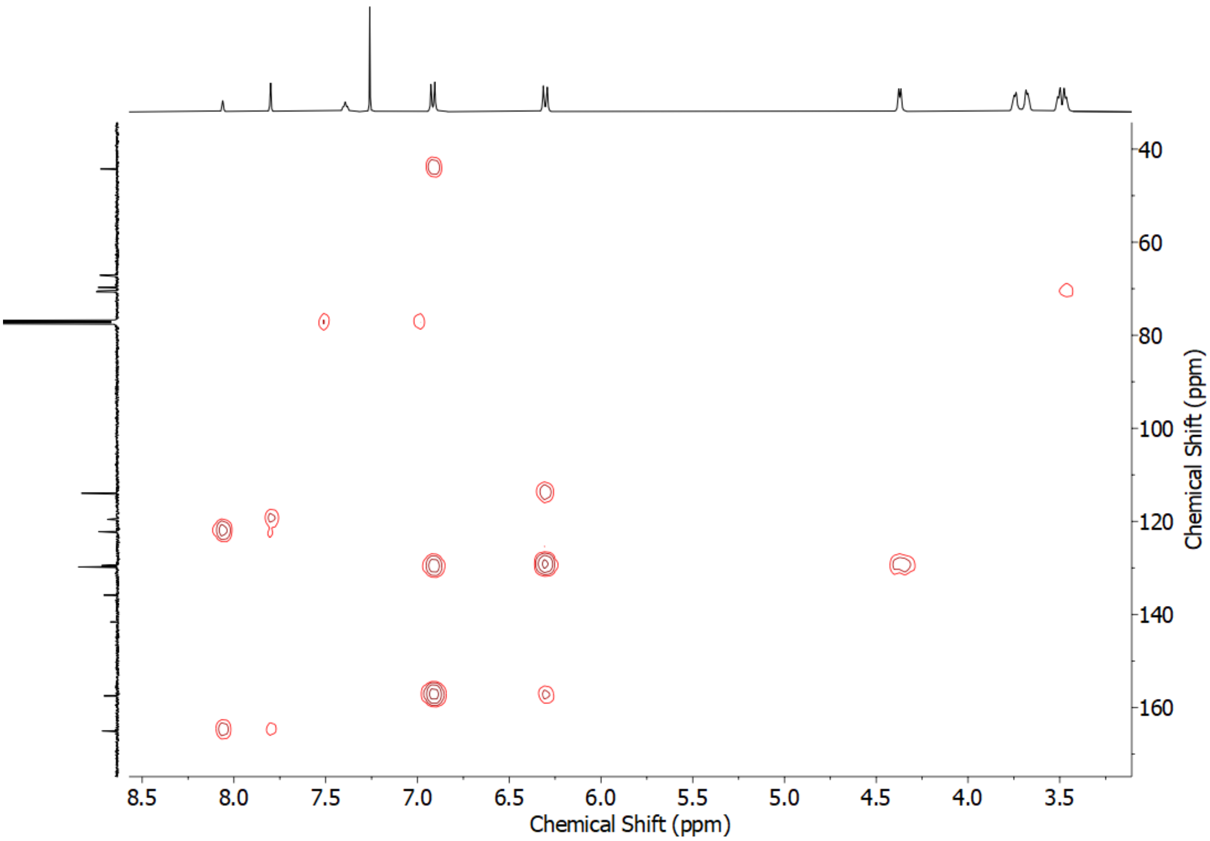

Figure S13 HMBC NMR ($CDCl_3$) of **4^N3^**

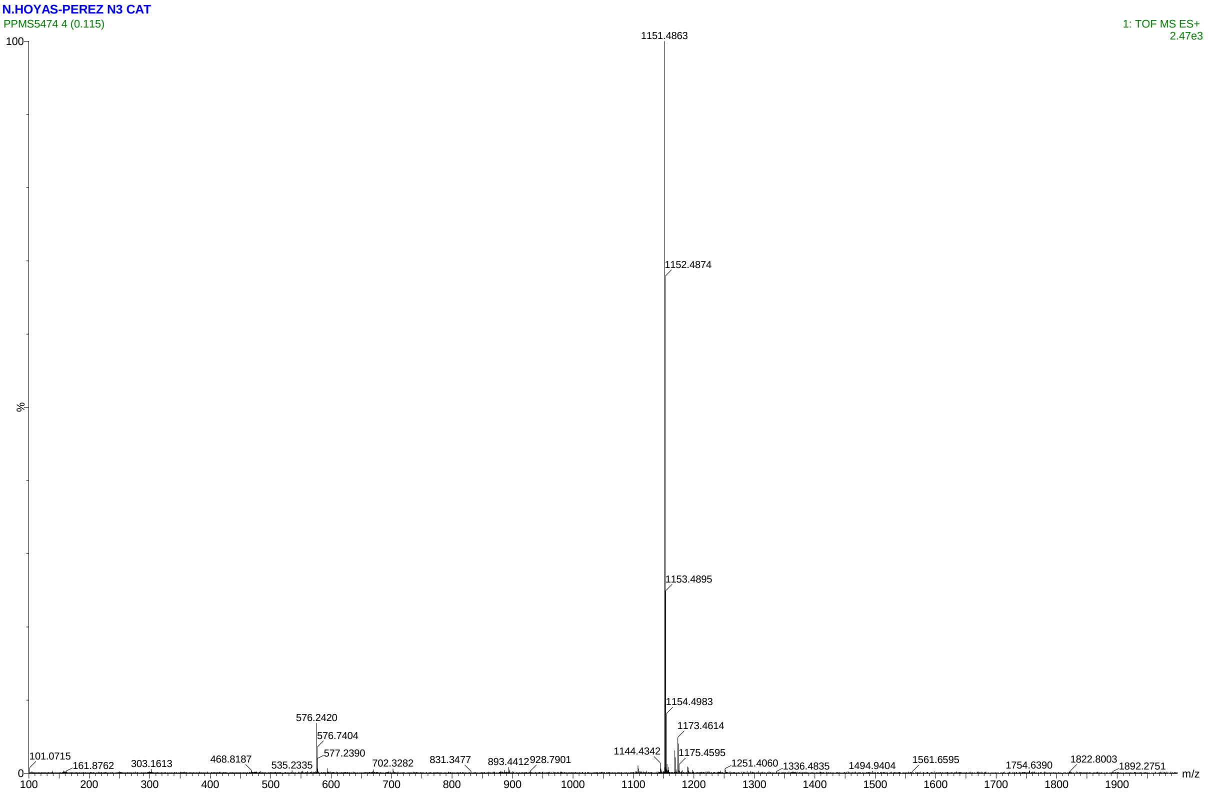

Figure S14 HR-ESI-MS of **4^N3^**.

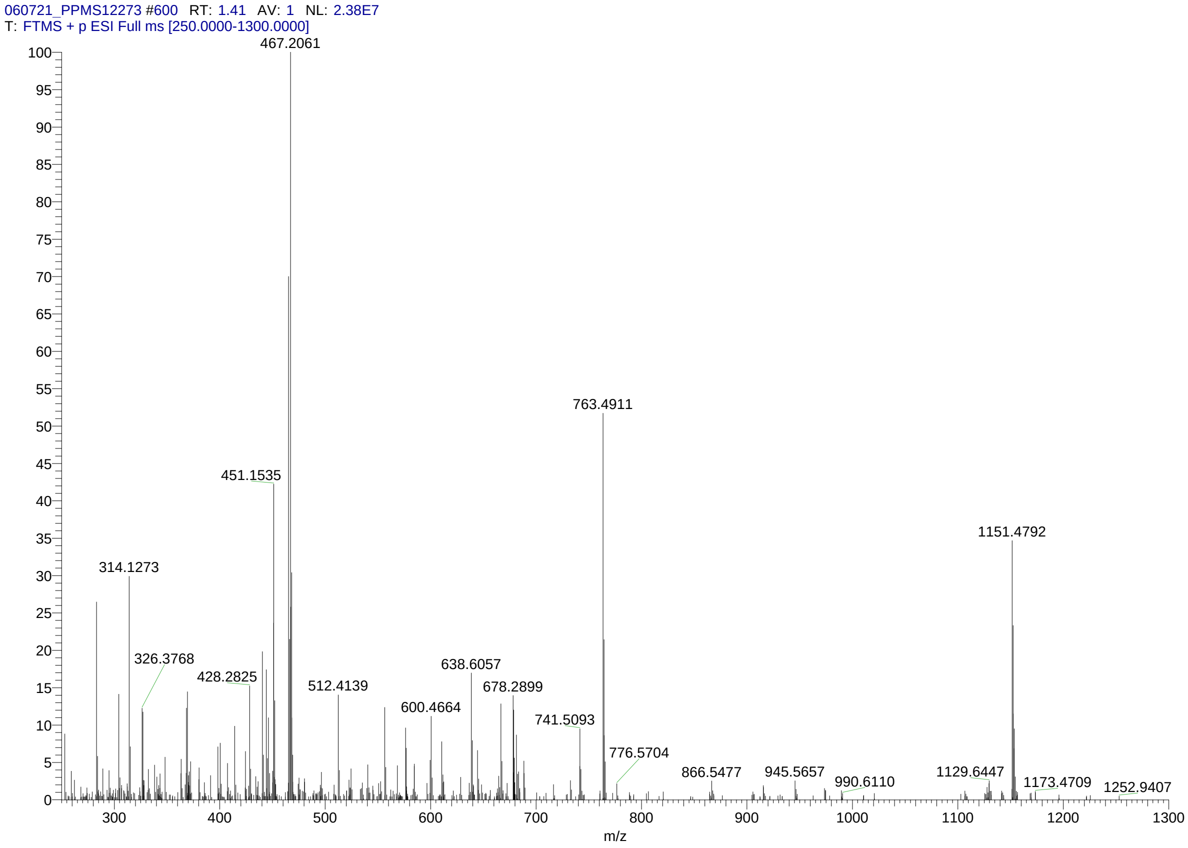

Figure S15 HR-ESI-MS of $\mathbf{4^{N3}}$. Found 1151.4792 [$\mathbf{4^{N3}}$+H]$^+$ calc. 1151.4833.

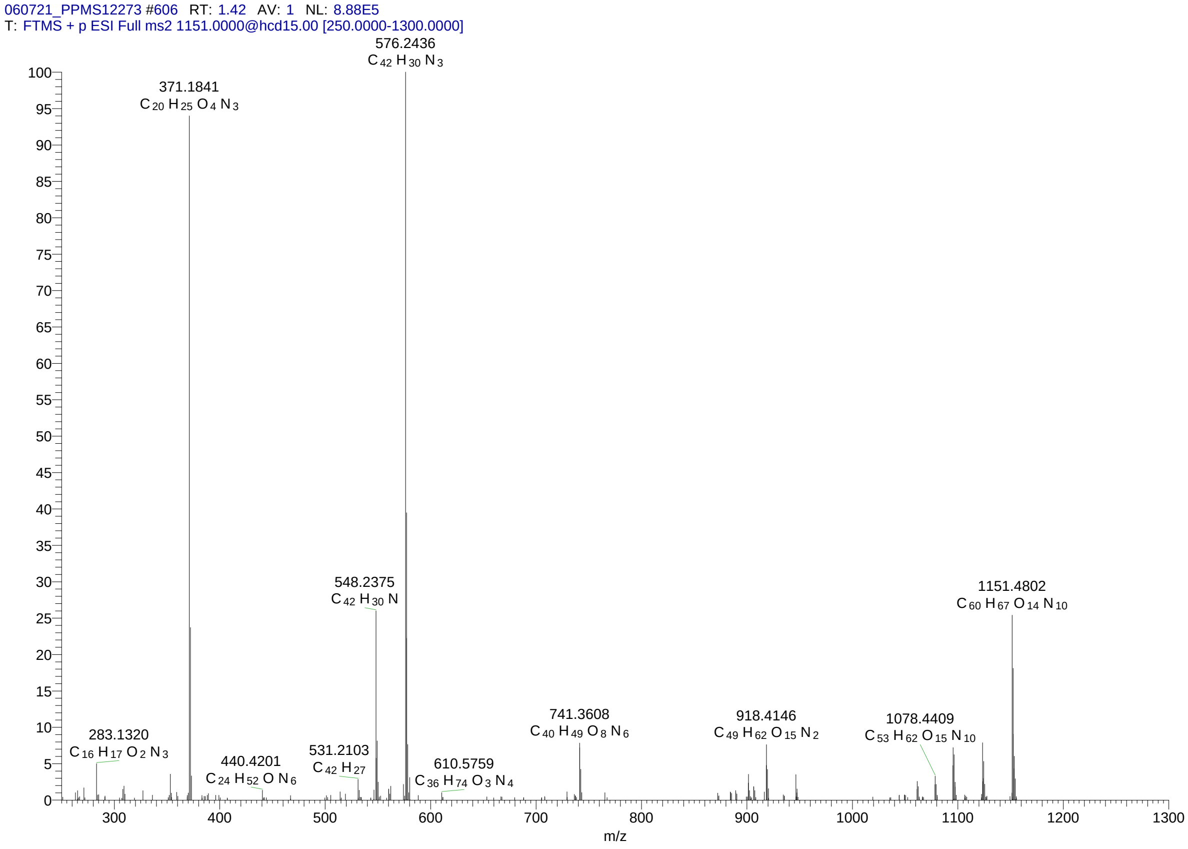

Figure S16 HR-ESI-MSMS of *m/z* = 1151.4792 peak. Found 576.2436 [$\mathbf{3^{N3}}$+H]$^+$ calc. 576.2453; found 548.2375 [$\mathbf{3^{N3}}$-N_2+H]$^+$ calc. 548.2392.

General CuAAC Procedure

A solution of **3^{N3}**/**4^{N3}** (0.025mmol), alkyne derivate (1.5 eq. per azide), $[Cu(CH_3CN)](PF_6)$ (0.5 eq. per azide), TBTA (0.5 eq. per azide) and $^{i}Pr_2NEt$ (1 eq. per azide) in 1:1 CH_2Cl_2/MeOH was stirred at rt overnight. CH_2Cl_2 (10 mL) was added and the organic phase washed with EDTA solution (2 × 10mL) and brine (2 × 10mL), dried ($MgSO_4$) and the solvent removed *in vacuo*. The products were obtained following purification by column chromatography on silica gel.

Synthesis of 3^{Ph}

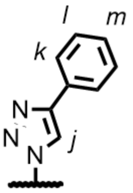

Prepared according to general procedure with **3^{N3}** (14.3 mg, 0.025 mmol, 1 eq.), phenylacetylene (3.8 mg, 0.037 mmol, 1.5 eq.), $[Cu(CH_3CN)_4](PF_6)$ (6.6 mg, 0.012 mmol, 0.5 eq.), TBTA (4.7 mg, 0.012 mmol, 0.5 eq.), and purified by column chromatography on silica (3:7 acetone/CH_2Cl_2) to give **3^{Ph}** as a white solid (14.6 mg, 86%).

^{1}H NMR (500 MHz, *d*$_6$-DMSO) δ: 9.55 (s, 1H, H$_j$), 9.02 (t, J = 5.6 Hz, 2H, H$_{NH}$), 8.52 (d, J = 1.4 Hz, 2H, H$_a$), 8.24 (t, J = 1.5 Hz, 1H, H$_b$), 8.00 (dd, J = 8.3, 1.3 Hz, 2H, H$_k$), 7.52 (app. t, J = 7.7 Hz, 2H, H$_l$), 7.40 (m, 1H, H$_m$), 7.28 (d, J = 8.7 Hz, 4H, H$_d$), 6.90 (d, J = 8.7 Hz, 4H, H$_e$), 4.45 (d, J = 5.6 Hz, H$_c$), 4.07-4.05 (m, 4H, H$_f$), 3.71-3.69 (m, 4H, H$_g$), 3.56-3.50 (m, 8H, H$_h$, H$_i$).

^{13}C NMR (126 MHz, *d*$_6$-DMSO) δ: 165.0, 157.6, 147.6, 136.8, 136.7, 130.9, 130.1, 129.3, 129.1, 128.4, 125.4 (×2), 121.0, 119.9, 114.3, 70.0, 69.9, 68.8, 67.2, 42.6.

HR-ESI-MS *m/z* = 678.2936 [M+H]$^+$ calc. 678.2923.

M.p. 139±1 °C.

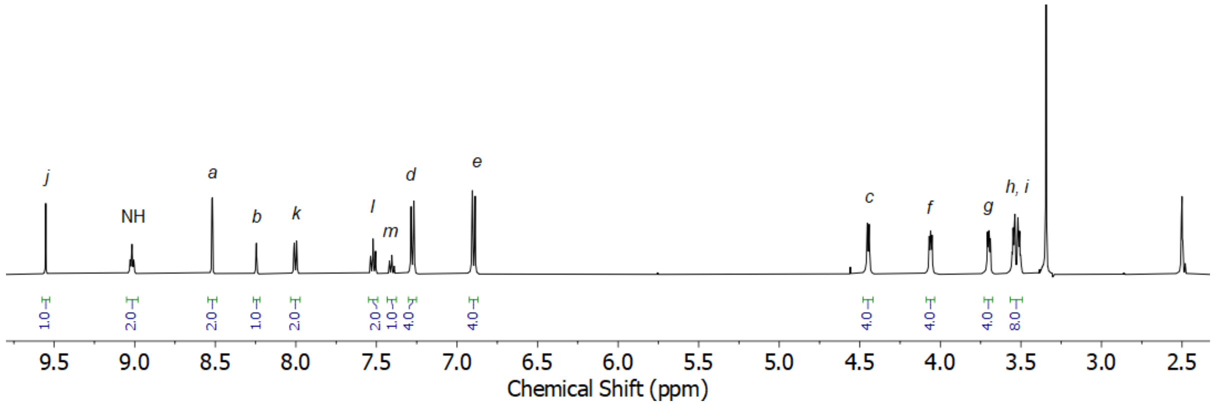

Figure S17 ^{1}H NMR (*d*$_6$-DMSO, 500 MHz) of **3^{Ph}**

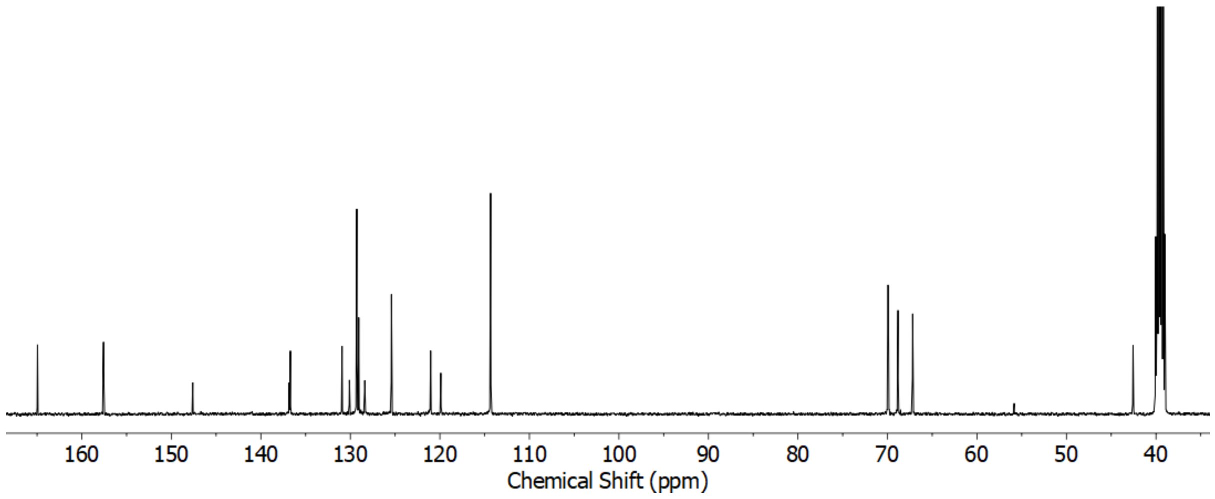

Figure S18 ^{13}C NMR (d_6-DMSO, 126 MHz) of **3**$^{\mathbf{Ph}}$

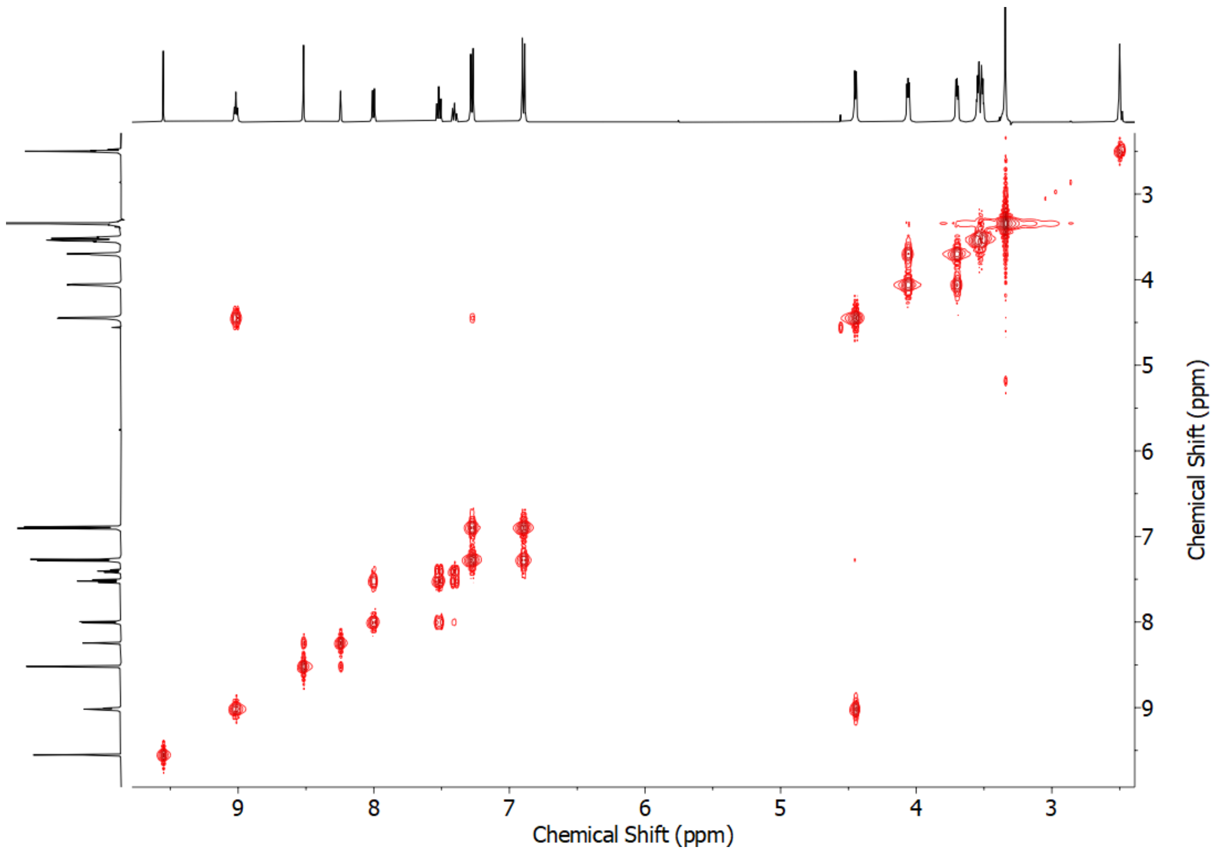

Figure S19 COSY NMR (d_6-DMSO) of **3**$^{\mathbf{Ph}}$

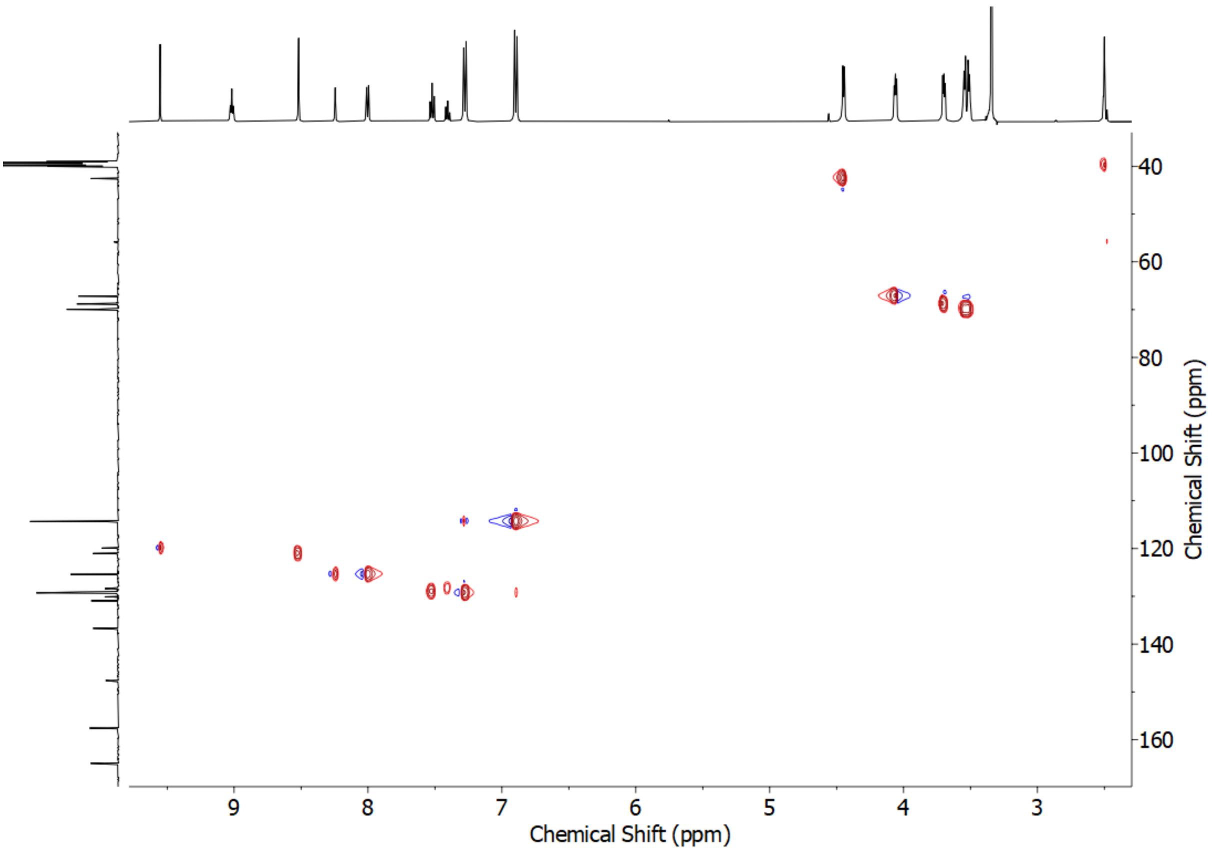

Figure S20 HSQC NMR (d_6-DMSO) of **3**$^{\mathbf{Ph}}$

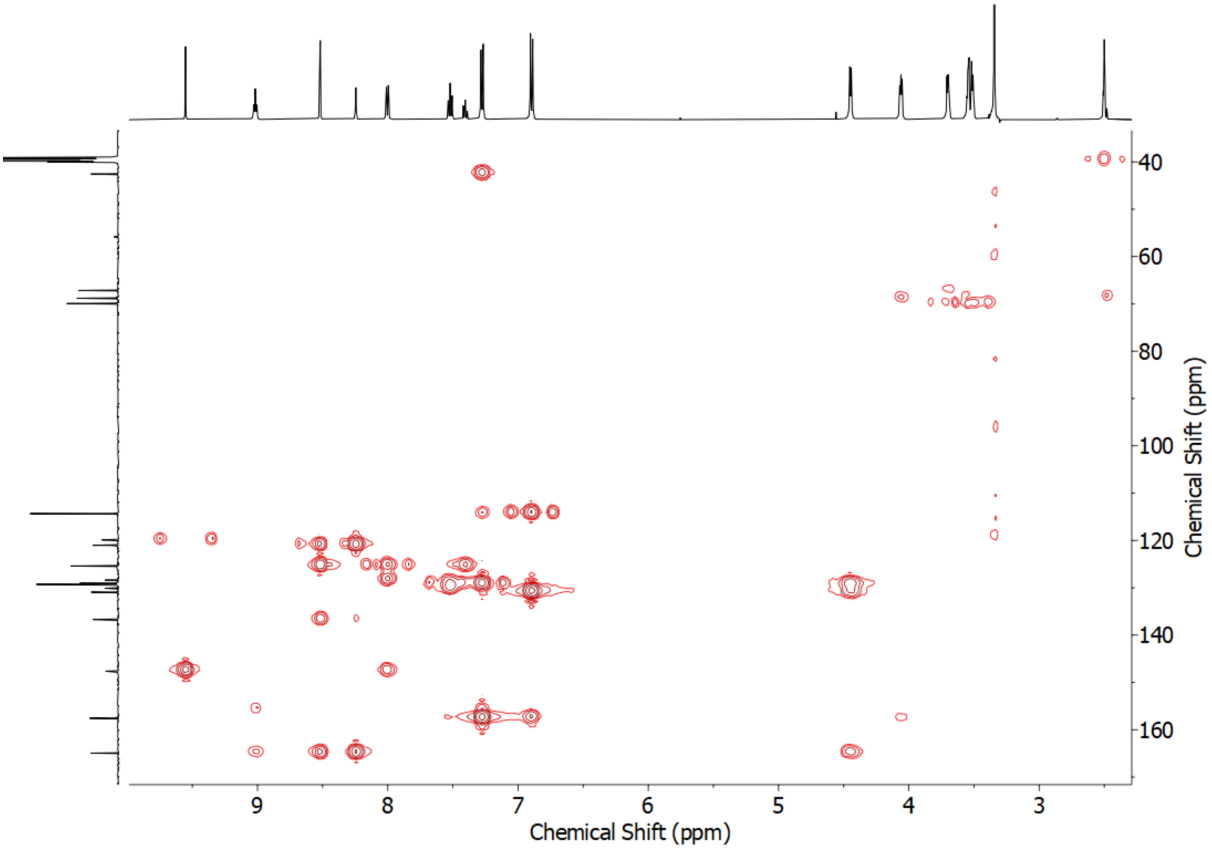

Figure S21 HMBC NMR (d_6-DMSO) of **3**$^{\mathbf{Ph}}$

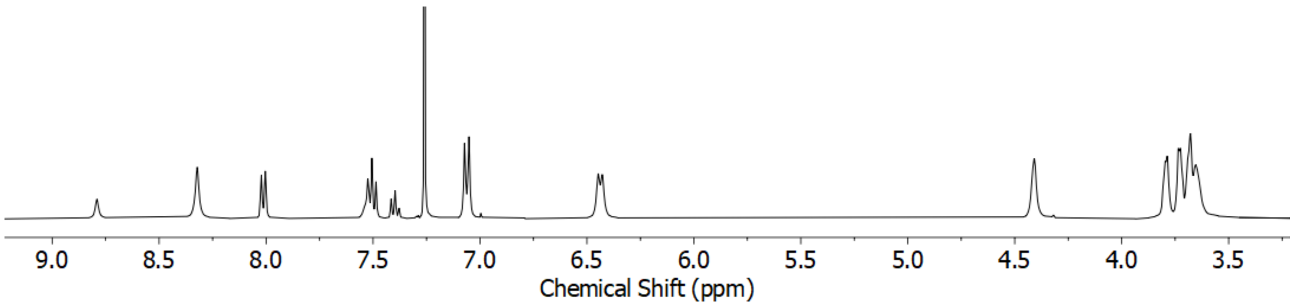

Figure S22 ^{1}H NMR ($CDCl_3$, 400 MHz) of **3^{Ph}**

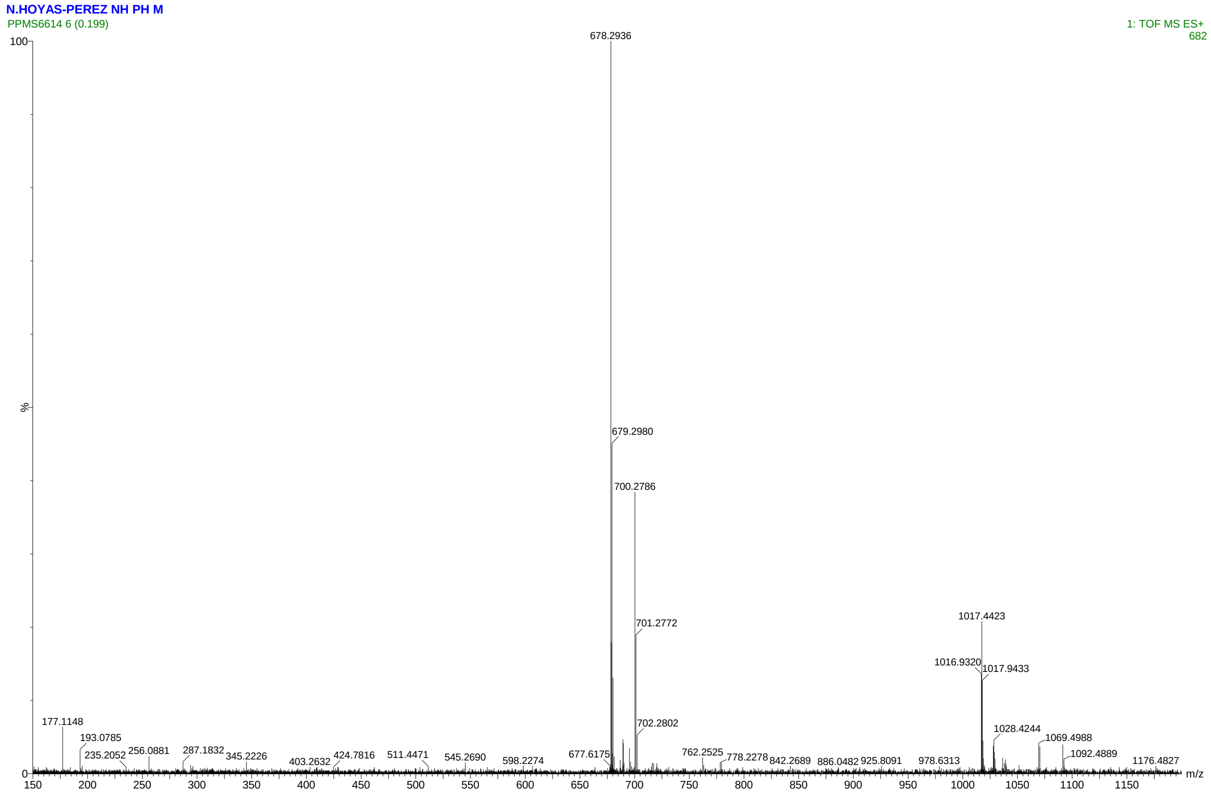

Figure S23 HR-ESI-MS of **3^{Ph}**.

Synthesis of 4Ph

Prepared according to general procedure with **4N3** (38.8 mg, 0.025 mmol, 1 eq.), phenylacetylene (7.7 mg, 0.075 mmol, 3 eq.), $[Cu(CH_3CN)_4](PF_6)$ (13.2 mg, 0.025 mmol, 1 eq.), TBTA (9.4 mg, 0.025 mmol, 1 eq.), and purified by column chromatography on silica (1:4 acetone/CH_2Cl_2) to give **4Ph** as a white solid (31.4 mg, 85%).

^{1}H NMR (400 MHz, $CDCl_3$) δ: 8.59 (br. s, 2H, H_b), 8.34 (br. m, 6H, H_a, H_j), 7.91-7.87 (m, 8H, H_{NH}, H_k), 7.50 (app. t, J = 7.5 Hz, 4H, H_l), 7.42 (m, 2H, H_m), 7.01 (br. d, J = 8.1 Hz, 8H, H_d), 6.54 (br. m, 8H, H_e), 4.39 (br. s, 8H, H_c), 3.69 (br. s, 8H, H_f), 3.52-3.48 (br. m, 16H, H_g, H_h), 3.36 (br. s, 8H, H_i).

^{13}C NMR (101MHz, $CDCl_3$) δ: 165.0, 157.8, 148.7, 137.0, 135.9, 130.0, 129.8, 129.3, 129.0, 125.8 (×2), 124.8, 122.8, 118.4, 114.1, 70.6, 70.4, 69.6, 66.9, 44.0.

HR-ESI-MS *m/z* = 1355.5800 $[M+Na]^+$ calc. 1355.5772.

M.p. 151±1 °C.

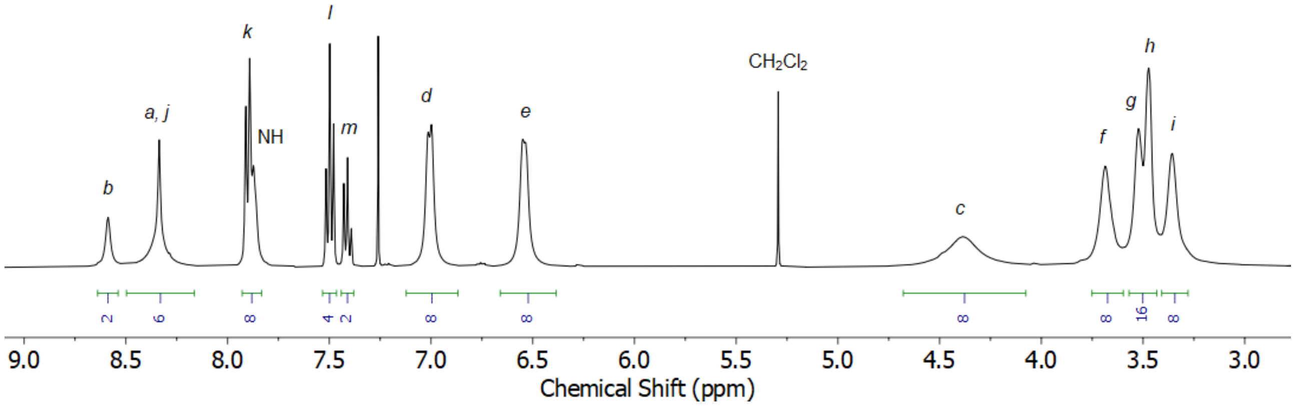

Figure S24 ^{1}H NMR ($CDCl_3$, 500 MHz) of **4Ph**

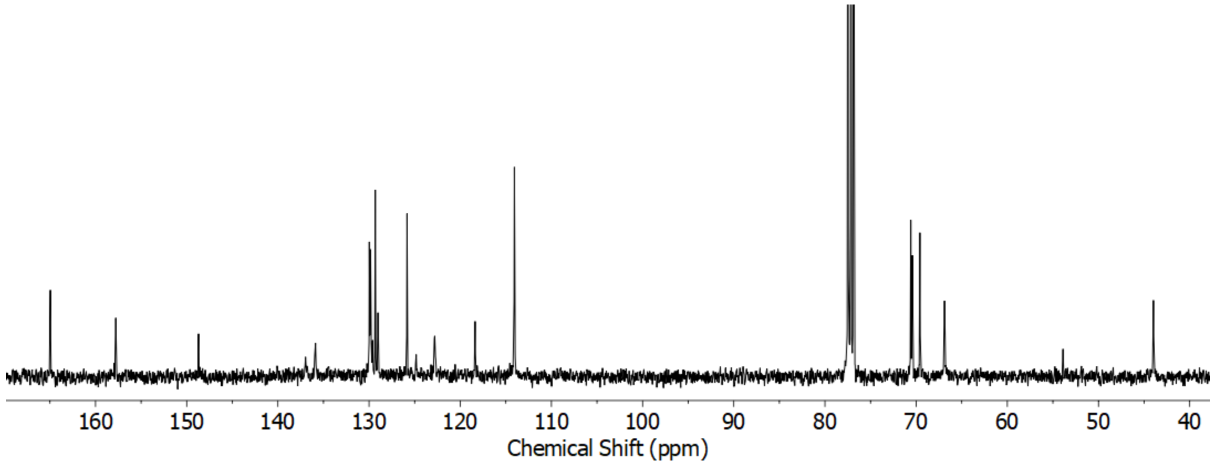

Figure S25 ^{13}C NMR ($CDCl_3$, 126 MHz) of **4Ph**

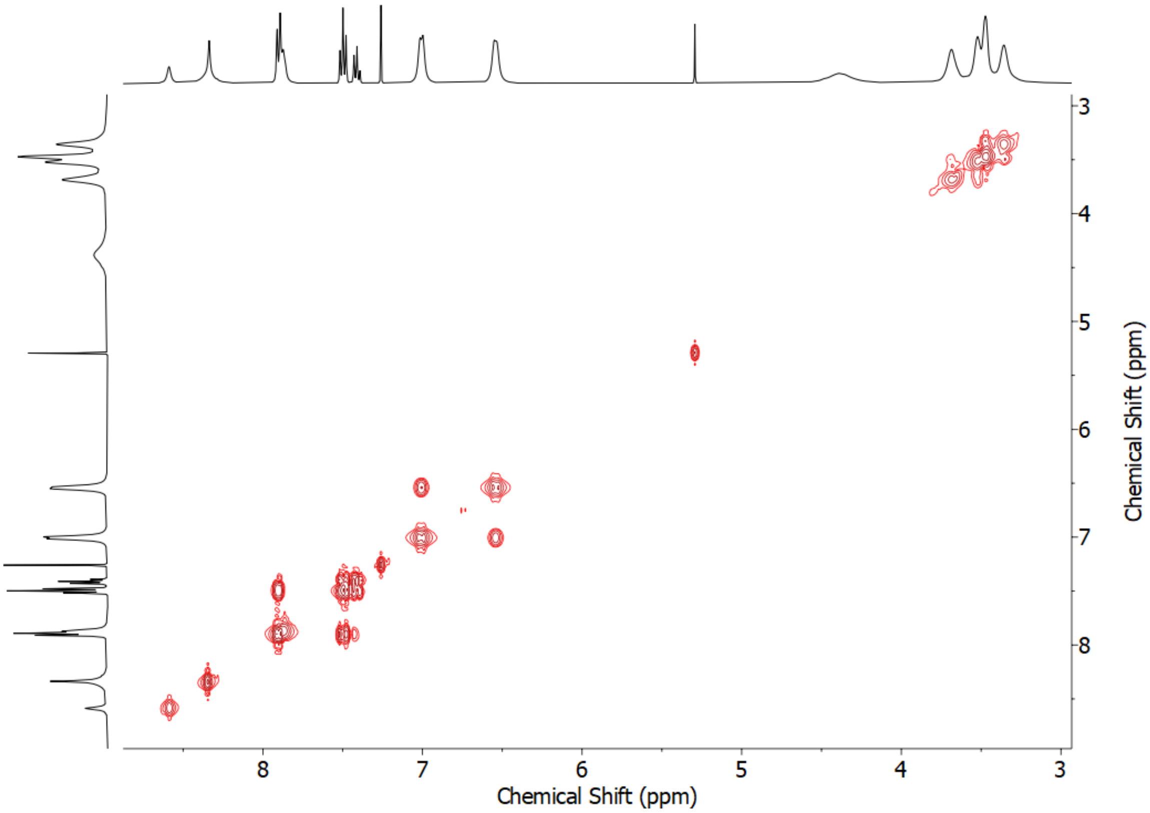

Figure S26: COSY NMR ($CDCl_3$ 500 MHz) of **4**^{Ph}

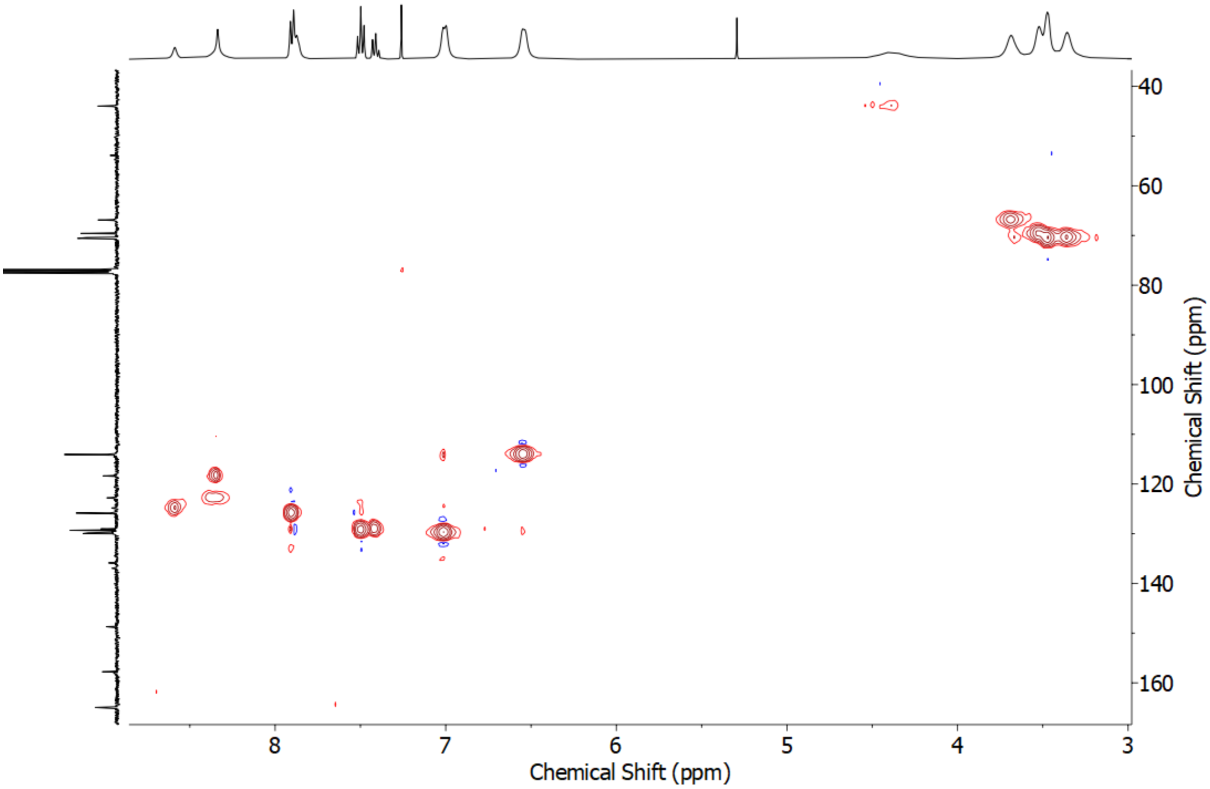

Figure S27 HSQC NMR ($CDCl_3$) of **4**^{Ph}

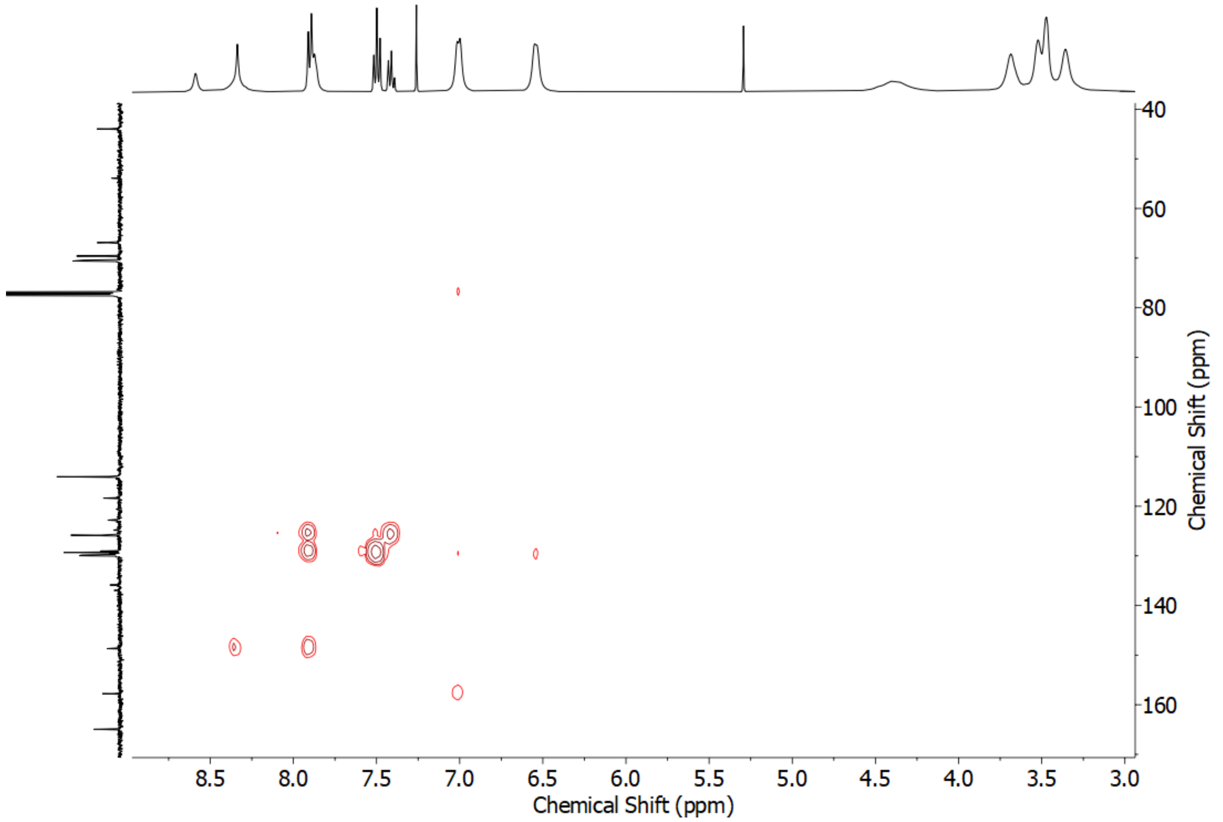

Figure S28 HMBC NMR ($CDCl_3$) of **4^{Ph}**

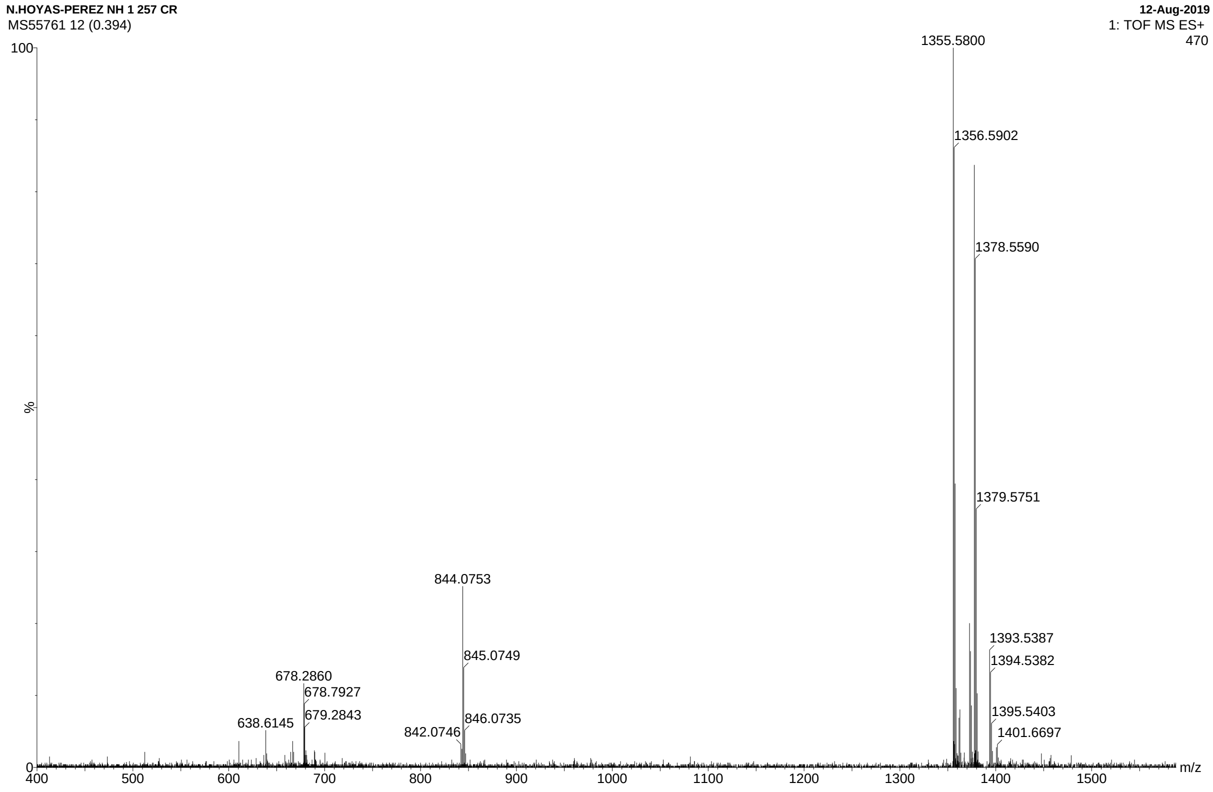

Figure S29 HR-ESI-MS of **4^{N3}**.

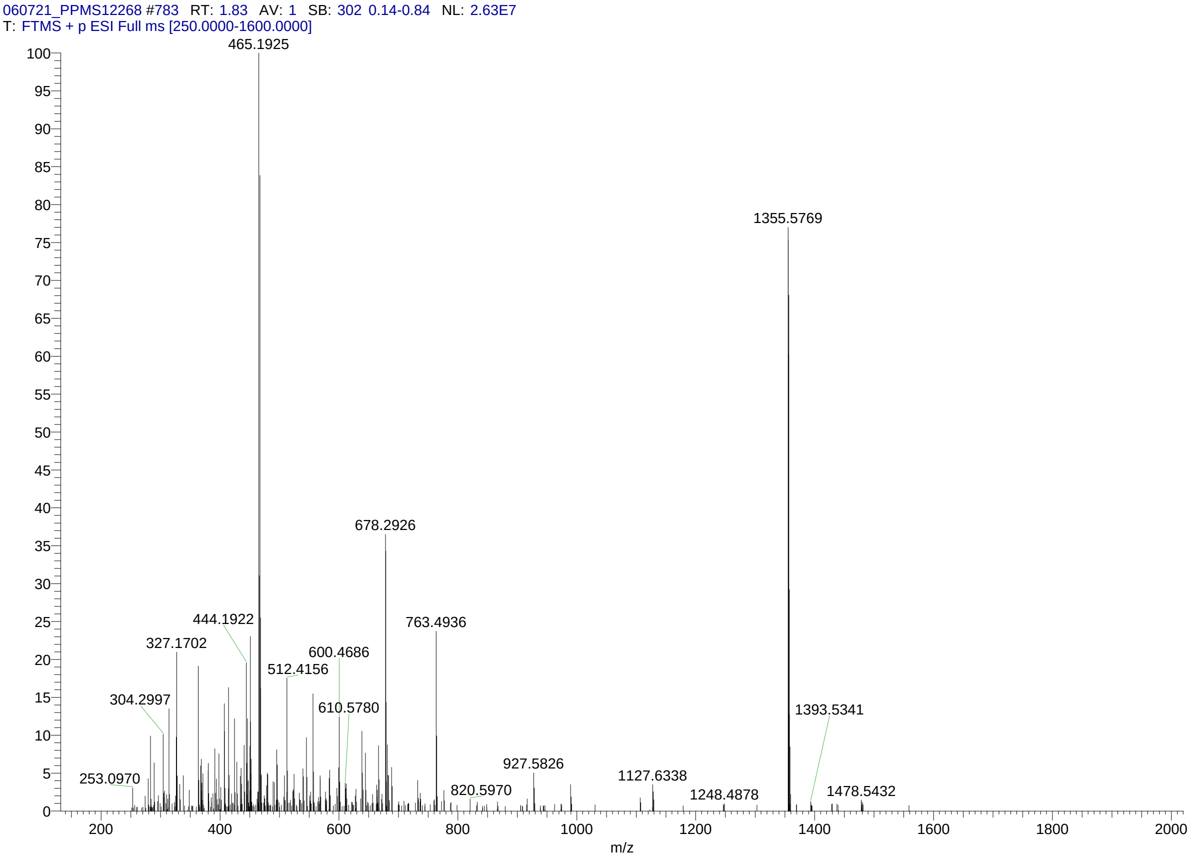

Figure S30 HR-ESI-MS of **4^{Ph}**. Found 1355.5769 [**4^{N3}**+H]$^+$ calc. 1355.5772.

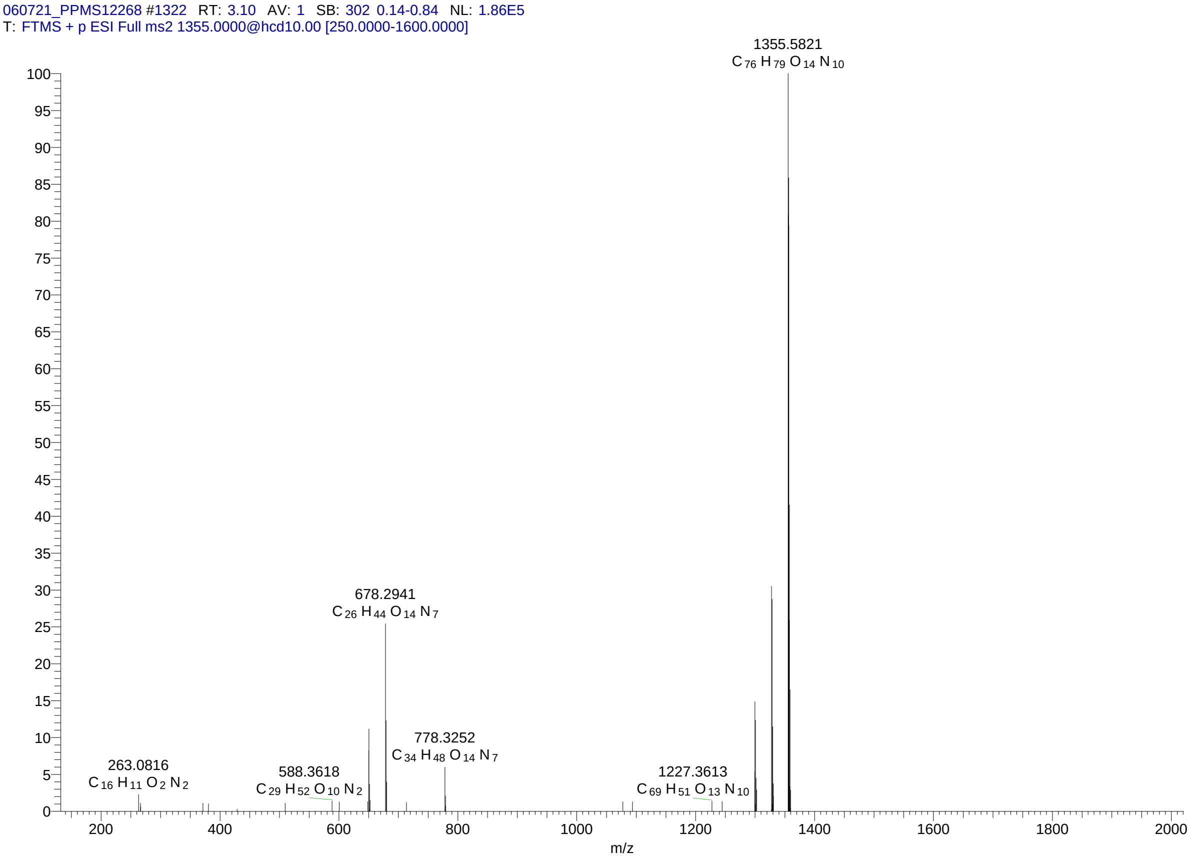

Figure S31 HR-ESI-MSMS of *m/z* = 1355.5769 peak. Found 678.2941 [**3^{Ph}**+H]$^+$ calc. 678.2923.

Synthesis of 3^{Py}

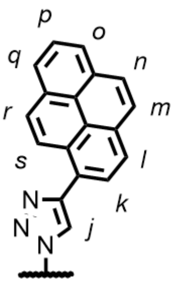

Prepared according to general procedure with $\mathbf{3^{N3}}$ (14.3 mg, 0.025 mmol, 1 eq.), 1-ethynylpyrene (8.4 mg, 0.037 mmol, 1.5 eq.), $[Cu(CH_3CN)_4](PF_6)$ (6.6 mg, 0.012 mmol, 0.5 eq.), TBTA (4.7 mg, 0.012 mmol, 0.5 eq.), and purified by column chromatography on silica (3:7 acetone/CH_2Cl_2) to give $\mathbf{3^{Py}}$ as a yellow solid (8.2 mg, 41%).

^{1}H NMR (400 MHz, *d*$_6$-DMSO) δ: 9.68 (s, 1H, H_j), 9.07 (t, J = 5.6 Hz, 2H, H_{NH}), 9.00 (d, J = 9.3 Hz, 1H, H_r/H_s), 8.66 (d, J = 1.4 Hz, 2H, H_a), 8.51 (d, J = 8.0 Hz, 1H, H_k), 8.45 (d, J = 8.0 Hz, 1H, H_l), 8.37 (app. d, J = 7.7 Hz, 2H, H_o, H_p), 8.33 (d, J = 9.4 Hz, 1H, H_r/H_s), 8.29-8.27 (m, 3H, H_b, H_m, H_n), 8.14 (t, J = 7.6 Hz, 1H, H_p), 7.29 (d, J = 8.7 Hz, 4H, H_d), 6.91 (d, J = 8.7 Hz, 4H, H_e), 4.47 (d, J = 5.5 Hz, 4H, H_c), 4.06 (m, 4H, H_f), 3.70 (m, 4H, H_g), 3.56-3.50 (m, 8H, H_h, H_i).

^{13}C NMR (101 MHz, *d*$_6$-DMSO) δ: 165.0, 157.6, 147.5, 136.9, 136.8, 131.0, 130.4, 129.3, 128.3, 128.0, 127.7, 127.4, 127.3, 126.6, 125.7, 125.6, 125.4, 125.2, 124.9, 124.6, 124.3, 123.9, 123.1, 121.5, 114.3, 70.0, 69.9, 68.8, 67.2, 42.6 (2 signals missing due to peak overlap).

HR-ESI-MS *m/z* = 803.3358 $[M+H]^+$cal. 803.3336.

M.p. 182±1 °C.

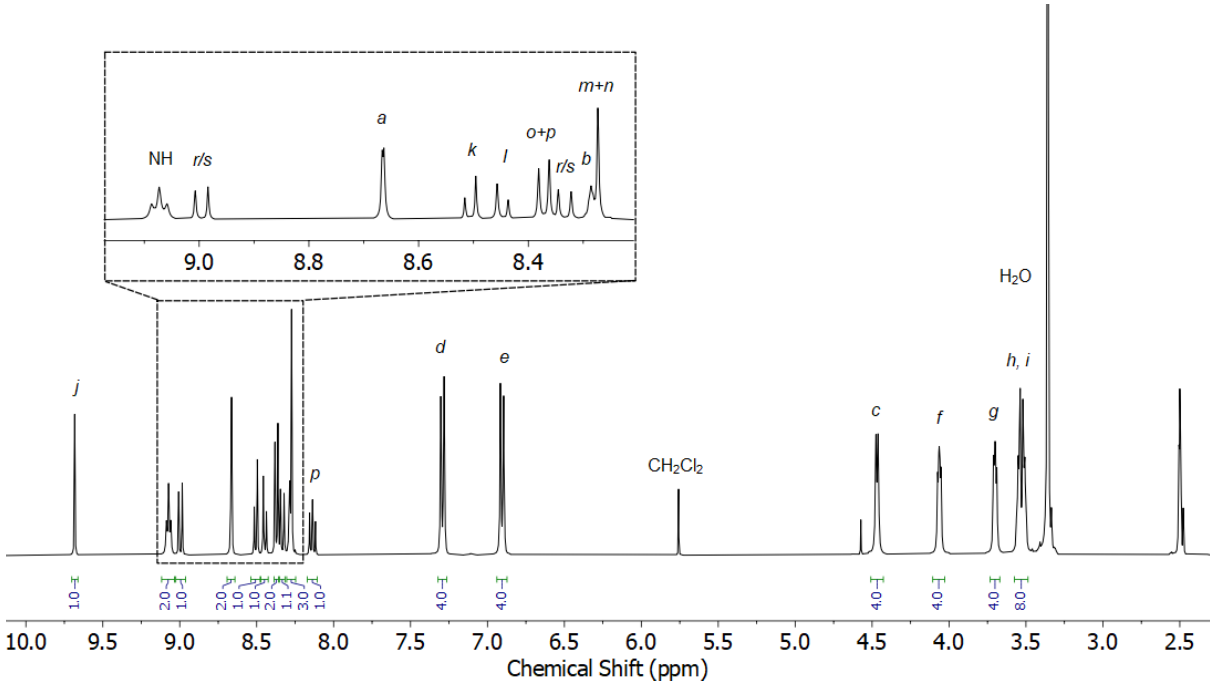

Figure S32 ^{1}H NMR (*d*$_6$-DMSO, 400 MHz) of $\mathbf{3^{Py}}$

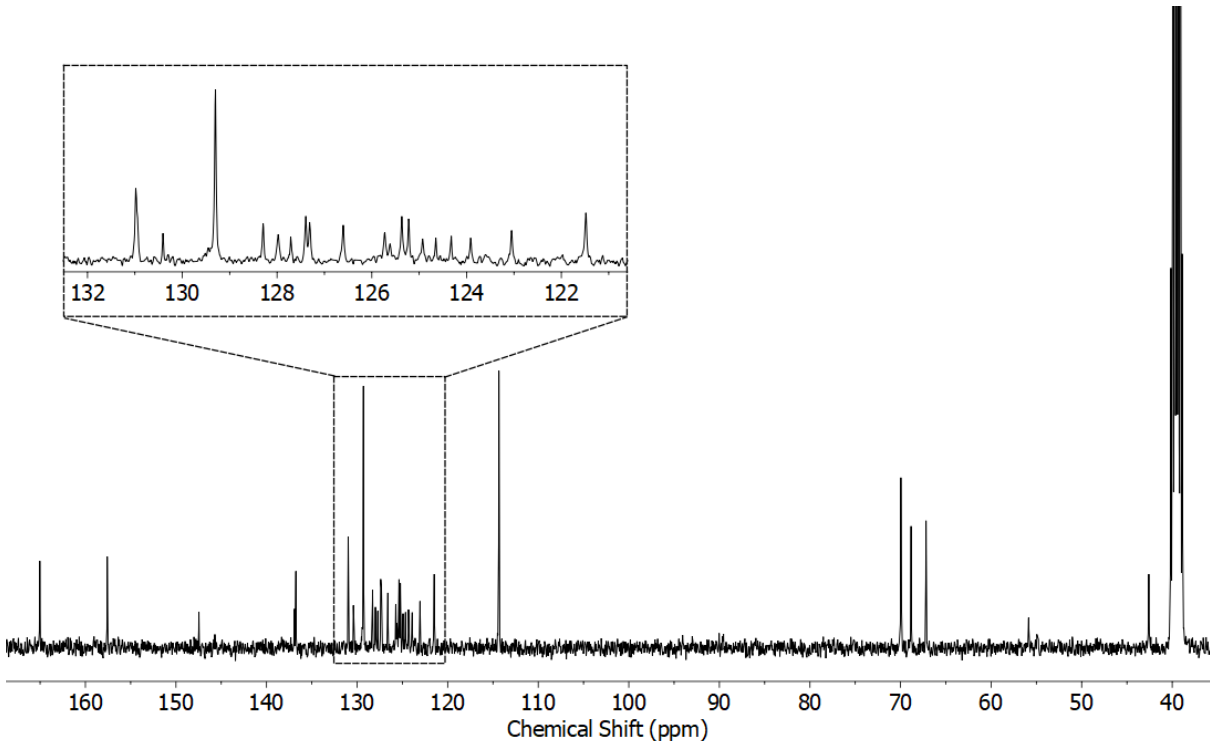

Figure S33 ^{13}C NMR (d_6-DMSO, 101 MHz) of **3**$^{\mathbf{Py}}$

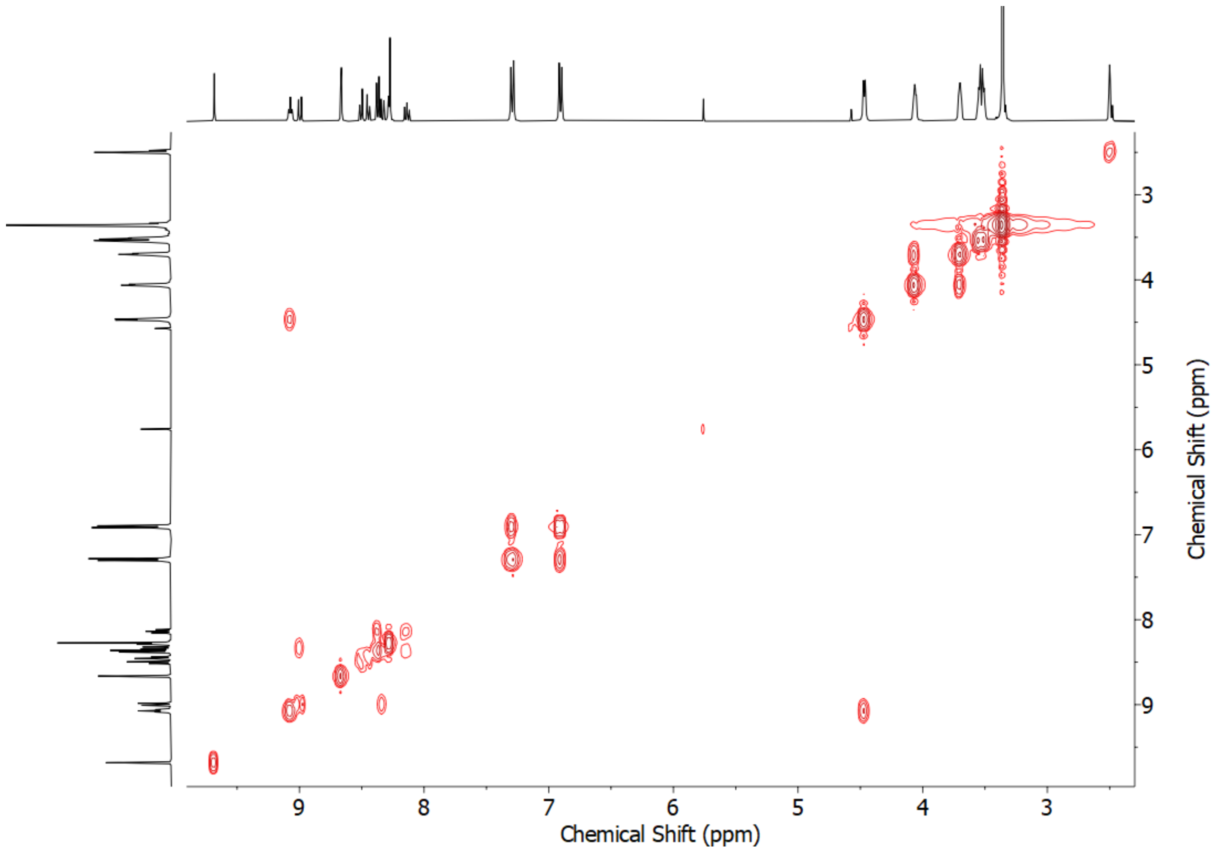

Figure S 34 COSY NMR (d_6-DMSO) of **3**$^{\mathbf{Py}}$

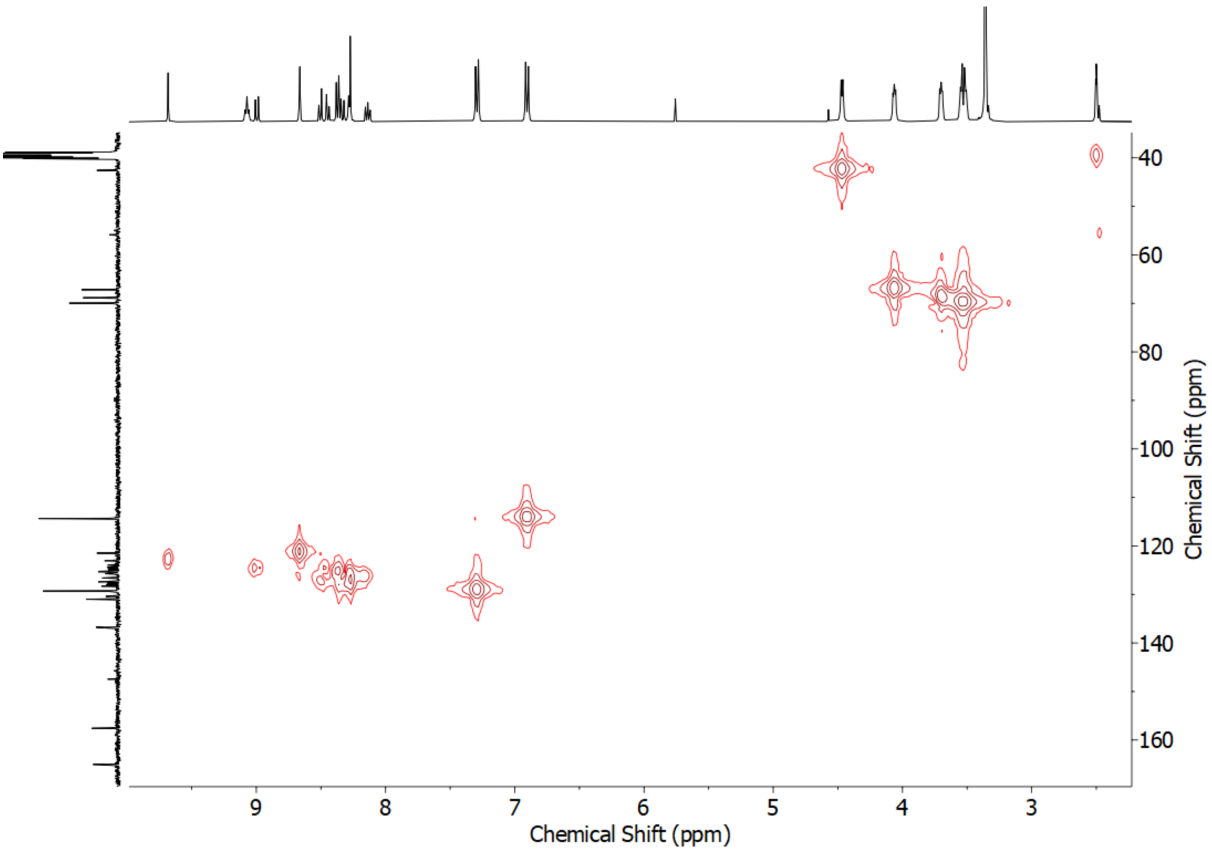

Figure S35 HSQC NMR (d_6-DMSO) of **3^{Py}**

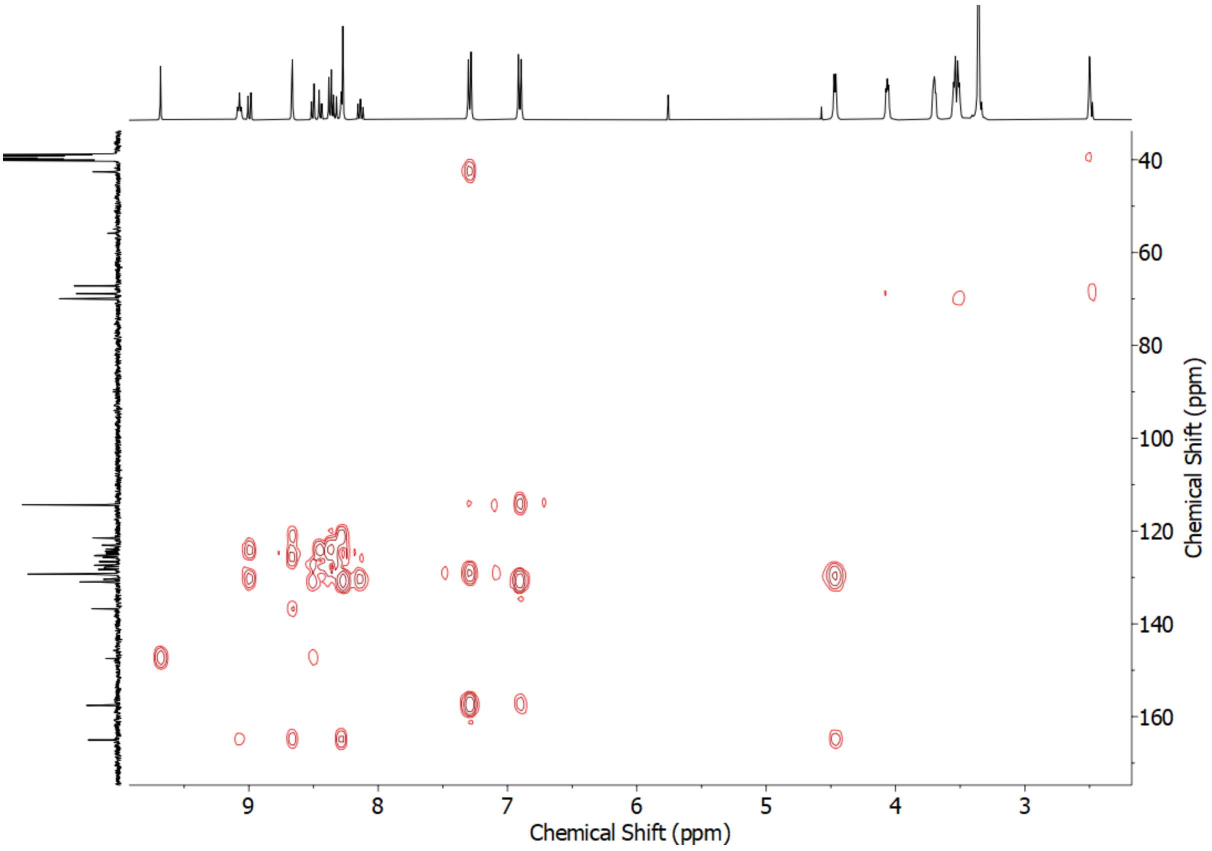

Figure S36 HMBC NMR (d_6-DMSO) of **3^{Py}**

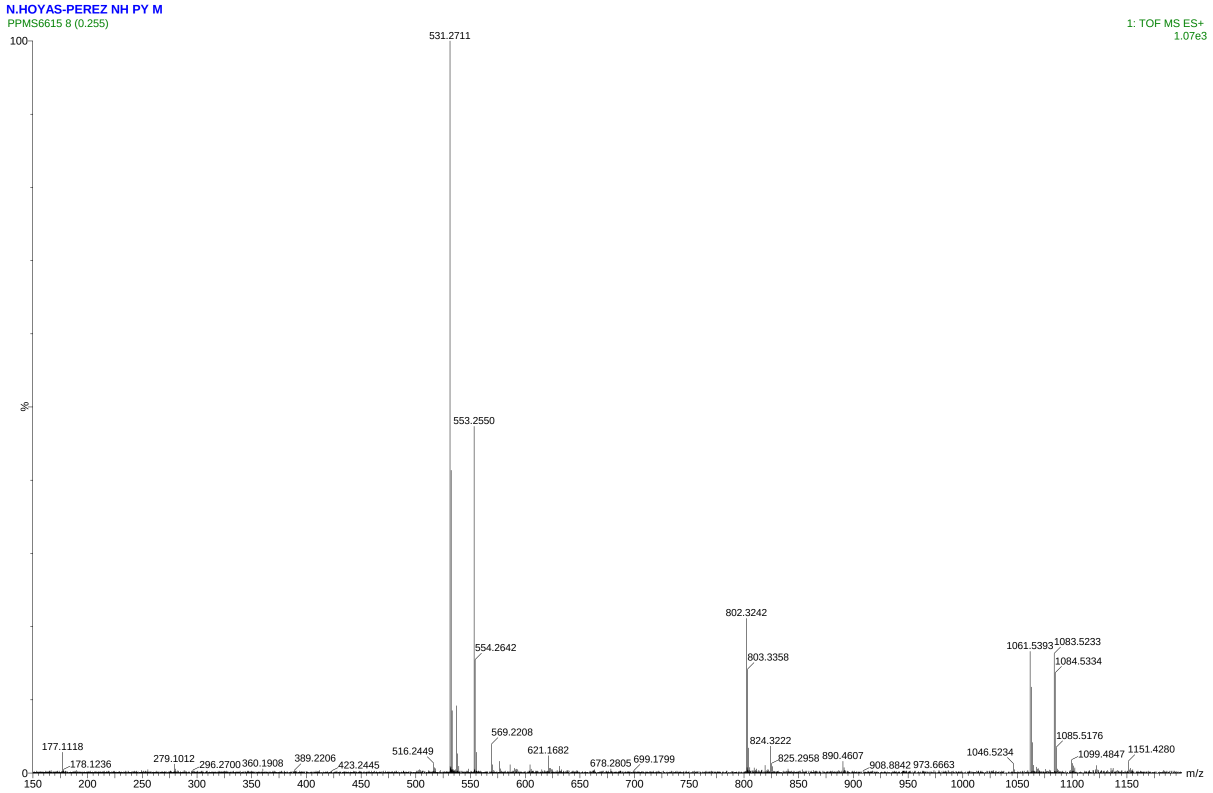

Figure S37 HR-ESI-MS of $\mathbf{3}^{\mathbf{Py}}$

Synthesis of 4^{Py}

Prepared according to general procedure with $\mathbf{4^{N3}}$ (38.8 mg, 0.025 mmol, 1 eq.), 1-ethynylpyrene (16.8 mg, 0.075 mmol, 3 eq.), $[Cu(CH_3CN)_4](PF_6)$ (13.2 mg, 0.025 mmol, 1 eq.), TBTA (9.4 mg, 0.025 mmol, 1 eq.), and purified by column chromatography on silica (3:7 acetone/CH_2Cl_2) to give $\mathbf{4^{Py}}$ as a yellow/orange solid (25.3 mg, 63%).

^{1}H NMR (500 MHz, $CDCl_3$) δ: 8.76 (d, *J* = 9.2 Hz, 2H, H_r/H_s), 8.67 (br. s, 2H, H_b), 8.61 (s, 2H, H_j), 8.48 (br. s, 4H, H_a), 8.37 (d, *J* = 7.9 Hz, 2H, H_k), 8.28 (d, *J* = 7.9 Hz, 2H, H_l), 8.24 (m, 4H, H_o, H_q), 8.19 (d, *J* = 9.3 Hz, 2H, H_r/H_s), 8.15-8.10 (m, 4H, H_m, H_n), 8.05 (app. t, *J* = 7.6 Hz, 2H, H_p), 7.89 (br. s, 4H, H_{NH}), 7.08 (d, *J* = 7.8 Hz, 8H, H_d), 6.59 (d, *J* = 7.8 Hz, 8H, H_e), 4.46 (br. s, 8H, H_c), 3.74 (br. s, 8H, H_f), 3.57 (br. s, 8H, H_g), 3.47 (br. s, 8H, H_h), 3.39 (br. s, 8H, H_i).

^{13}C NMR (126 MHz, $CDCl_3$) δ: 165.1, 157.8, 148.6, 137.0, 136.0, 131.8, 131.5, 130.9, 129.9, 128.8, 128.6, 128.3, 127.4, 127.0, 126.3, 125.8, 125.6, 125.2, 125.2, 124.9, 124.8, 124.4, 124.1, 122.7, 121.2, 114.1, 70.5, 70.4, 69.6, 67.0, 44.1 (1 signal missing due to peak overlap).

HR-ESI-MS *m/z* = 1606.6439 $[M+H]^+$ cal. 1606.6778.

M.p. 163±1 °C.

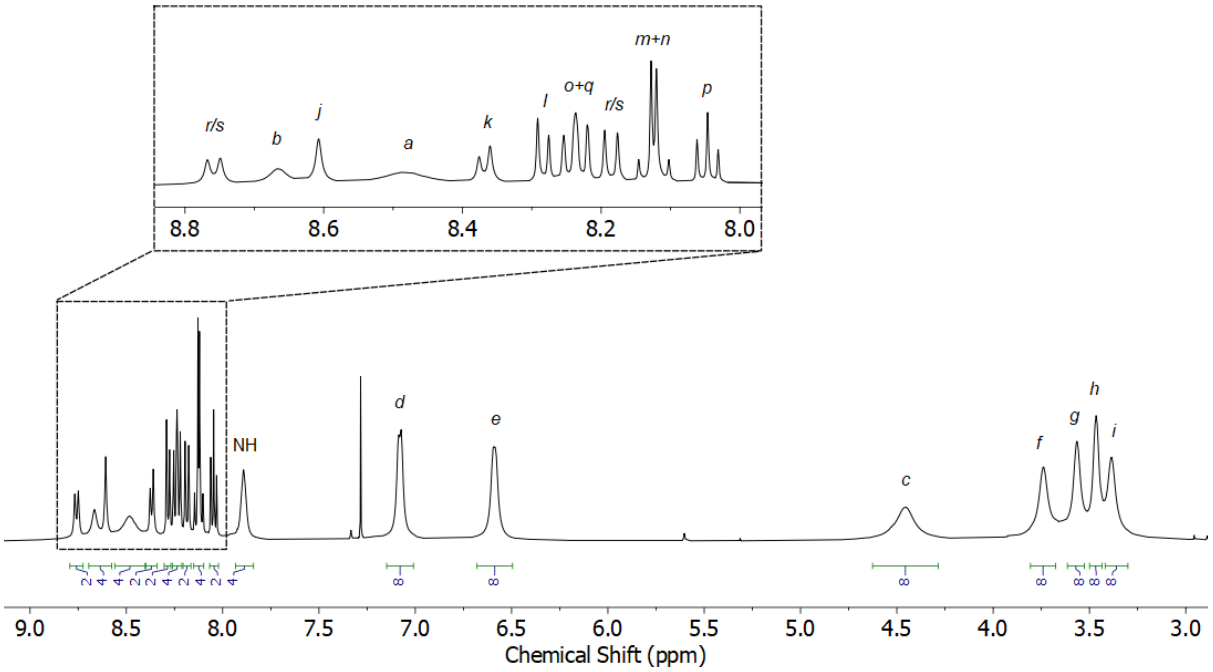

Figure S38 ^{1}H NMR ($CDCl_3$, 500 MHz) of $\mathbf{4^{Py}}$

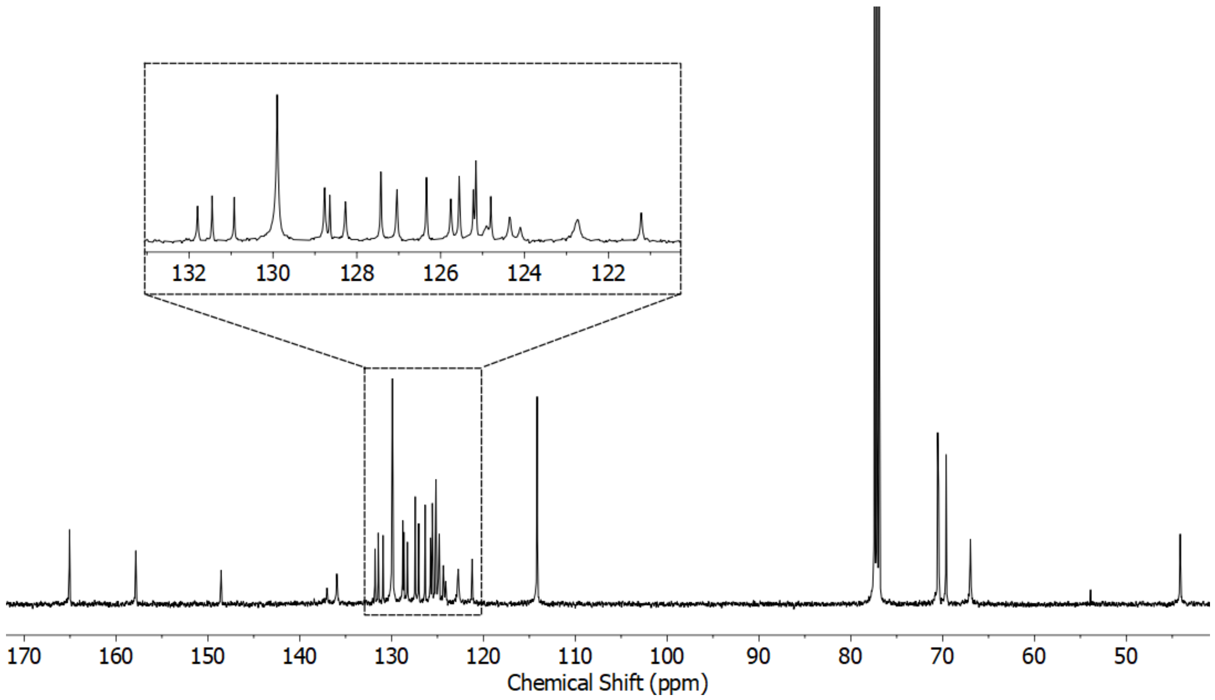

Figure S39 ^{13}C NMR ($CDCl_3$, 126 MHz) of $\mathbf{4^{Py}}$

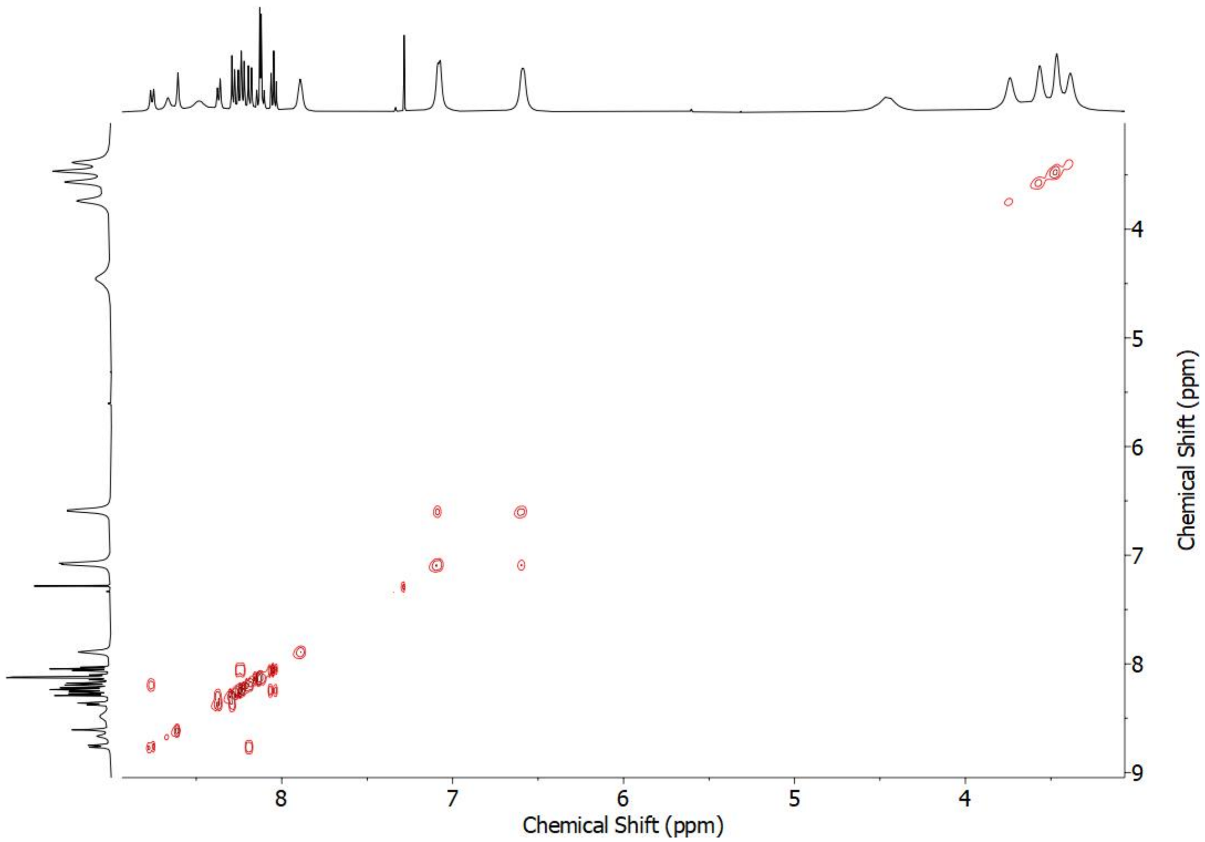

Figure S40 COSY ($CDCl_3$) of $\mathbf{4^{Py}}$

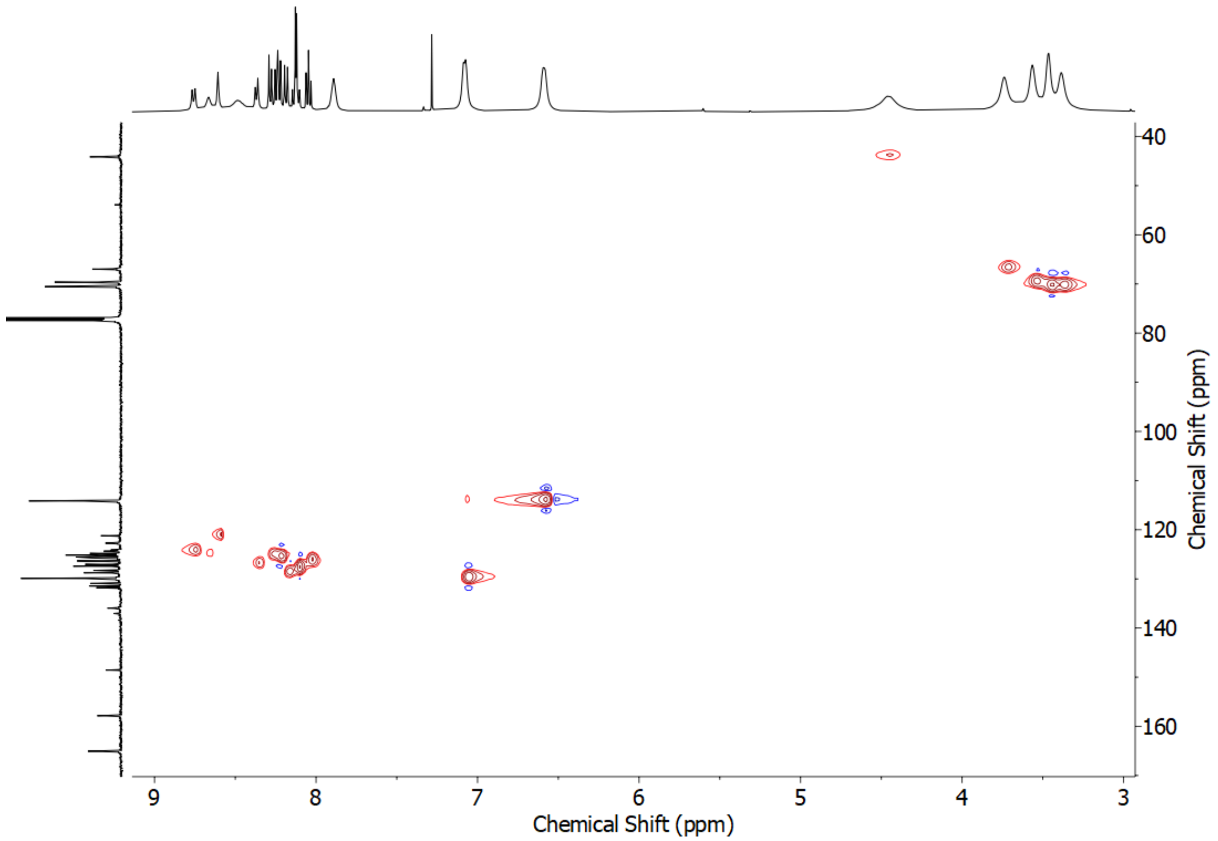

Figure S41 HSQC ($CDCl_3$) of **4**^{Py}

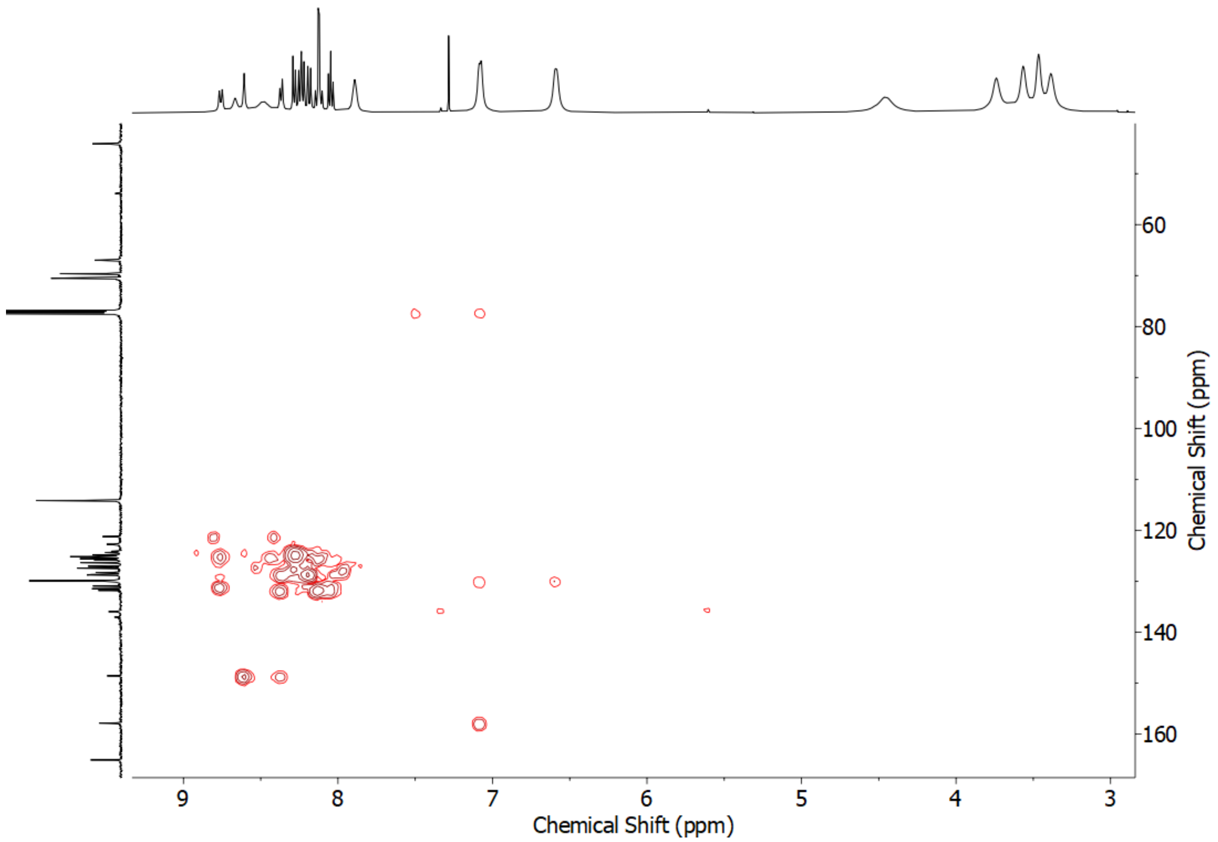

Figure S42 HMBC ($CDCl_3$) of **4**^{Py}

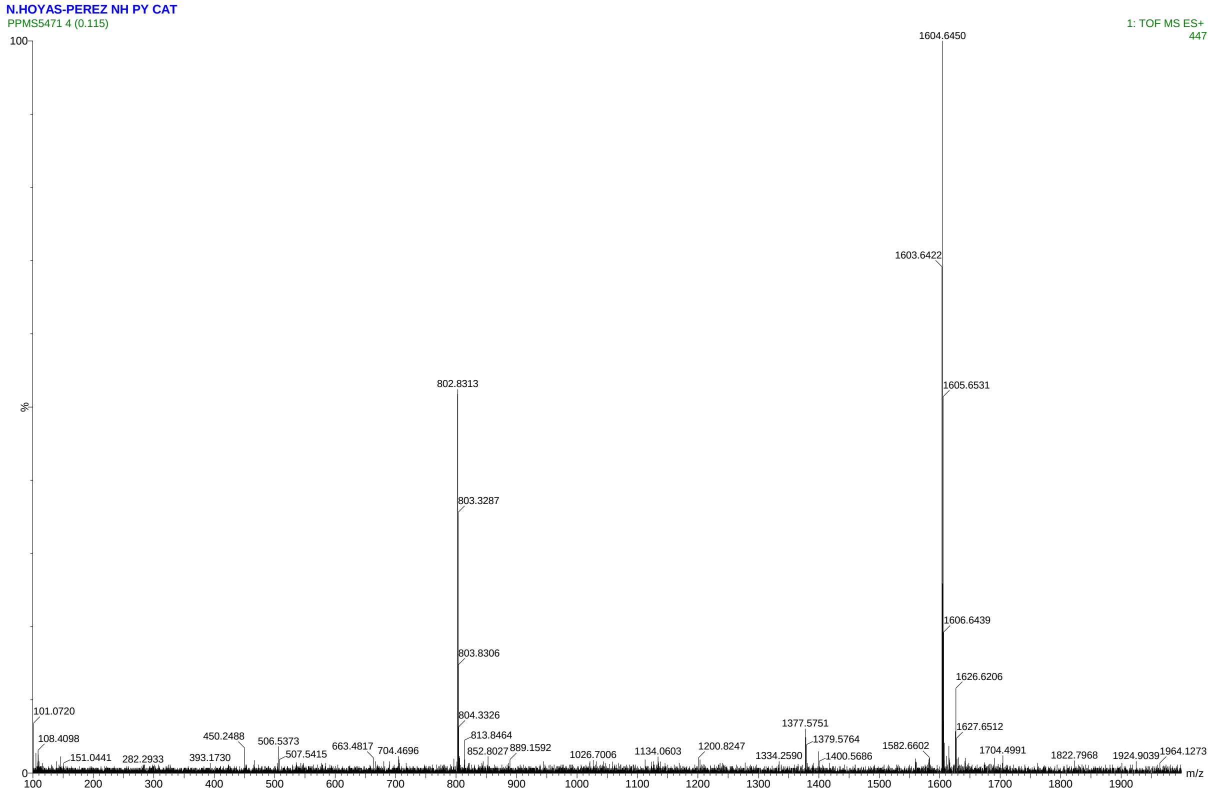

Figure S43 HR-ESI-MS of **4**^{Py}

Synthesis of 3^{An}

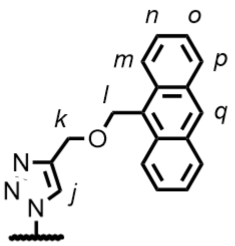

Prepared according to general procedure with $\mathbf{3^{N3}}$ (14.3 mg, 0.025 mmol, 1 eq.), **S1** (9.1 mg, 0.037 mmol, 1.5 eq.), $[Cu(CH_3CN)_4](PF_6)$ (6.6 mg, 0.012 mmol, 0.5 eq.), TBTA (4.7 mg, 0.012 mmol, 0.5 eq.), and purified by column chromatography on silica (1:4 acetone/CH_2Cl_2) to give $\mathbf{3^{An}}$ as a yellow solid (14.5 mg, 71%).

^{1}H NMR (400 MHz, d_6-DMSO) δ: 9.03-9.00 (m, 3H, H_j, H_{NH}), 8.65 (s, 1H, H_q), 8.45-8.41 (m, 4H, H_a,H_m/H_p), 8.22 (s, 1H, H_b), 8.12 (d, J = 7.7 Hz, 2H, H_m/H_p), 7.62-7.57 (m, 2H, H_n/H_o), 7.56-7.52 (m, 2H H_n/H_o), 7.25 (d, J = 8.5 Hz, 4H, H_d), 6.88 (d, J = 8.6 Hz, 4H, H_e), 5.60 (s, 2H, H_l), 4.94 (s, 2H, H_k), 4.42 (d, J = 5.5 Hz, 4H, H_c), 4.05 (m, 4H, H_f), 3.68 (m, 4H, H_g), 3.54-3.49 (m, 8H, H_h, H_i).

^{13}C NMR (101 MHz, d_6-DMSO) δ: 164.9, 157.5, 145.6, 136.8, 136.6, 131.0, 130.9, 130.5, 129.2, 128.8, 128.1, 126.4, 125.5, 125.2, 124.5, 122.6, 121.2, 114.3, 79.2, 69.9, 69.9, 68.8, 67.1, 63.8, 63.2, 42.5.

HR-ESI-MS *m/z* = 822.3481 $[M+H]^+$calc. 822.3503.

M.p. 118±1 °C.

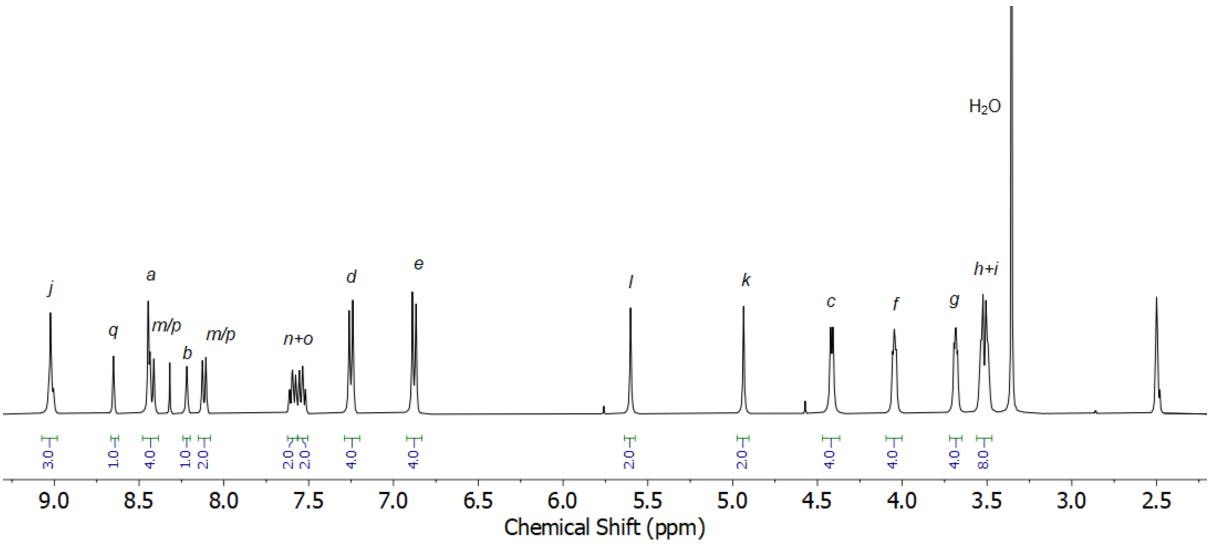

Figure S44 ^{1}H NMR (d_6-DMSO, 400 MHz) of $\mathbf{3^{An}}$

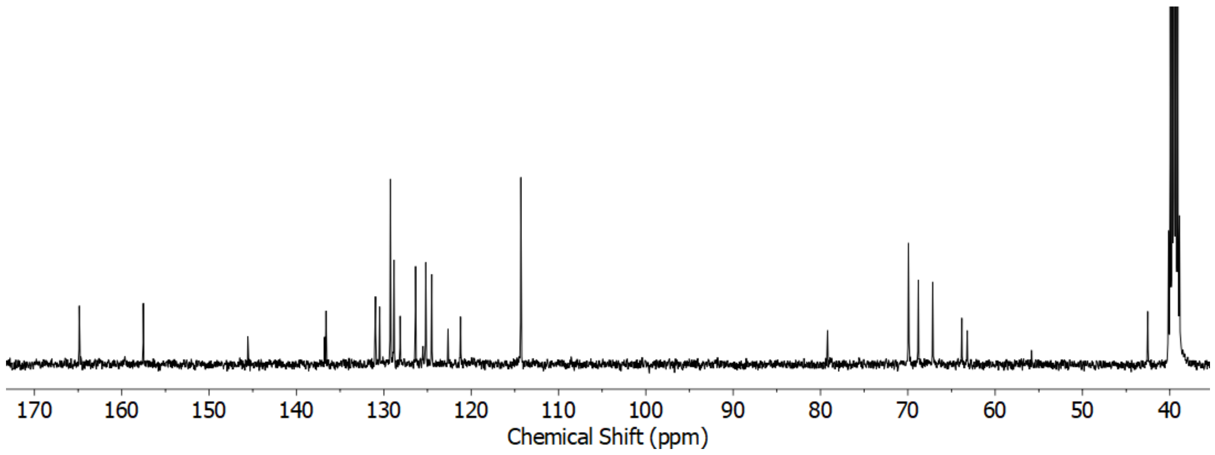

Figure S45 ^{13}C NMR (d_6-DMSO, 101 MHz) of $\mathbf{3^{An}}$

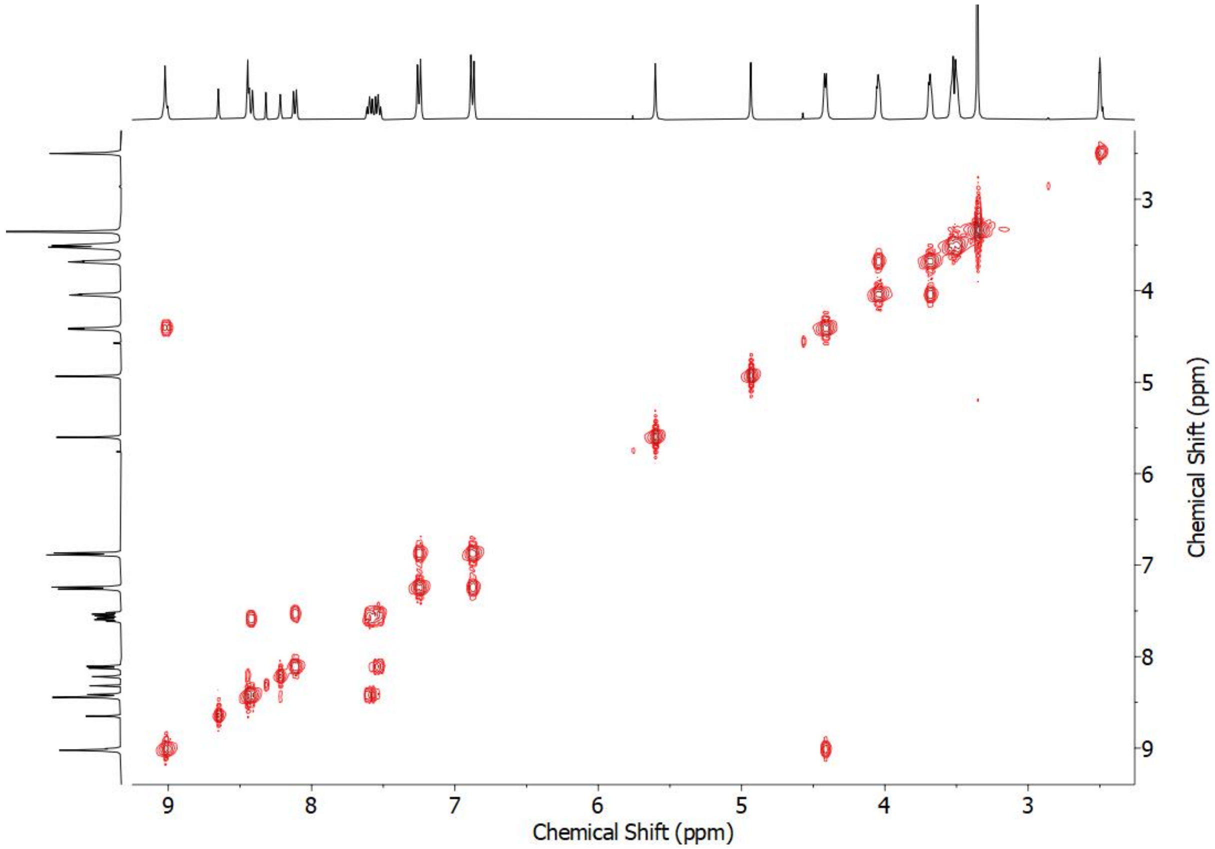

Figure S46 COSY (d_6-DMSO) of $\mathbf{3^{An}}$

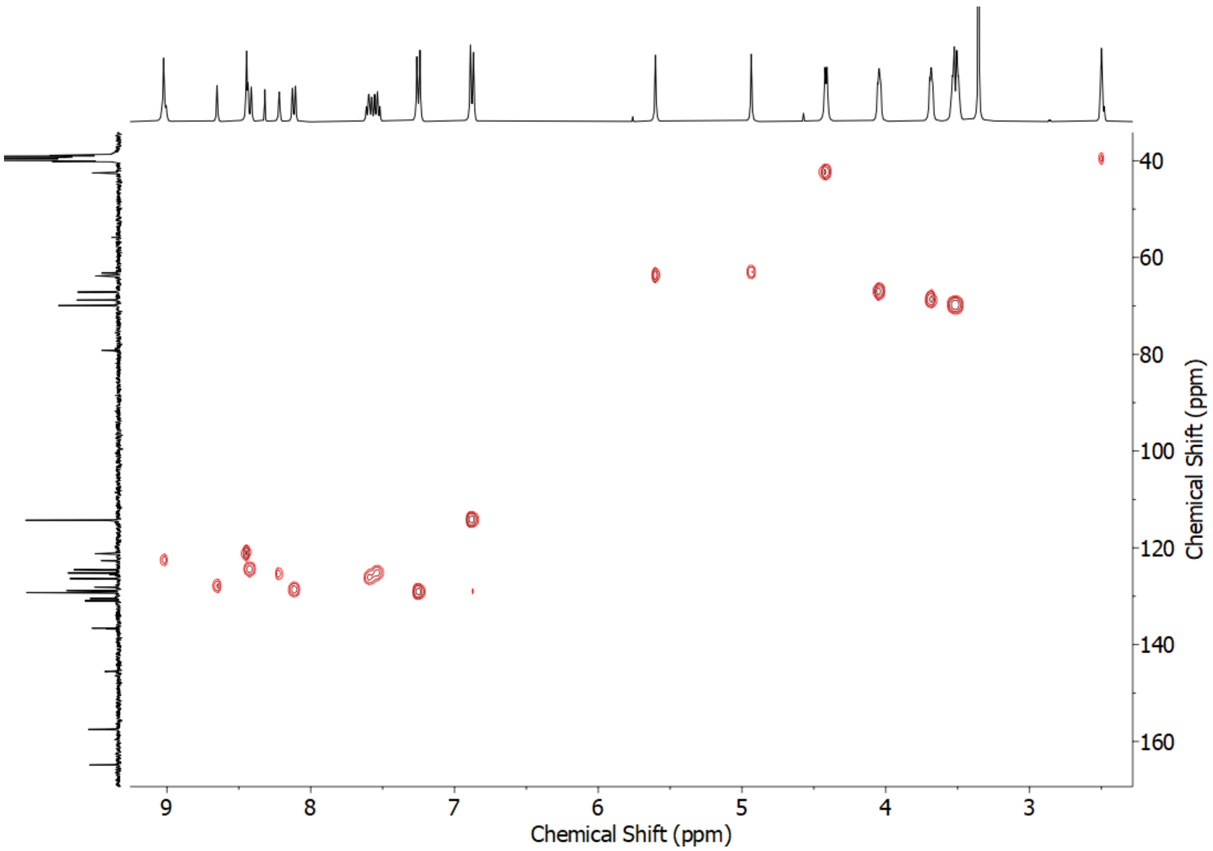

Figure S47 HSQC (d_6-DMSO) of **3**^{An}

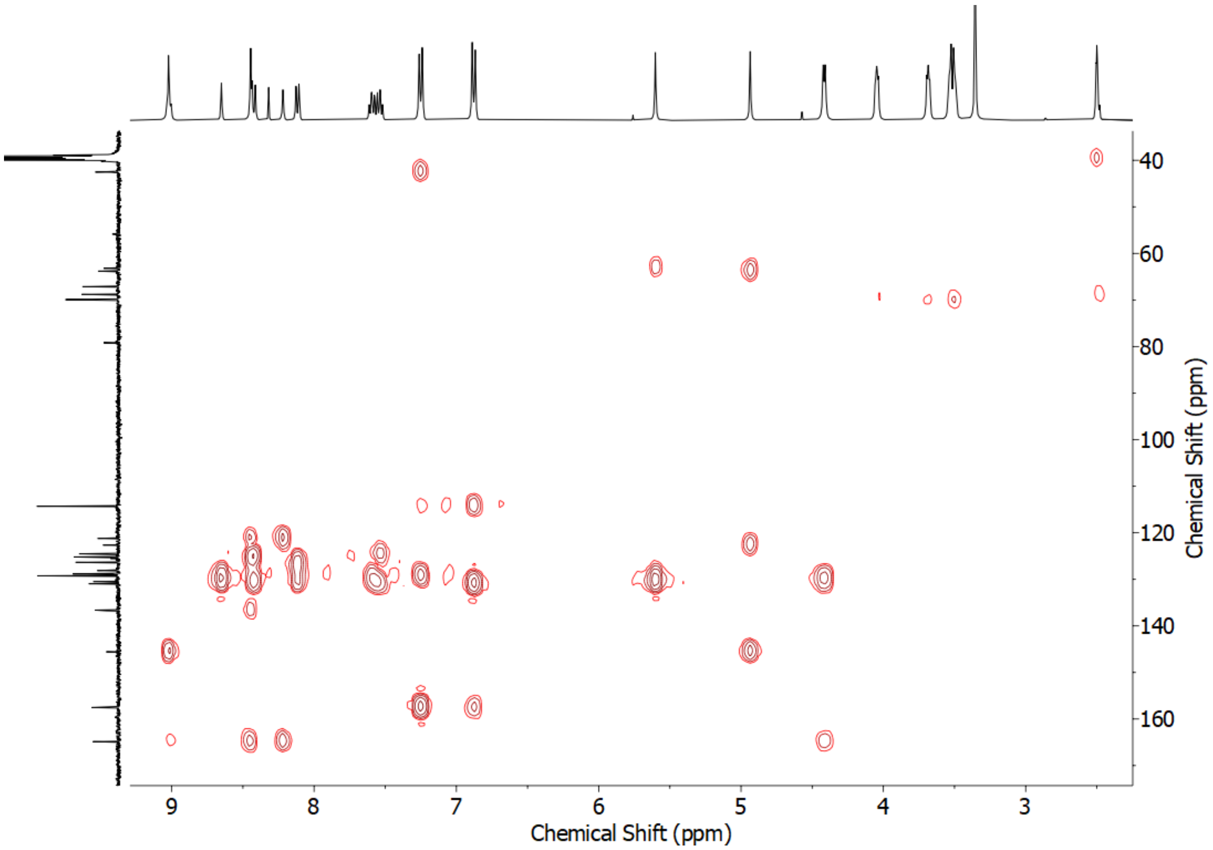

Figure S48 HMBC (d_6-DMSO) of **3**^{An}

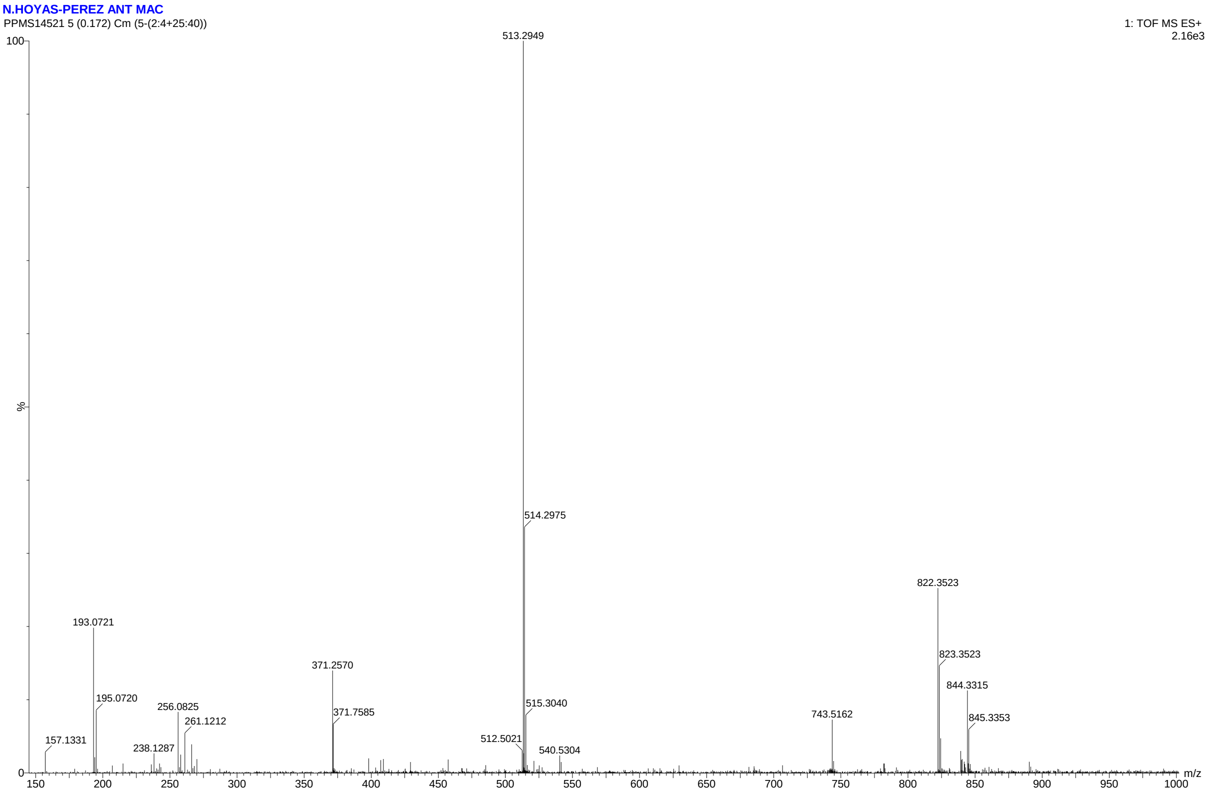

Figure S49 HR-ESI-MS of $\mathbf{3^{An}}$

Synthesis of 4^{An}

Prepared according to general procedure with $\mathbf{4^{N3}}$ (38.8 mg, 0.025 mmol, 1 eq), **S1** (18.4 mg, 0.075 mmol, 3 eq.), $[Cu(CH_3CN)_4](PF_6)$ (13.2 mg, 0.025 mmol, 1 eq.), TBTA (9.4 mg, 0.025 mmol, 1 eq.), and purified by column chromatography on silica (3:7 acetone/CH_2Cl_2) to give $\mathbf{4^{An}}$ as a yellow solid (23.2 mg, 56%).

^{1}H NMR (400 MHz, $CDCl_3$) δ: 8.48 (s, 2H, H_q), 8.42-8.40 (m, 6H, H_b/H_j, H_m/H_p), 8.17 (br. s, 4H, H_a), 8.02-8.00 (m, 6H, H_b/H_j, H_m/H_p), 7.76 (br. s, 4H, H_{NH}), 7.55 (ddd, J = 8.9, 6.5, 1.4 Hz, 4H, H_n/H_o), 7.47 (ddd, J = 8.5, 6.5, 1.0 Hz, 4H, H_n/H_o), 6.92 (d, J = 8.0 Hz, 8H, H_d), 6.45 (m, 8H, H_e), 5.70 (s, 4H, H_l), 4.91 (s, 4H, H_k), 4.23 (br. s, 8H, H_c), 3.64 (s, 8H, H_f), 3.47 (s, 8H, H_g), 3.37 (s, 8H, H_h), 3.29 (s, 8H, H_i).

^{13}C NMR (101 MHz, $CDCl_3$) δ: 164.9, 157.7, 146.6, 136.9, 135.9, 131.5, 131.2, 130.0, 129.8, 129.2, 128.8, 128.1, 126.6, 125.2, 124.9, 124.2, 122.7, 121.5, 114.0, 70.5, 70.3, 69.5, 66.9, 65.1, 63.7, 43.8.

HR-ESI-MS *m/z* = 1644.7061 $[M+H]^+$ calc. 1644.7006.

M.p. 150±1 °C.

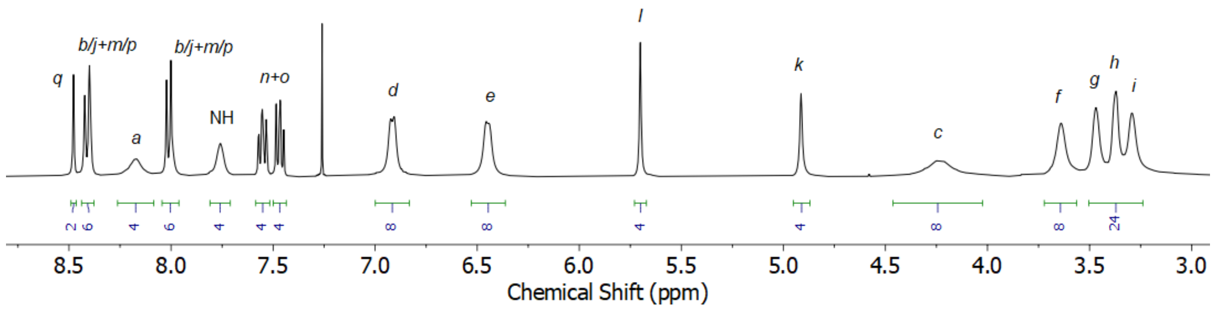

Figure S50 ^{1}H NMR ($CDCl_3$, 400 MHz) of $\mathbf{4^{An}}$

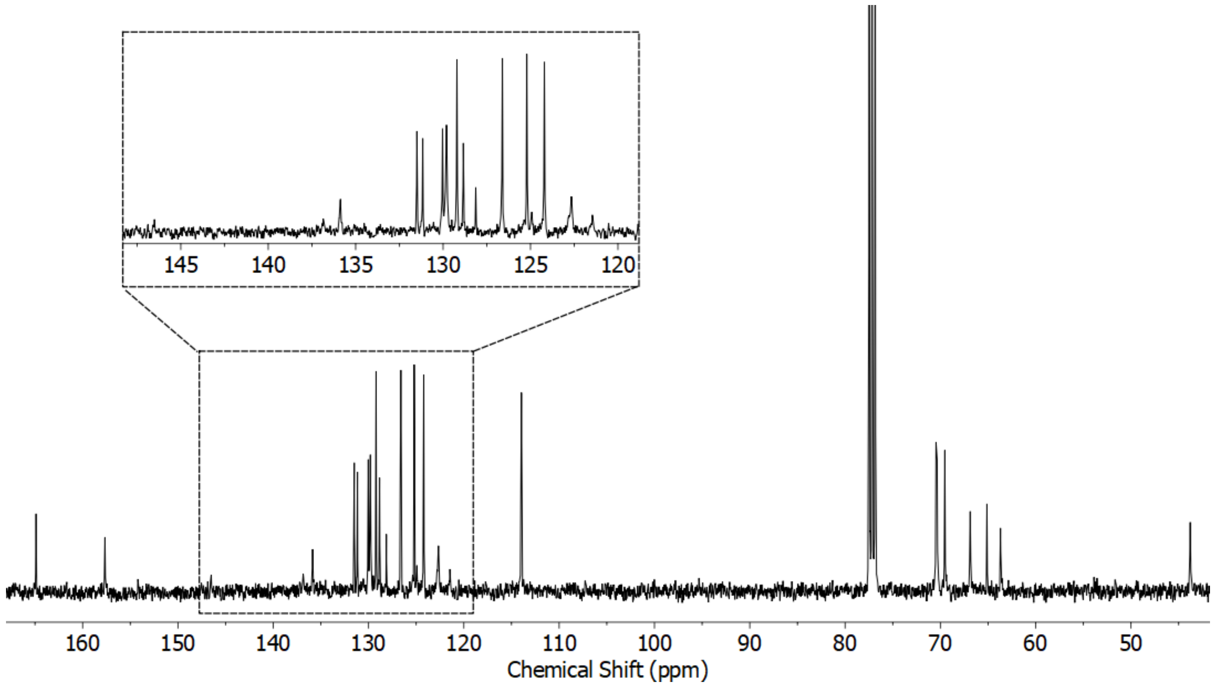

Figure S51 ^{13}C NMR ($CDCl_3$, 400 MHz) of $\mathbf{4^{An}}$

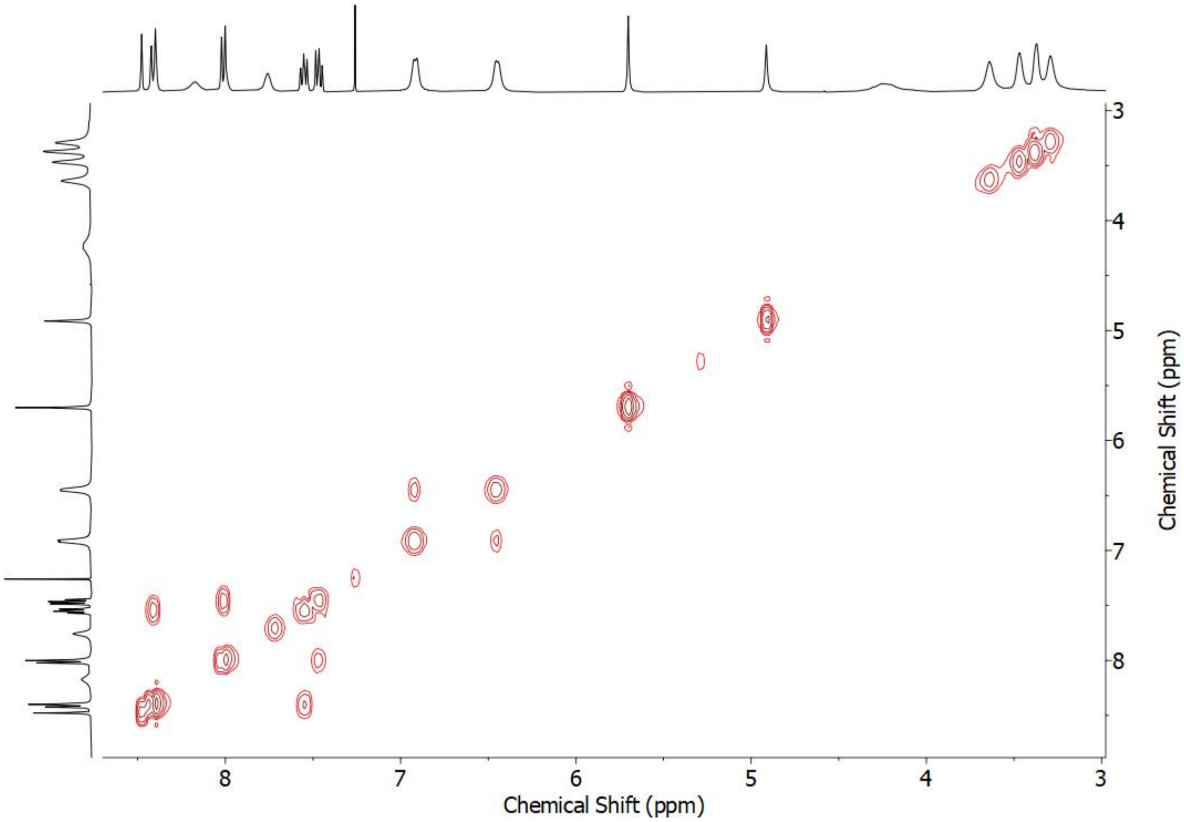

Figure S 52 COSY ($CDCl_3$) of **4^{An}**

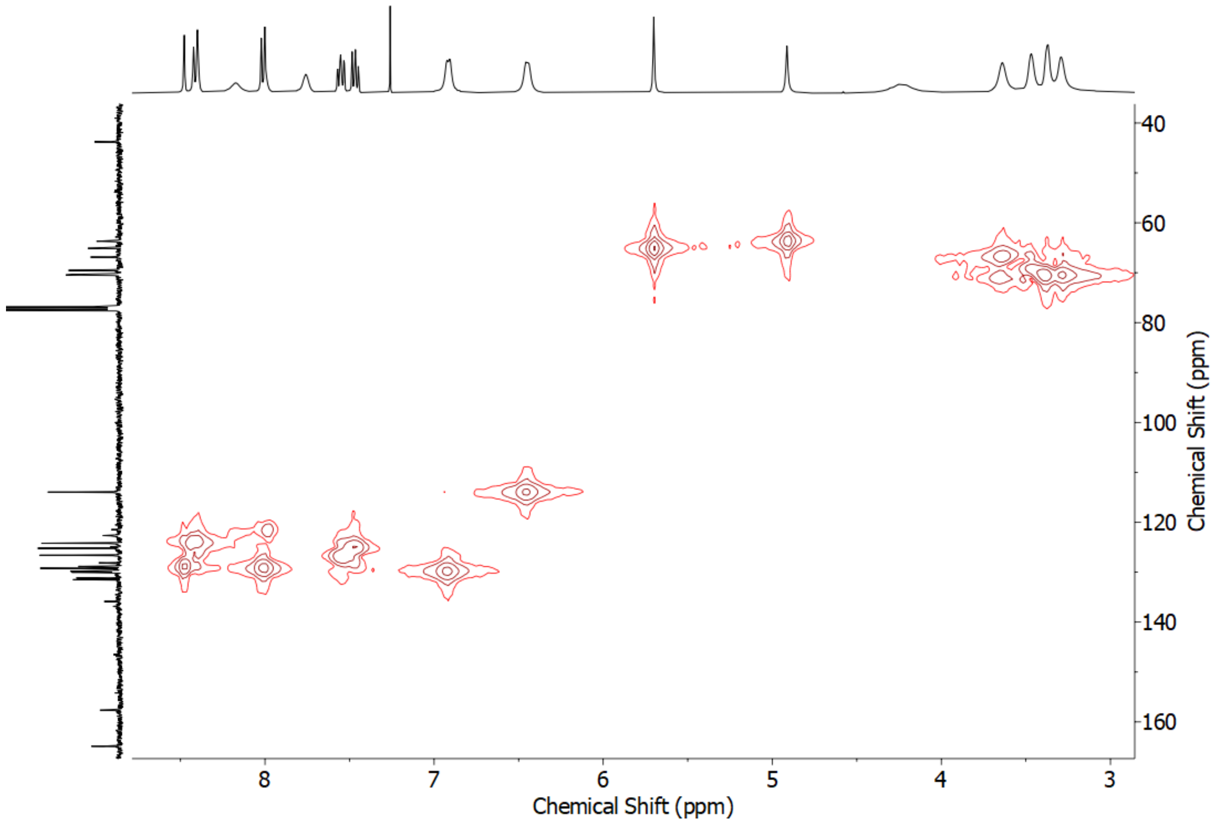

Figure S53 HSQC ($CDCl_3$) of **4^{An}**

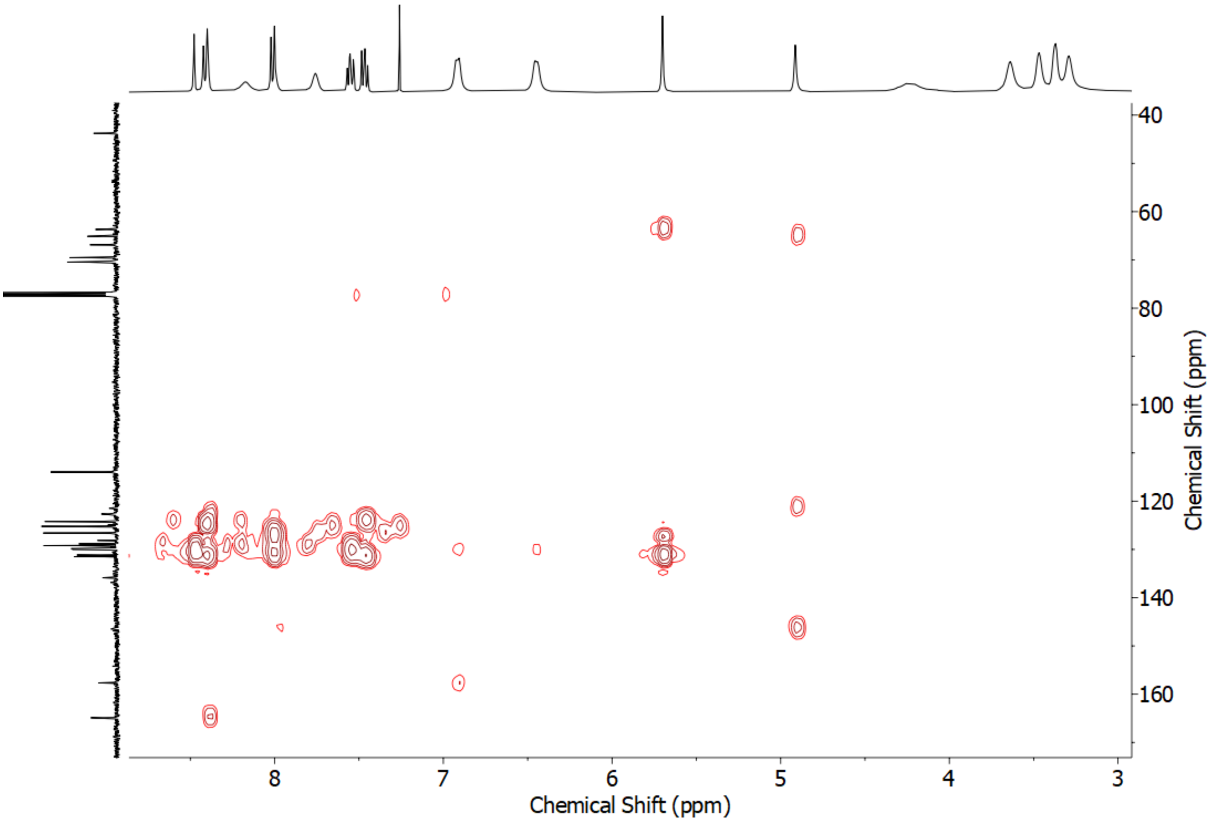

Figure S54 HMBC ($CDCl_3$) of $\mathbf{4^{An}}$

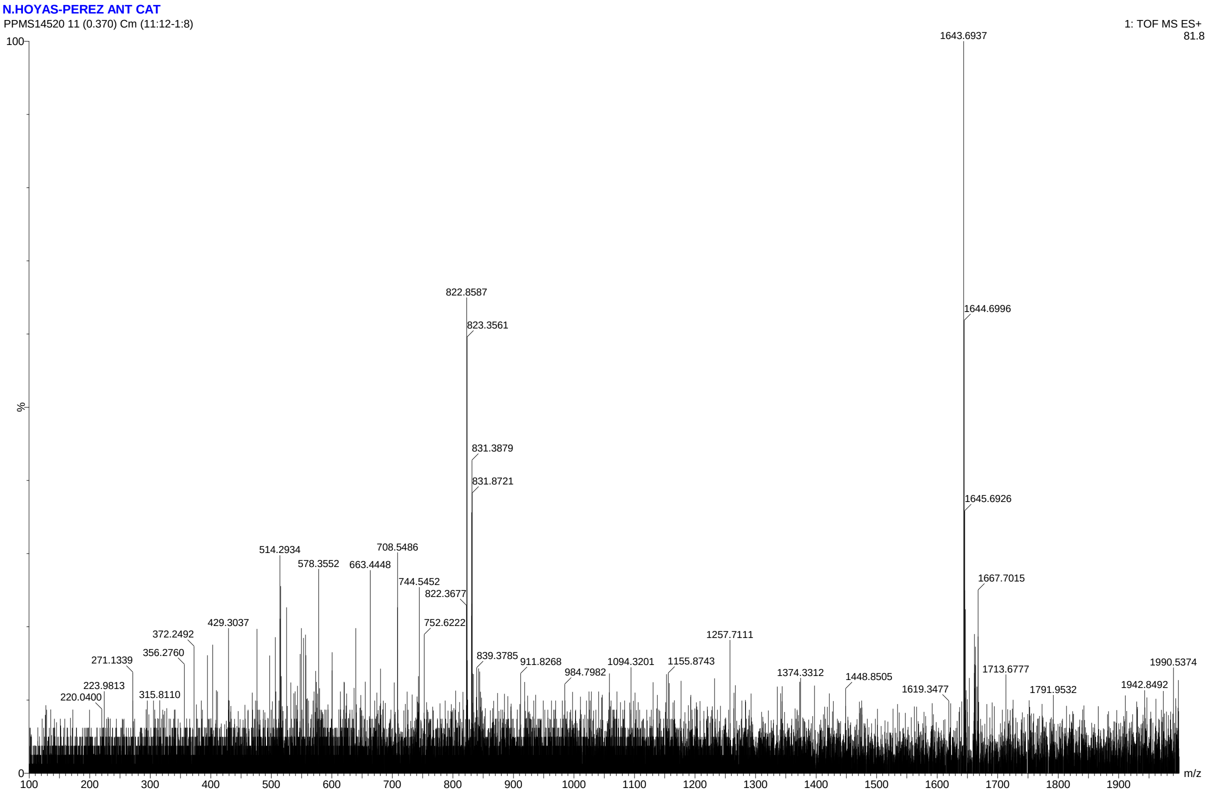

Figure S55 HR-ESI-MS of $\mathbf{4^{An}}$

Synthesis of 3^{Fc}

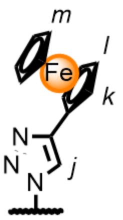

Prepared according to general procedure with **3^{N3}** (14.3 mg, 0.025 mmol, 1 eq.), ethynylferrocene (7.9 mg, 0.037 mmol, 1.5 eq.), [Cu(CH_3CN)$_4$](PF_6) (6.6 mg, 0.012 mmol, 0.5 eq.), TBTA (4.7 mg, 0.012 mmol, 0.5 eq.), and purified by column chromatography on silica (1:4 acetone/CH_2Cl_2) to give **3^{Fc}** as an orange solid (14.9 mg, 83%).

^{1}H NMR (500 MHz, *d*$_6$-DMSO) δ: 9.13 (s, 1H, H$_j$), 9.02 (t, *J* = 5.7 Hz, 2H, H$_{NH}$), 8.48 (d, *J* = 1.5 Hz, 2H, H$_a$), 8.22 (t, *J* = 1.5 Hz, 1H, H$_b$), 7.27 (d, *J* = 8.7 Hz, 4H, H$_d$), 6.89 (d, *J* = 8.7 Hz, 4H, H$_e$), 4.85 (t, *J* = 1.8 Hz, 2H, H$_k$/H$_l$), 4.45 (d, *J* = 5.6 Hz, 4H, H$_c$), 4.38 (t, *J* = 1.9 Hz, 2H, H$_k$/H$_l$), 4.11 (s, 5H, H$_m$), 4.06 (m, 4H, H$_f$), 3.70 (m, 4H, H$_g$), 3.56-3.50 (m, 8H, H$_h$, H$_i$).

^{13}C NMR (126 MHz, *d*$_6$-DMSO) δ: 165.0, 157.6, 147.1, 136.8, 136.6, 131.0, 129.3, 125.2, 120.8, 118.7, 114.3, 75.0, 69.9, 69.9, 69.4, 68.8, 68.6, 67.2, 66.5, 42.5.

HR-ESI-MS *m/z* = 786.2577 [M+H]$^+$ calc. 786.2585.

M.p. 175±1 °C.

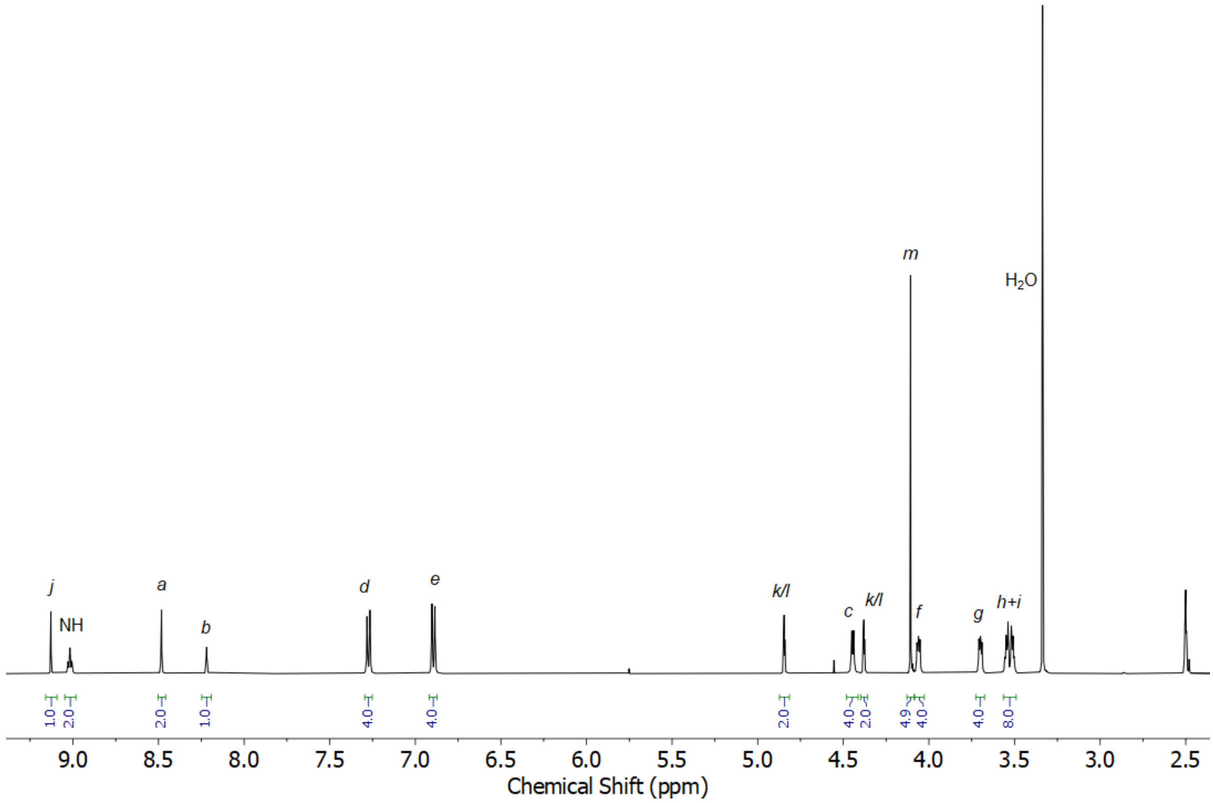

Figure S56 ^{1}H NMR (*d*$_6$-DMSO, 500 MHz) of **3^{Fc}**

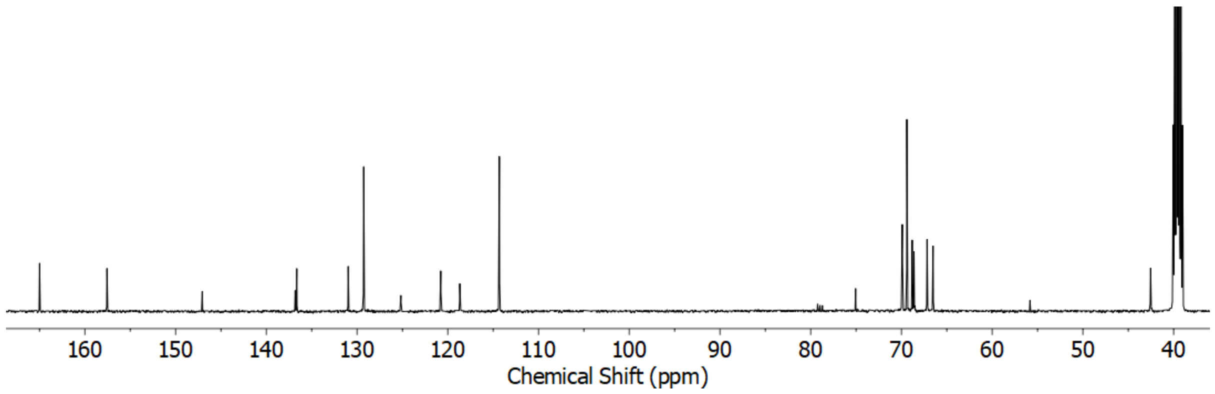

Figure S57 ^{13}C NMR (d_6-DMSO, 126 MHz) of $\mathbf{3^{Fc}}$

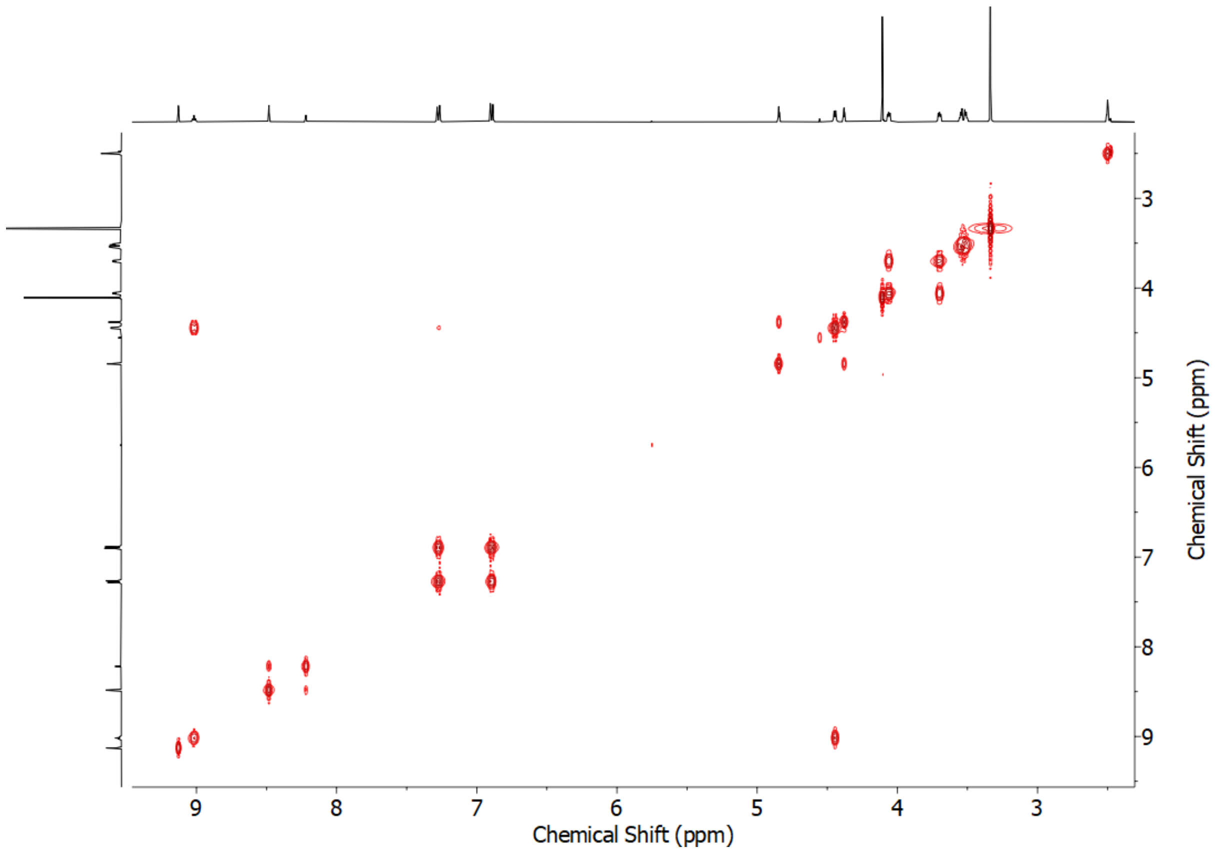

Figure S58 COSY (d_6-DMSO) of $\mathbf{3^{Fc}}$

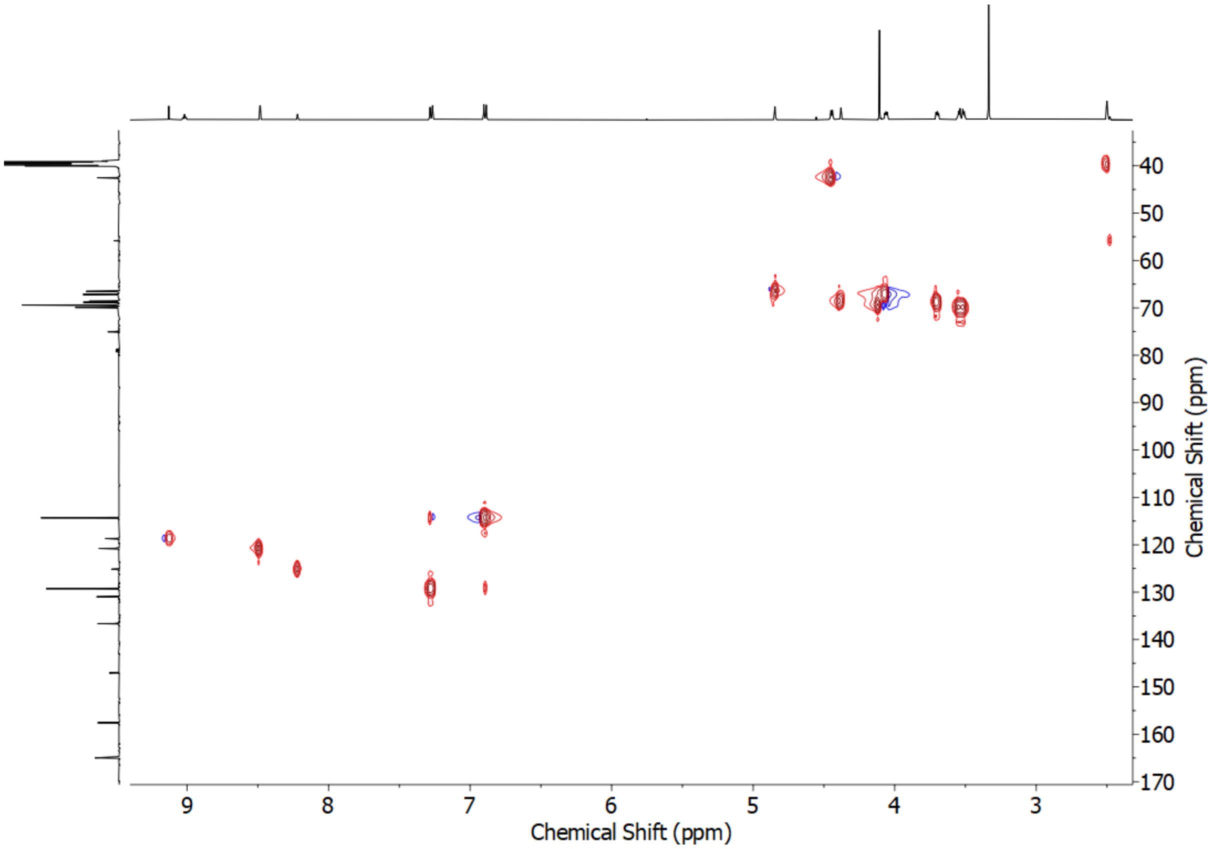

Figure S59 HMBC (d_6-DMSO) of **3**^{Fc}

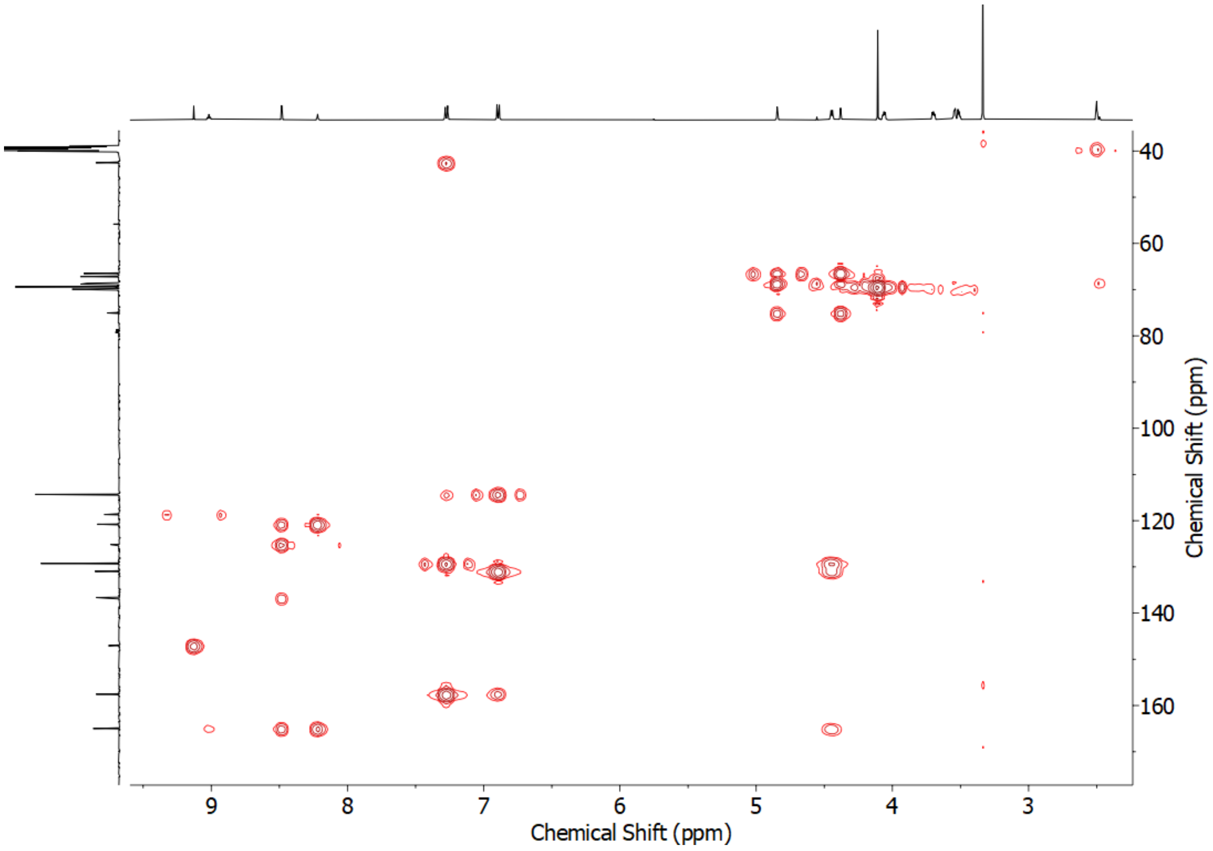

Figure S60 HSQC (d_6-DMSO) of **3**^{Fc}

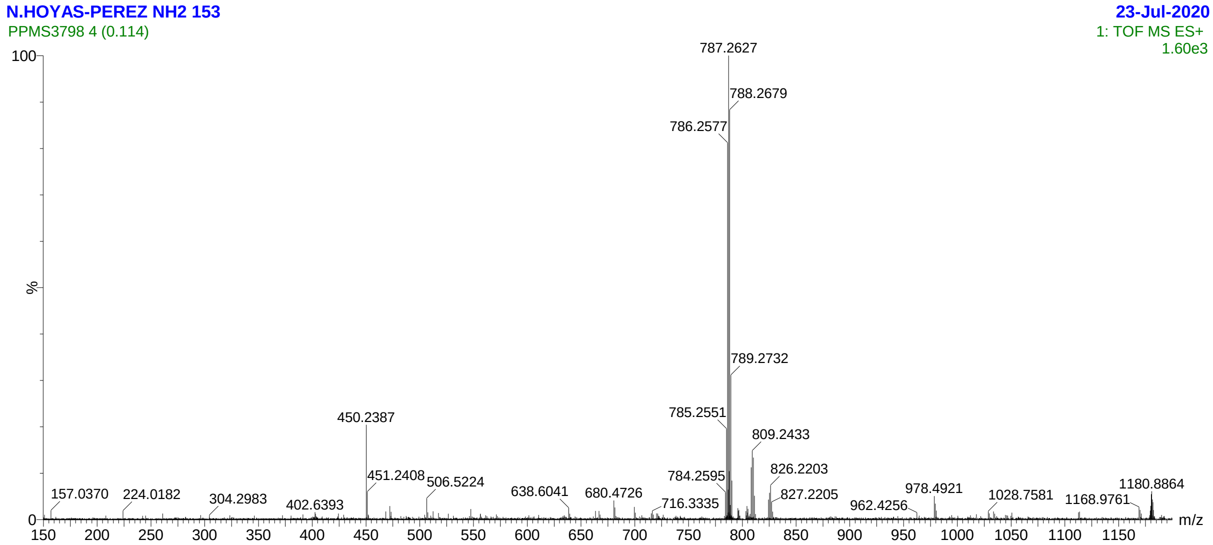

Figure S61 HR-ESI-MS of **3**$^{\mathbf{Fc}}$

Synthesis of 4^{Fc}

Prepared according to general procedure with $\mathbf{4^{N3}}$ (38.8 mg, 0.025 mmol, 1 eq.), ethynylferrocene (15.8 mg, 0.075 mmol, 3 eq.), $[Cu(CH_3CN)_4](PF_6)$ (13.2 mg, 0.025 mmol, 1 eq.), TBTA (9.4 mg, 0.025 mmol, 1 eq.), and purified by column chromatography on silica (3:7 acetone/CH_2Cl_2) to give $\mathbf{4^{Fc}}$ as an orange/brown solid (31.4 mg, 80%).

^{1}H NMR (500 MHz, $CDCl_3$) δ: 8.56 (s, 2H, H_b), 8.32 (br. s, 4H, H_a), 8.02 (s, 2H, H_j), 7.79 (br. s, 4H, H_{NH}), 7.03 (br. m, 8H, H_d), 6.55 (br. m, 8H, H_e), 4.76 (t, J = 1.9 Hz, 4H, H_k/H_l), 4.45 (br. m, 4H, H_c), 4.37 (t, J = 1.8 Hz, 4H, H_k/H_l), 4.12 (s, 10H, H_m), 3.68 (br. m, 8H, H_f), 3.53 (br. m, 8H, H_g), 3.47 (br. m, 8H, H_h), 3.36 (br. m, 8H, H_i).

^{13}C NMR (126 MHz, $CDCl_3$) δ: 165.1, 157.8, 148.3, 137.0, 135.8, 130.0, 129.8, 129.0, 124.6, 122.6, 117.0, 114.1, 74.3, 70.6, 70.4, 69.8, 69.6, 69.3, 66.9 (×2), 44.0.

HR-ESI-MS *m/z* = 1571.5138 $[M+H]^+$ calc. 1571.5097.

M.p. 148±1 °C.

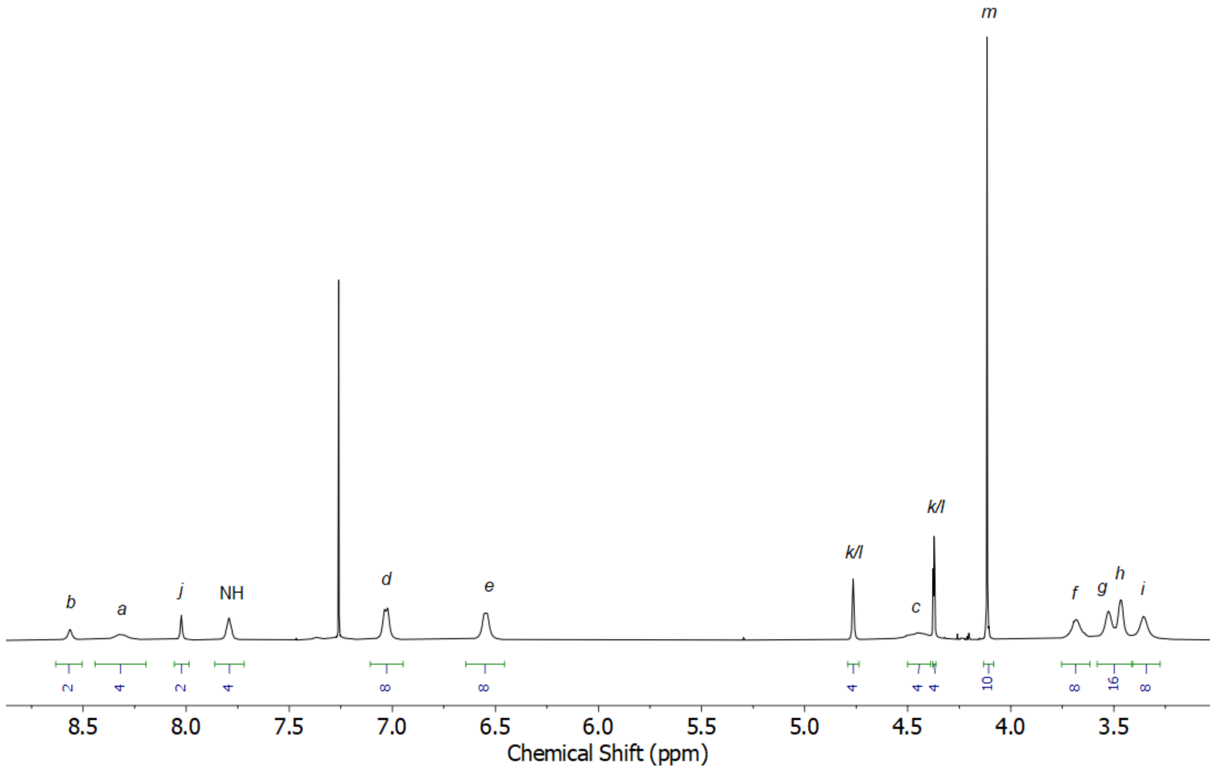

Figure S62 ^{1}H NMR ($CDCl_3$, 500 MHz) of $\mathbf{4^{Fc}}$

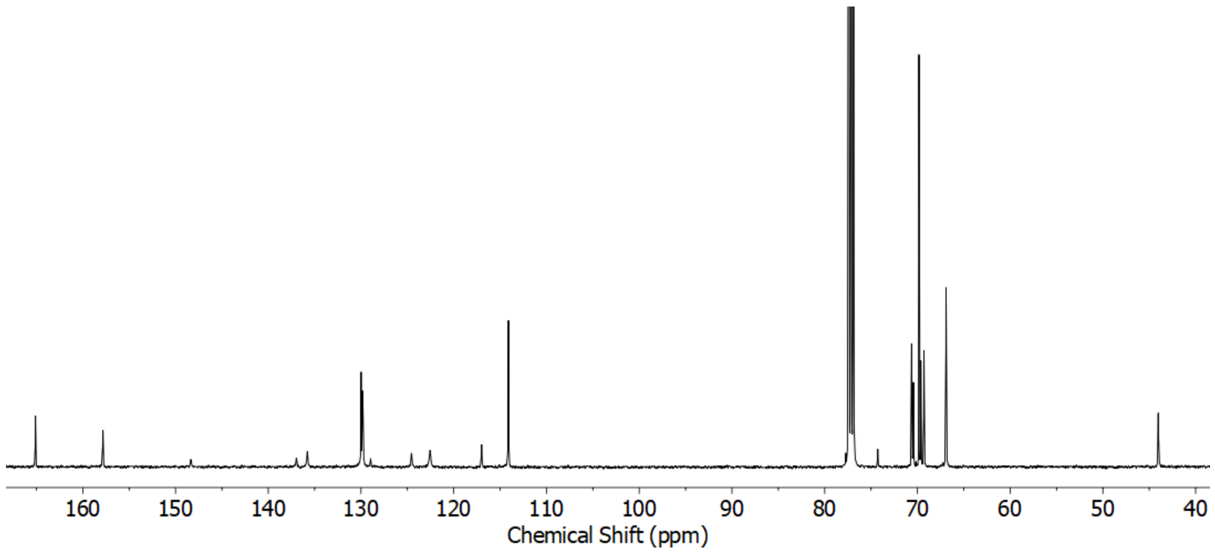

Figure S63 ^{13}C NMR ($CDCl_3$, 500 MHz) of $\mathbf{4^{Fc}}$

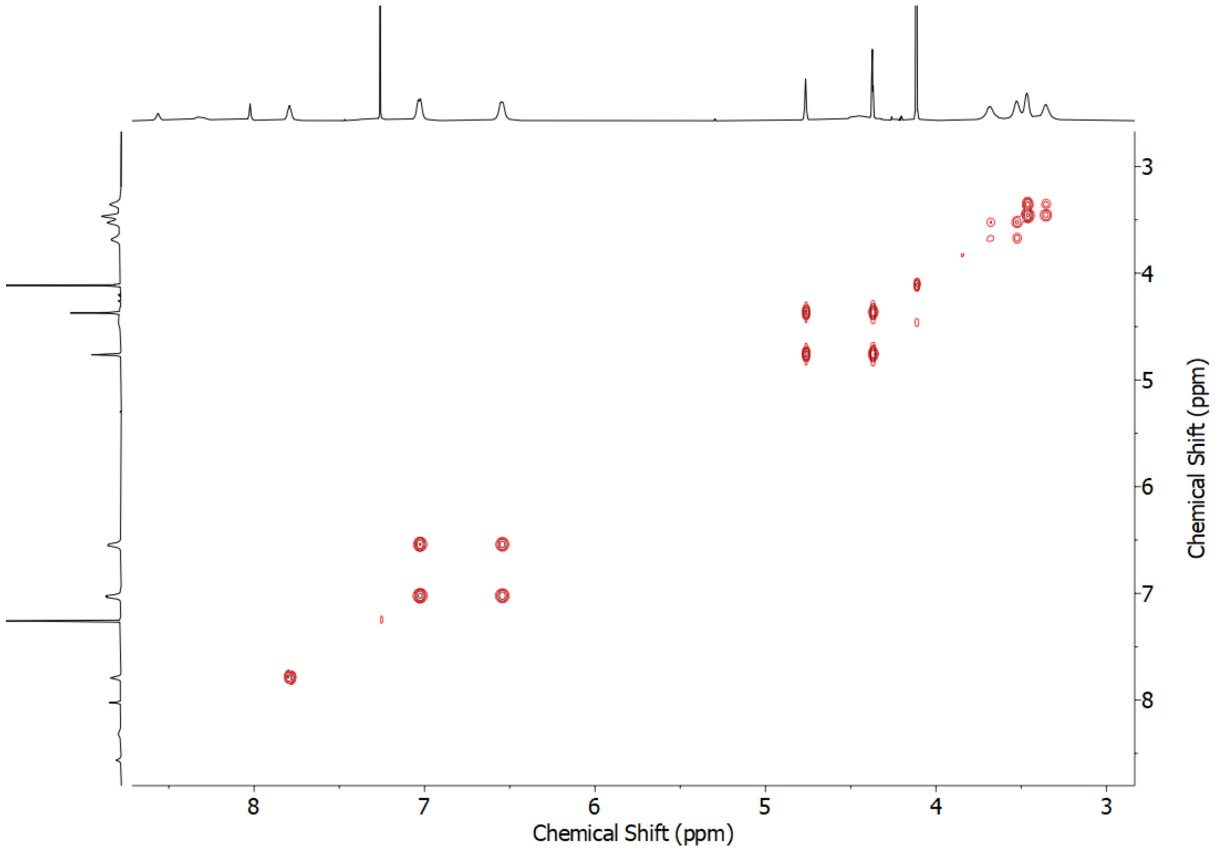

Figure S64 COSY ($CDCl_3$) of $\mathbf{4^{Fc}}$

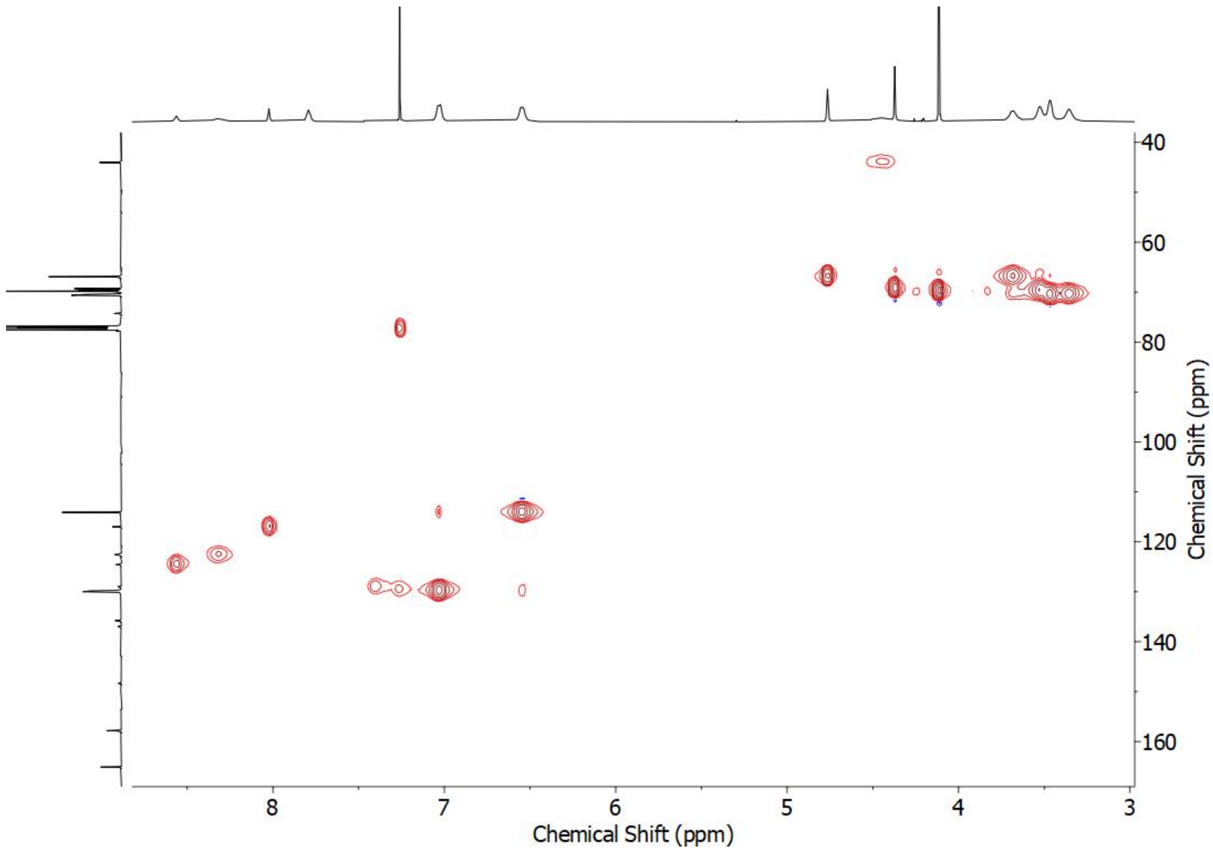

Figure S65 HSQC ($CDCl_3$) of **4^Fc^**

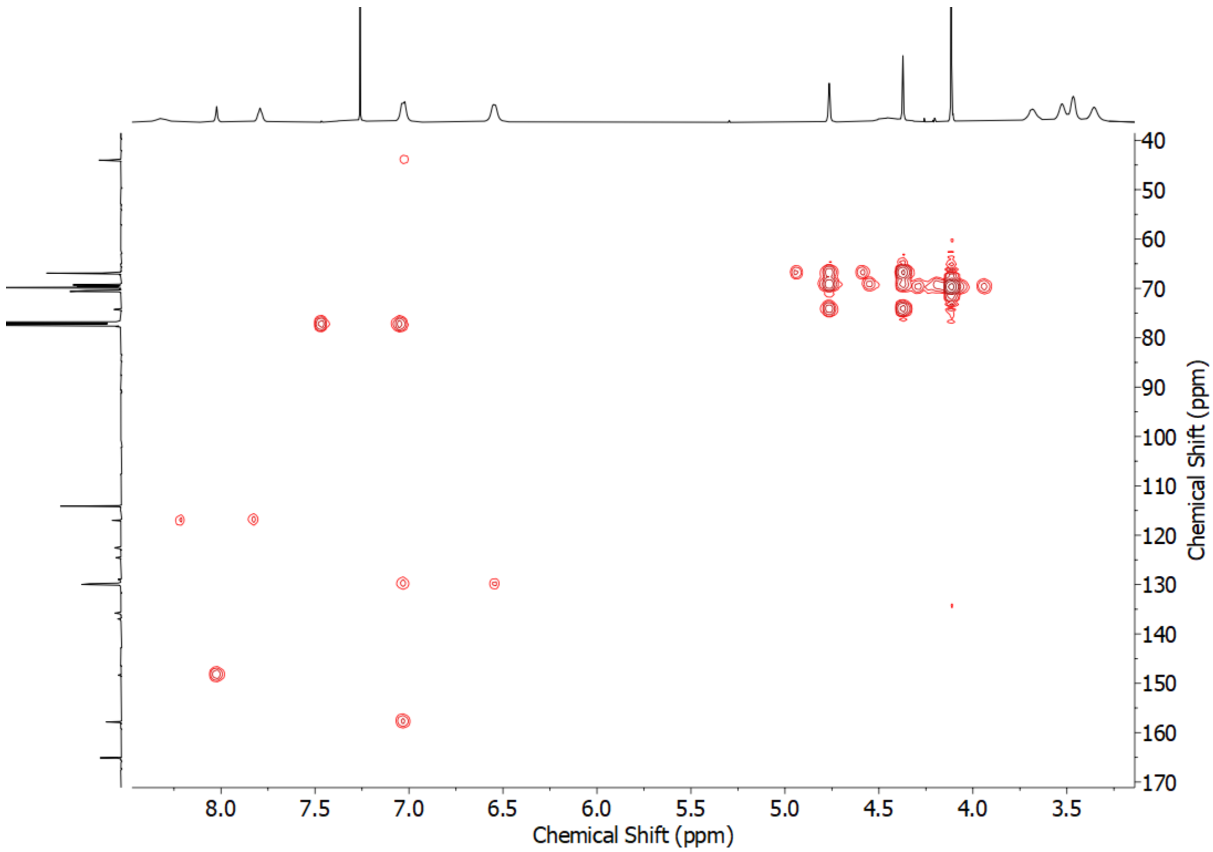

Figure S66 HMBC ($CDCl_3$) of **4^Fc^**

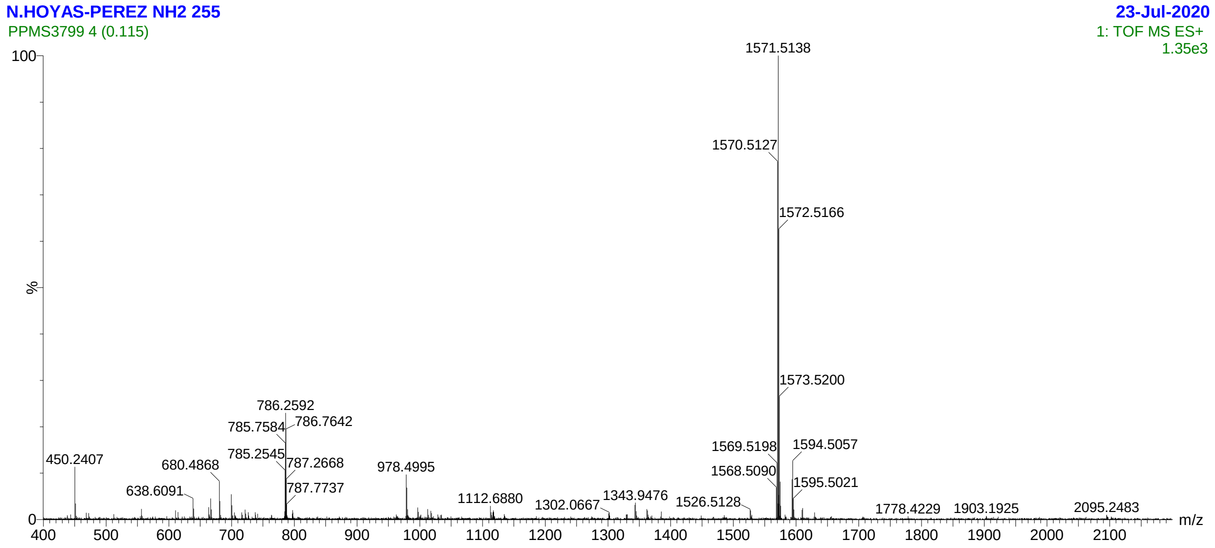

Figure S67 HR-ESI-MS of **4**$^{\mathbf{Fc}}$

3. Spectroscopic and Photophysical Data of 3^{Py} & 4^{Py}

Experimental procedures

Steady-State Absorption and Fluorescence Spectra

Absorption and fluorescence spectra were recorded using a HP 8453 spectrophotometer (Agilent) and FluoroMax-4 spectrophotometer (Jobin-Yvon, Horiba), respectively. Samples were excited at 340 nm and fluorescence spectra recorded in the range 350-650 nm and corrected for the wavelength sensitivity of detector. Fluorescence quantum yields (Φ_{fl}) were determined relatively to quinine sulfate in 1 N H_2SO_4 (0.546).[3] QY values were corrected for the refractive indexes of organic solvents and the aqueous solution used for preparation of samples and the standard, respectively. All spectra were recorded using a 10 mm quartz cuvette.

Fluorescence spectra at different temperatures were recorded with the automated Peltier-based thermostat F3004 (Jobin-Yvon, Horiba) in a temperature range of 10 – 60/80 °C depending on the boiling point of the solvent. Cyclic measurements of spectra at 10 and 70 °C were carried out in the automated mode with 15 min of waiting times for the equilibration of sample temperature at each point.

Time-Resolved Fluorescence Measurements

Fluorescence time profiles were measured using time-correlated single-photon counting (TCSPC) setup based on DeltaFlex TCSPC (Horiba) with the excitation source 282 nm NanoLED (Horiba). Depending on the decay time, the time window of 100 – 800 ns was used with 4096 time bins. The fluorescence time profiles had 10 000 counts at the peak. Time profiles were fitted using a sum of exponential function numerically convolved with the Instrument Response Function, which was recorded using a scattering Ludox® solution. In the case of $\mathbf{3^{Py}}$ the mono exponential function was used; in the case of $\mathbf{4^{Py}}$ a global fitting of several time profiles recorded over the emission band were reproduced with the sum of three exponential functions (the same time constants were used for all time profiles). The goodness of fits was controlled by visual agreement between experimental and fitted kinetics and confirmed by the value of χ^2 parameter in the range below 1.2.

Spectroscopic and Photophysical Data

Table S1 Fluorescence quantum yields (Φ_{fl}) and lifetimes (τ) for $\mathbf{3^{Py}}$ (5.2×10^{-6} M) and $\mathbf{4^{Py}}$ (2.6×10^{-6} M) in air-equilibrated organic solvents. The mono-exponential function was used to reproduce the fluorescence decays of $\mathbf{3^{Py}}$ (see Fig. S68 for kinetics) and the three-exponential global analysis of decays recorded over the whole emission spectrum – to $\mathbf{4^{Py}}$ (see Fig. S72 for kinetics): the first component, τ_M, corresponded to a decay of monomeric form and the second and the third, τ_{e1} and τ_{e2}, – to a decay of two forms of excimers. The three-exponential function was also used in the case of $\mathbf{3^{Py}}$ in MeOH due to visible signs of excimer formation in protic solvents, see Fig. 1a for spectrum and Fig. S69 for kinetics; the major and fastest component is present in Table. Standard error for Φ_{fl} is 10%.

	$\mathbf{3^{Py}}$		$\mathbf{4^{Py}}$			
Solvent	Φ_{fl}	τ / ns	Φ_{fl}	τ_M / ns	τ_{e1} / ns	τ_{e2} / ns
Toluene	0.42	12.1	0.43	7.5	15.0	20.7
CH_3Cl	0.37	8.6	0.39	7.7	16.0	27.0
THF	0.27	13.8	0.29	7.6	14.3	28.6
DMF	0.45	17.4	0.44	9.1	17.7	32.7
DMSO	0.60	17.8	0.65	13.2	23.8	62.1
MeCN	0.17	11.6	0.21	3.8	12.3	14.2
MeOH	0.007	0.35	0.039	0.35	8.6	22.1

The Φ_{fl} values for both molecules are close together in broad range of solvents from toluene to DMSO with a significant increase of Φ_{fl} for $\mathbf{4^{Py}}$ in MeCN and MeOH relatively to $\mathbf{3^{Py}}$. Substantial lowering of Φ_{fl} for both $\mathbf{3^{Py}}$ and $\mathbf{4^{Py}}$ in MeOH should be attributed to intermolecular H-bonds, which are known to quench fluorescence intensity via effective non-radiative transitions.[4-6]

The τ values for $\mathbf{3^{Py}}$ are shorter than values reported for pyrene[7] due to the loss of symmetry upon pyrene linking to macrocycle. However, the τ values for excimers are close with values reported for pyrene[7] and other excimer-forming pyrene derivatives reported to date.[8,9] Interesting feature of $\mathbf{4^{Py}}$ excimers is a two-component decay, which was not reported earlier and should be assigned to excimers with two different geometries.

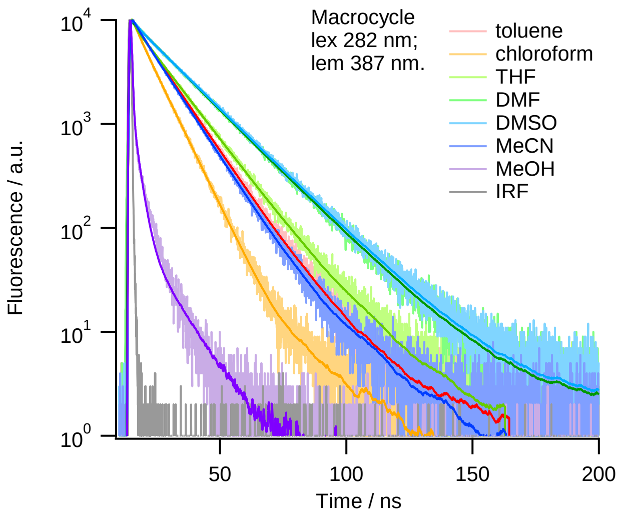

Figure S68 Fluorescence time profiles recorded with **3**^{Py} (5.2 × 10^{-6} M) in various air-equilibrated solvents at room temperature. The excitation wavelength 282 nm, the emission was recorded at 387 nm. Smooth lines are the best fits using mono exponential function except for MeOH for which three exponential fit was used.

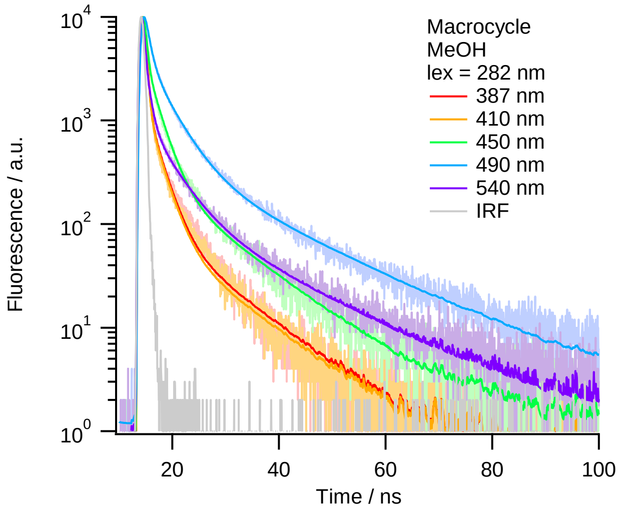

Figure S69 Fluorescence time profiles recorded with **3**^{Py} (5.2 × 10^{-6} M) in air-equilibrated MeOH at room temperature. The excitation wavelength 282 nm, the emission wavelengths are denoted on plots. Smooth lines are the best fits using three-exponential global analysis.

It should be noted that **3**^{Py} is able to form excimers in MeOH leading to an increase of intensity at the red side of emission spectrum as compared with other solvents (Fig. 1A). The presence of excimer emission was supported by time-resolved measurements (Fig. S69) demonstrating the presence of long-lived components with the maximal contribution at 490 nm, the maximum of excimer emission band. The formation of excimers is more pronounced for aqueous solution, where only excimer band was observed for both **3**^{Py} and **4**^{Py} (Fig. S70). No monomer emission in aqueous environment clearly demonstrates the aggregation-like behavior of **3**^{Py} and **4**^{Py} in attempt to minimize the number of pyrene contacts with water molecules.

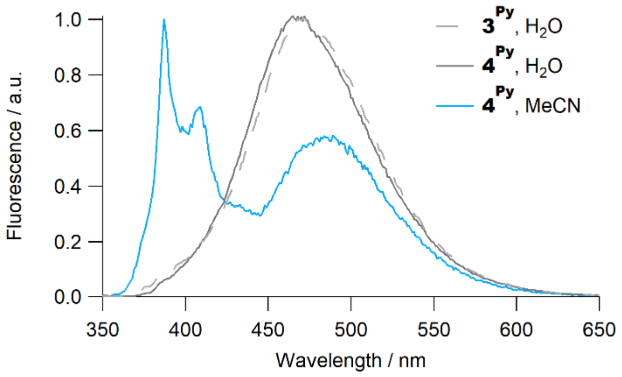

Figure S70 Normalized emission spectra recorded with **3^Py** (5.2×10^{-6} M) and **4^Py** (2.6×10^{-6} M) in H_2O and MeCN.

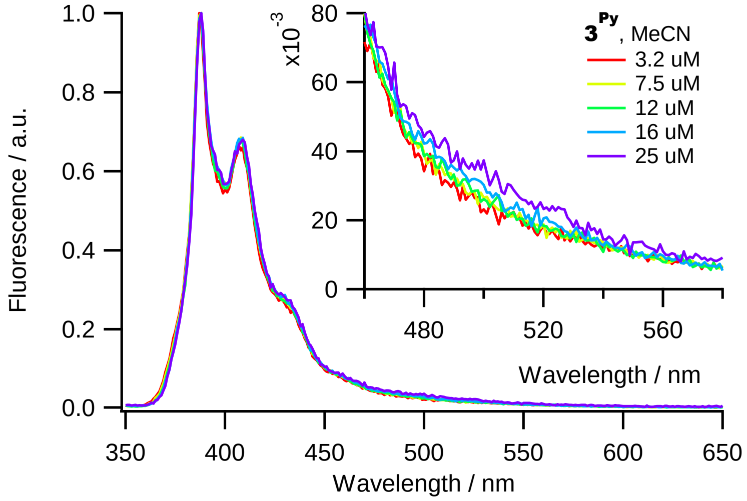

Figure S71 Normalised fluorescence spectra of **3^Py** (MeCN) at various concentrations

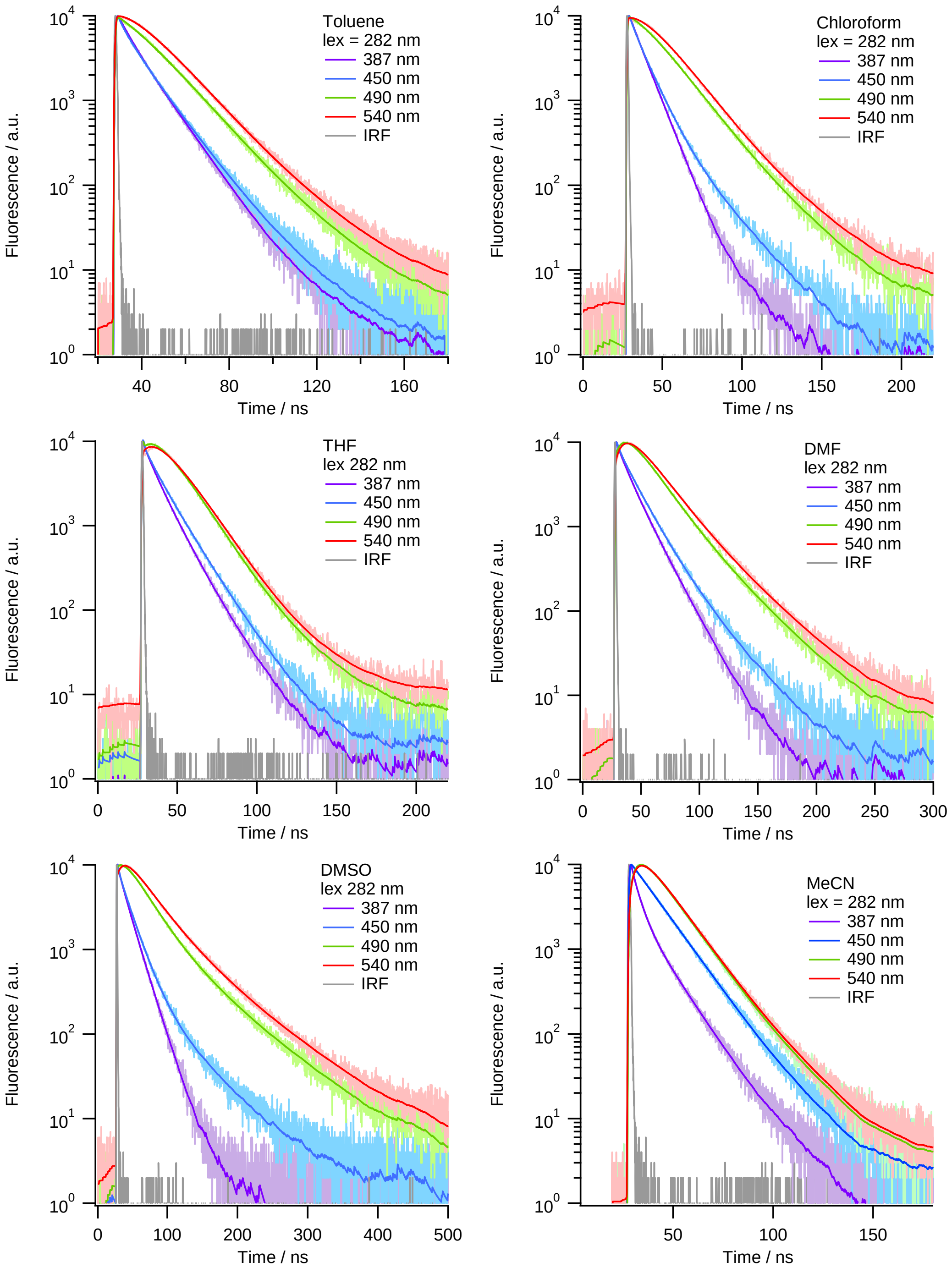
Toluene
lex = 282 nm
387 nm
450 nm
490 nm
540 nm
IRF
Chloroform
lex = 282 nm
387 nm
450 nm
490 nm
540 nm
IRF
THF
lex 282 nm
387 nm
450 nm
490 nm
540 nm
IRF
DMF
lex 282 nm
387 nm
450 nm
490 nm
540 nm
IRF
DMSO
lex 282 nm
387 nm
450 nm
490 nm
540 nm
IRF
MeCN
lex = 282 nm
387 nm
450 nm
490 nm
540 nm
IRF
Fluorescence / a.u.
Time / ns

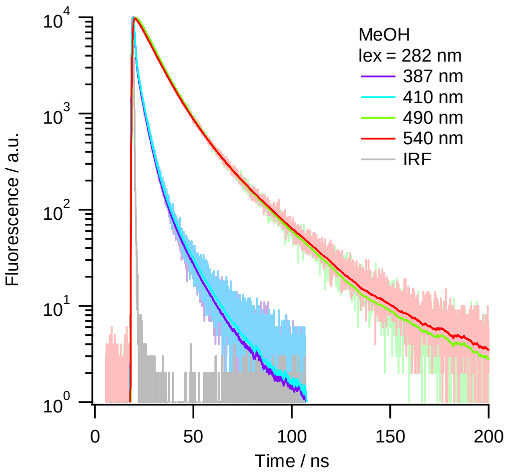

Figure S72 Fluorescence time profiles recorded with **4^Py^** (2.6×10^{-6} M) in various air-equilibrated solvents at room temperature. The excitation wavelength 282 nm, the emission wavelengths are denoted on plots. Smooth lines are the best fits using three-exponential global analysis.

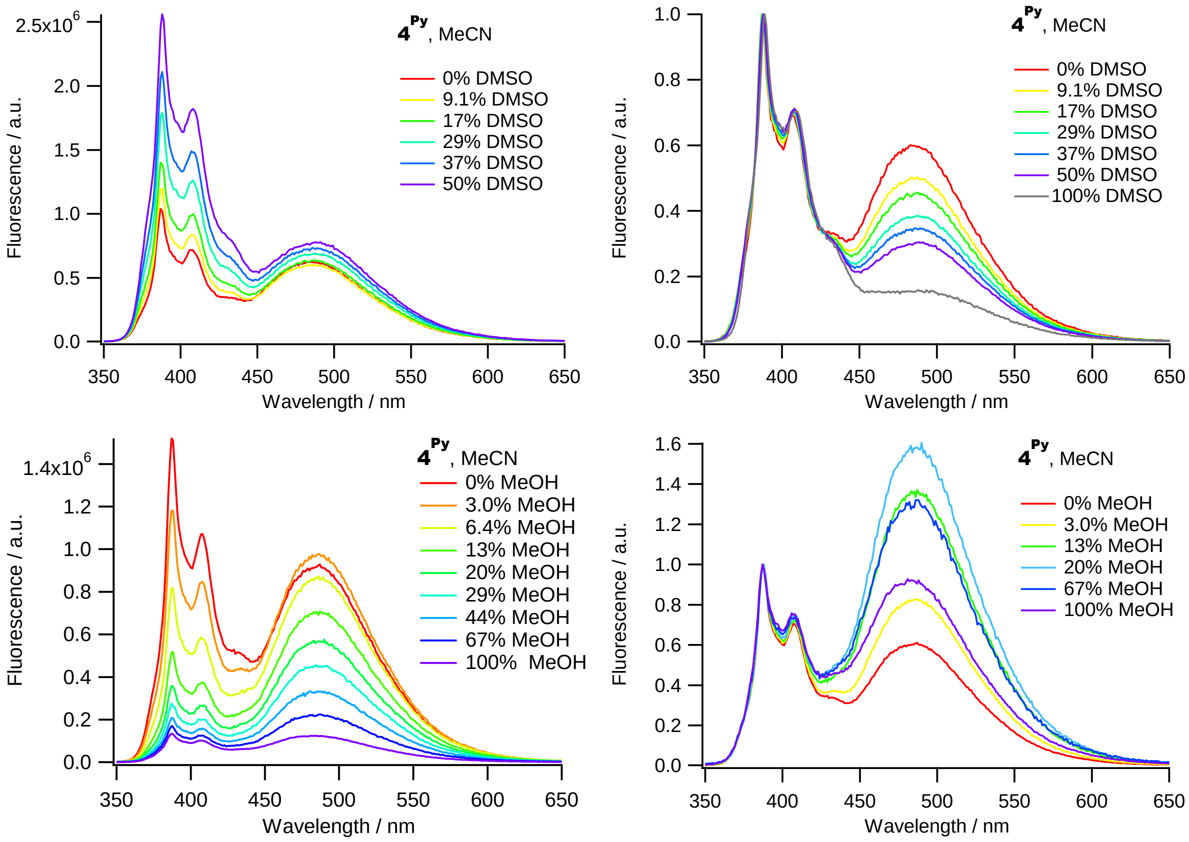

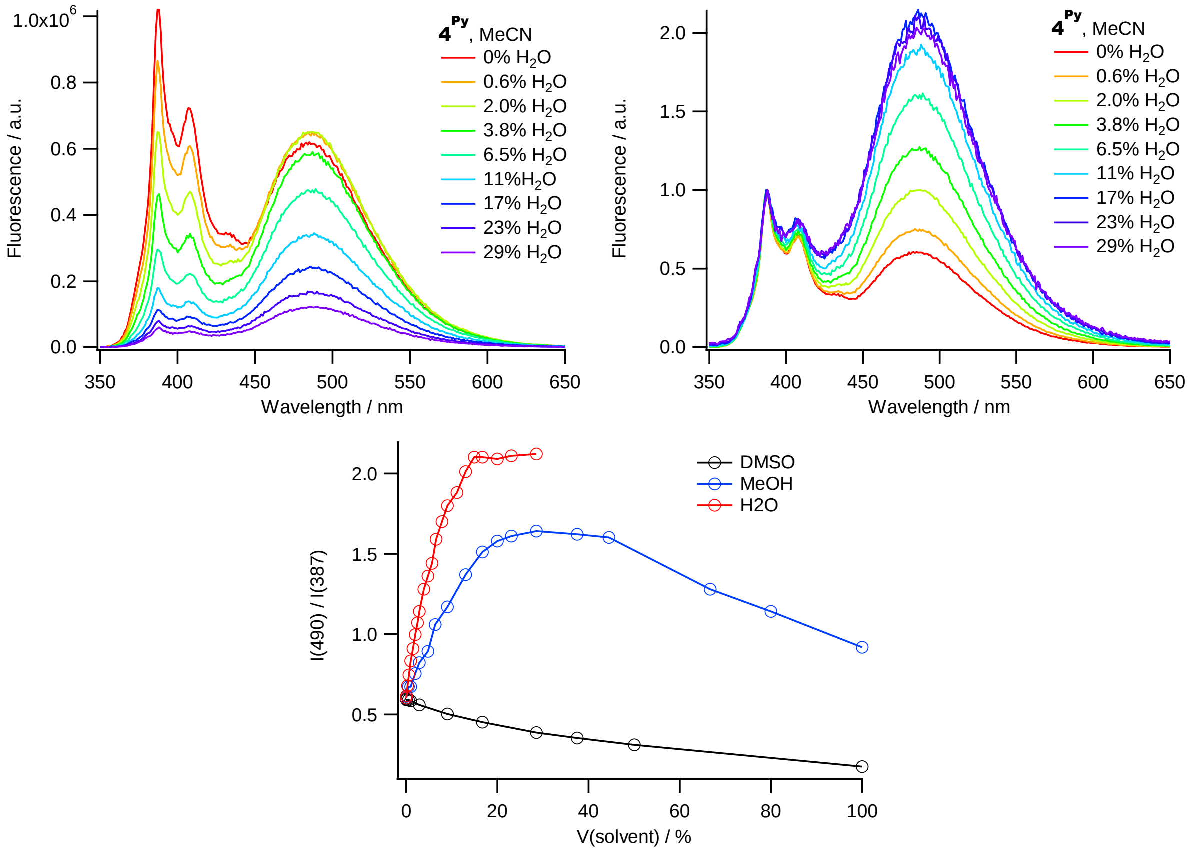

Figure S73 Fluorescence spectra before (left) and after (right) normalisation recorded with **4**^Py^ (2.6×10^{-6} M) in MeCN with addition of (top two) DMSO, (middle two) MeOH and (bottom two) H_2O. The single bottom plot: the ratio of I490 / I387 vs content of the second component of mixture (v/v).

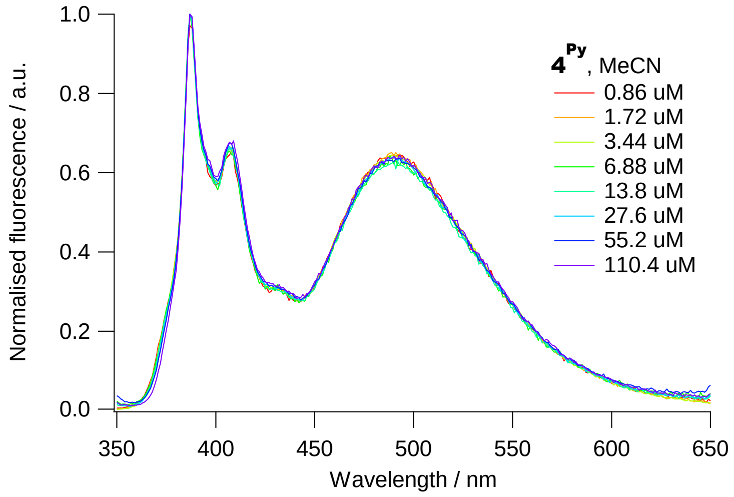

Figure S74 Normalised fluorescence spectra of **4**^{Py} (MeCN) at various concentrations.

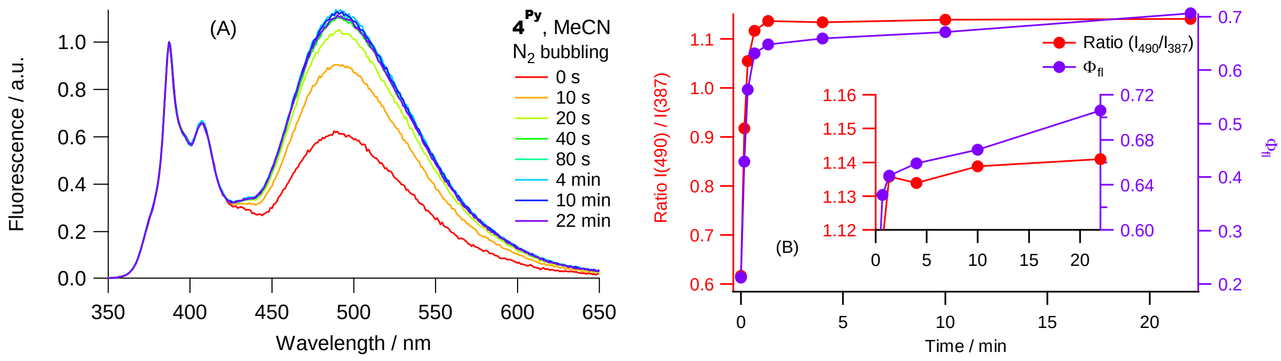

Figure S75 Data obtained with **4^{Py}** (2.6 × 10^{-6} M) in MeCN during the N_2 bubbling: (A) normalized emission spectra, (B) ratio of fluorescence intensities at 490 and 387 nm and fluorescence quantum yields (Φ_{fl}).

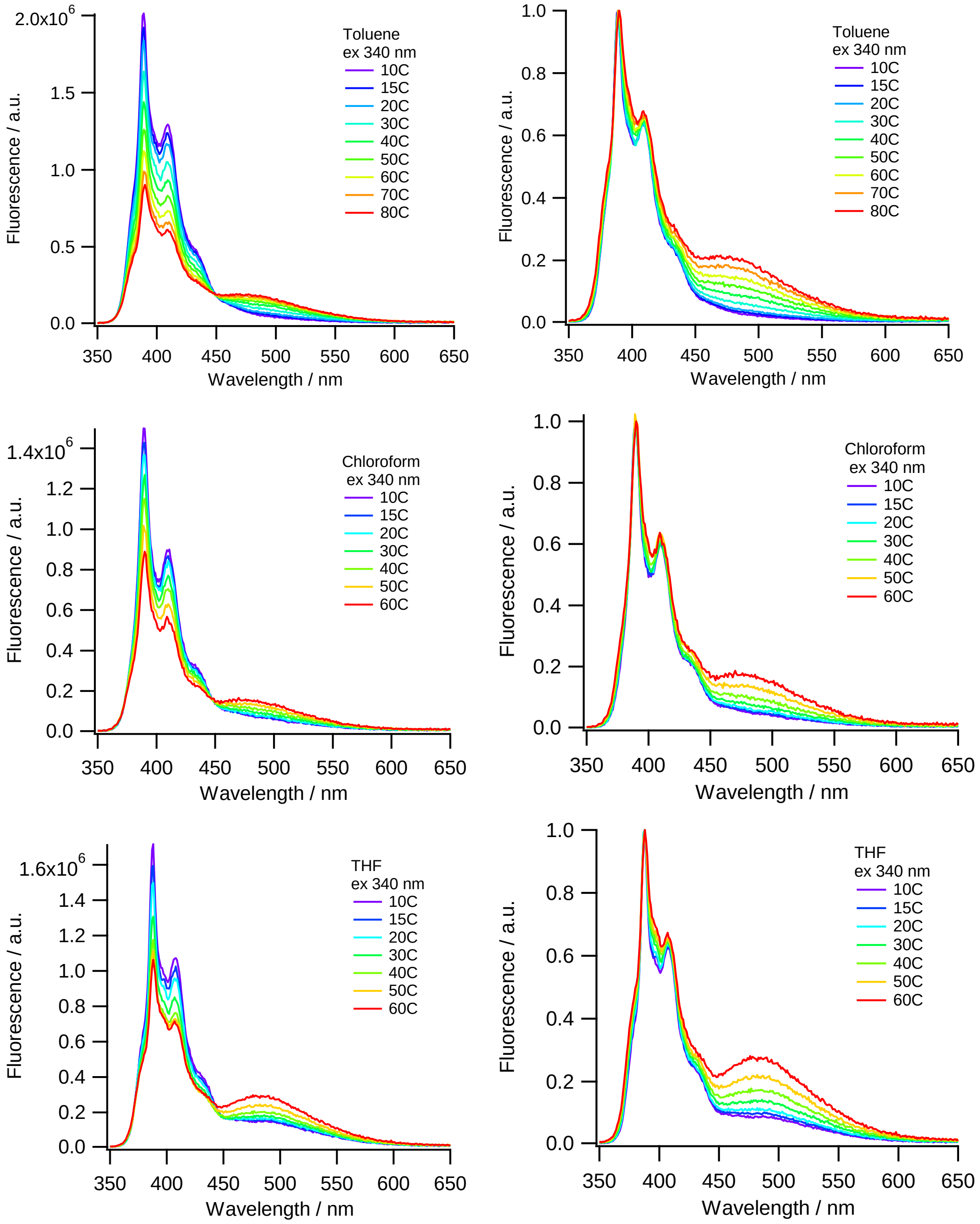

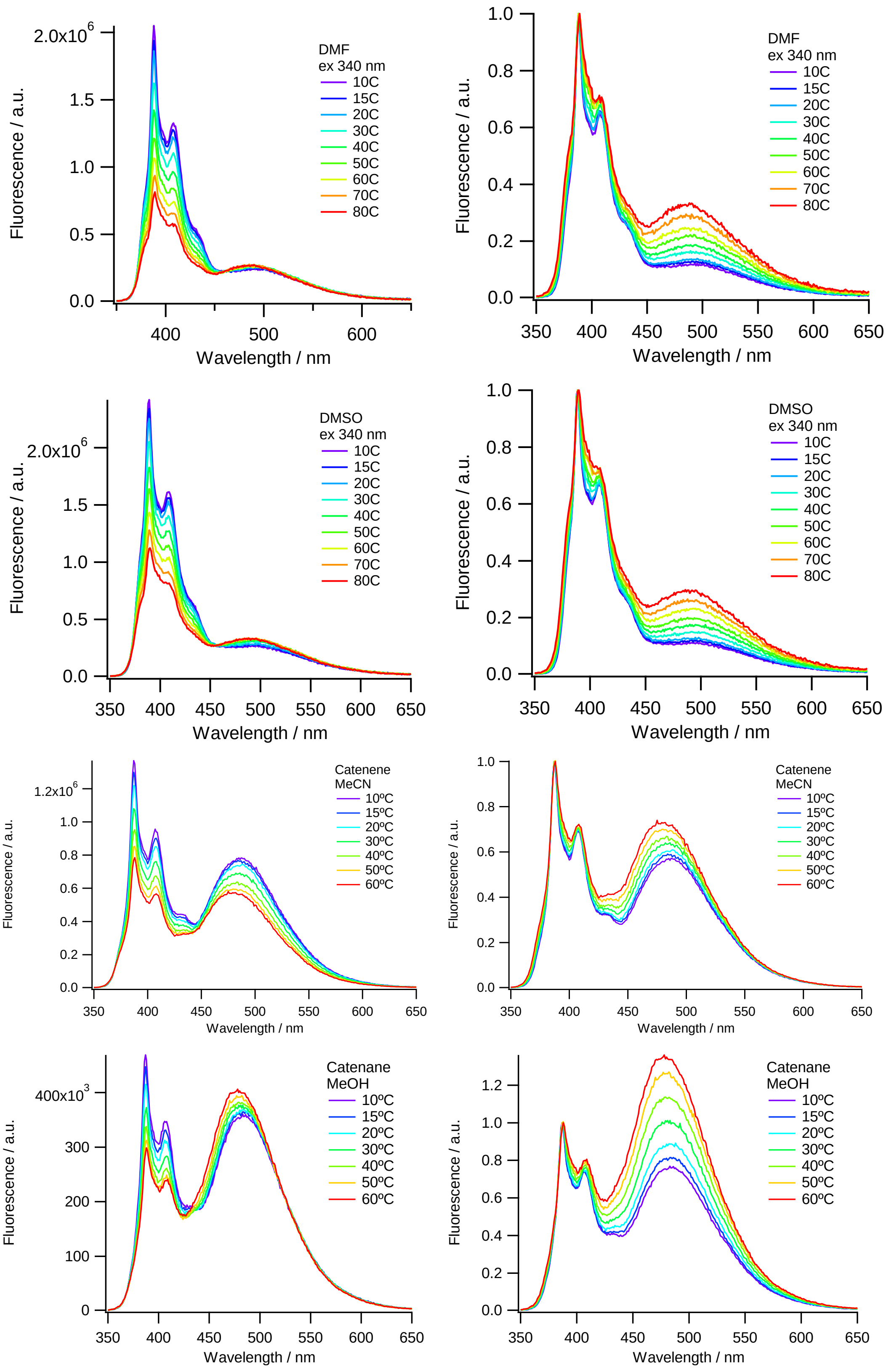

Figure S76 Fluorescence spectra before (left) and after (right) normalization recorded with **4^{Py}** (2.6 × 10^{-6} M) in organic solvents at different temperatures.

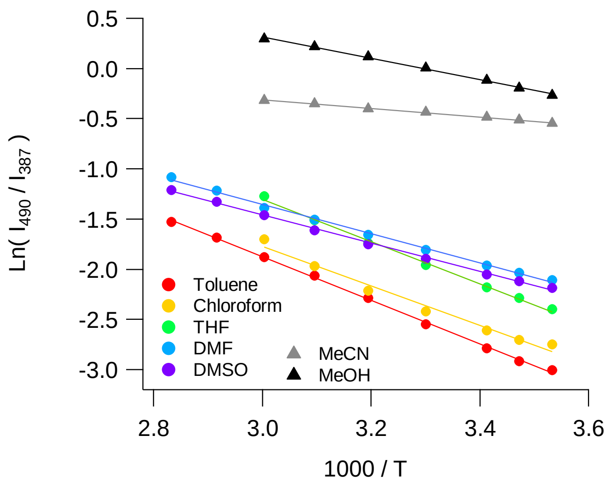

Figure S77 Plot of Ln(I490 / I387) vs 1/T using fluorescence spectra recorded with $\mathbf{4}^{Py}$ (2.6×10^{-6} M) in MeCN on Fig. S76 (left).

The ratio between monomer and excimer bands was shown to be affected by temperature (Fig. 3B and S76). This indicates the temperature dependence for the rate constants of excimer formation (k_{ME}) and dissociation (k_{EM}), as observed for unsubstituted pyrene in solution.[7] Assuming no temperature dependence for the rate constants of other $\mathbf{4}^{Py}$ fluorescence decays, including radiative and non-radiative for monomer and excimer, as with unsubstituted pyrene,[7] the differences in activation energies $\Delta E_a = E_{ME} - E_{EM}$ could be extracted from linear dependences of $\log(I_{490}/I_{387})$ vs 1/T for all used solvents (Fig. S77); the obtained values are listed in Table S2. According to literature data the activation energies for the viscosity of used solvents ($\Delta E_a^{solv\text{-}visc}$) are in the range of 12-14 kJ/mol.[10] The obtained ΔE_a values (Table S2) could be above (low polar solvents) or below (MeCN and MeOH) $\Delta E_a^{solv\text{-}visc}$ values that indicates more significant contribution of the pyrene-pyrene and pyrene-solvent interactions in the stability of $\mathbf{4}^{Py}$ excimers rather than movements of solvent molecules as a bulk media.

Table S2 The values of ΔE_a determined from linear fit of $\mathrm{Ln}(I_{490} / I_{387})$ vs 1/T.

Solvent	ΔE_a, kJ / mol
Toluene	18.0
CH_3Cl	16.4
THF	17.5
DMF	12.1
DMSO	11.7
MeCN	3.5
MeOH	8.9

4. Electrochemical data for 3^{Fc} and 4^{Fc}

Cyclic Voltammetry was performed using a BioLogic SP-150 potentiostat. The CV setup consisted of a glassy carbon working electrode (Pine Instruments, 3 mm OD), a Pt wire counter electrode and an Ag wire quasi-reference electrode. All measurements were performed under an Ar atmosphere, and each measurement was referenced to an internal reference, Fc/Fc$^+$. A 0.1 M $TBAPF_6$/MeCN or a $TBAPF_6/CH_2Cl_2$ (TBA = nBu$_4$N$^+$) electrolyte was used for cyclic voltammetry measurements (solvent stated in the caption of the relevant figures). Sample concentrations are reported in the figure captions. For all measurements, a CV of the background electrolyte was measured prior to addition of the analyte to confirm no background impurities are present. The voltage range investigated was set to be within the electrochemical window of the electrolyte. In all cases multiple sweeps were performed, which displayed no differences in the voltammogram between scans.

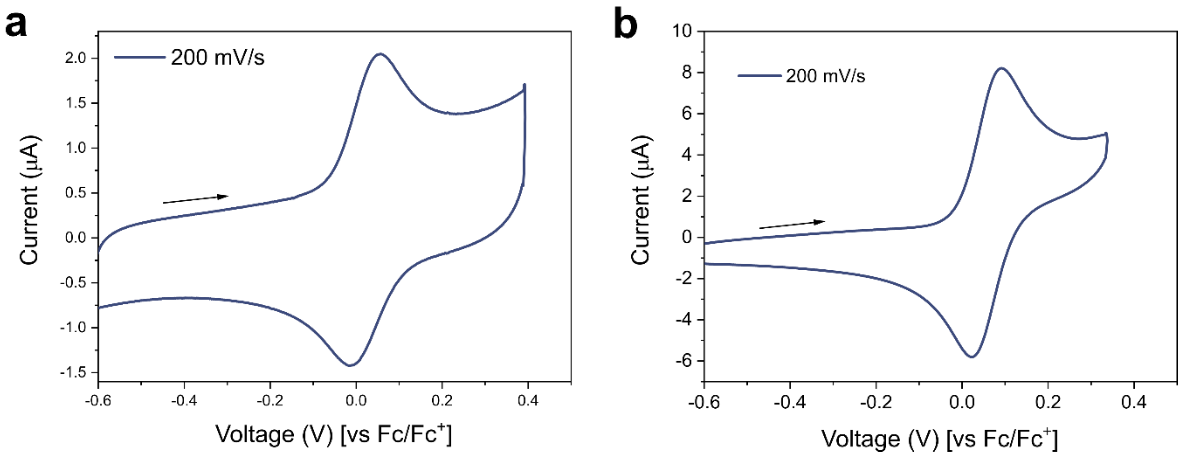

Figure S78 The cyclic voltammograms of **a**, catenane $\mathbf{4^{Fc}}$ (0.1 mg/mL) and **b**, macrocycle $\mathbf{3^{Fc}}$ (0.2 mg/mL). The measurements were performed under Ar in a 0.1 M electrolyte solution comprising of $TBAPF_6$/MeCN at 298 K at a scan rate of 200 mV/s. The traces displayed here correspond to the first measurement of a freshly polished glassy carbon electrode (3 mm OD). The potential is referenced to the Fc/Fc+ redox couple which is added at the end of the measurement. The black arrows indicate the direction of the initial scan. An oxidation potential of 0.023 V and 0.057 V (vs Fc/Fc+) was determined for **a** and **b**, respectively.

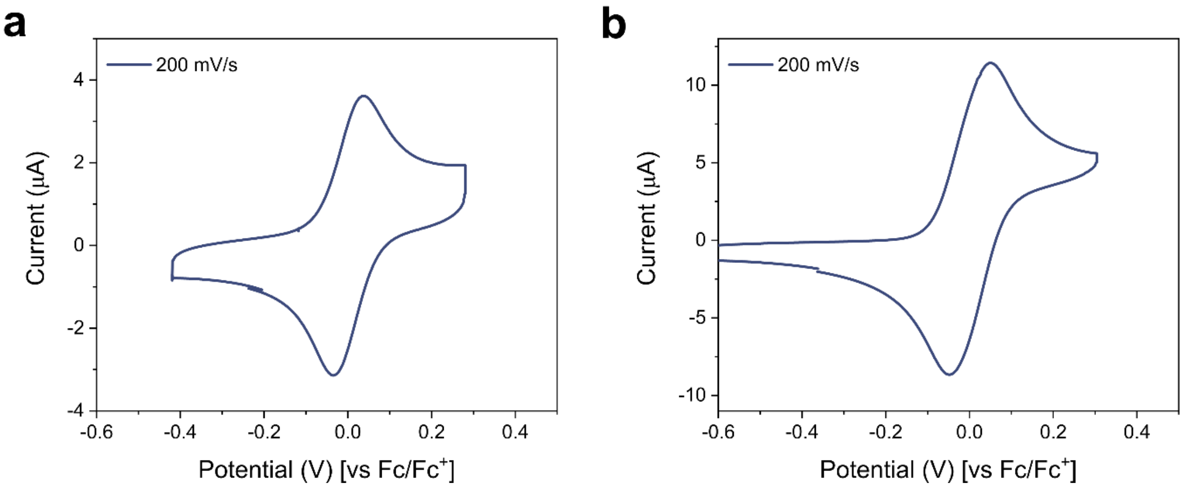

Figure S79 The cyclic voltammograms of **a**, catenane $\mathbf{4^{Fc}}$ (0.2 mg/mL) and **b**, macrocycle $\mathbf{3^{Fc}}$ (0.2 mg/mL). The measurements were performed under Ar in a 0.1 M electrolyte solution comprising of $TBAPF_6/CH_2Cl_2$ at 298 K at a scan rate of 200 mV/s. The traces displayed here correspond to the first measurement of a freshly polished glassy carbon electrode (3 mm OD). The potential is referenced to the Fc/Fc+ redox couple which is added at the end of the measurement. An oxidation potential of 0.041 V and 0.077 V (vs Fc/Fc+) was determined for **a** and **b**, respectively.

5. Photoirradiation data for 3^{An} and 4^{An}

Samples were irradiated using a Nichia NCSU276A 365 nm LED (365 nm, 800 mW @ 100% power) operating at 5% power, fitted with a collimating lens. The cuvette was placed 3.5 cm away from the light source.

UV-vis absorption spectra were obtained on a Cary 60 UV-Vis spectrophotometer equipped with a temperature controller operating at 298 K. Samples were prepared in a UV Quartz cuvette with a path length of 10 mm. Solutions of the compounds were prepared in HPLC or higher-grade solvents. A background measurement containing only the solvent was recorded before measuring samples. This background was subtracted from the sample data using Origin Software.

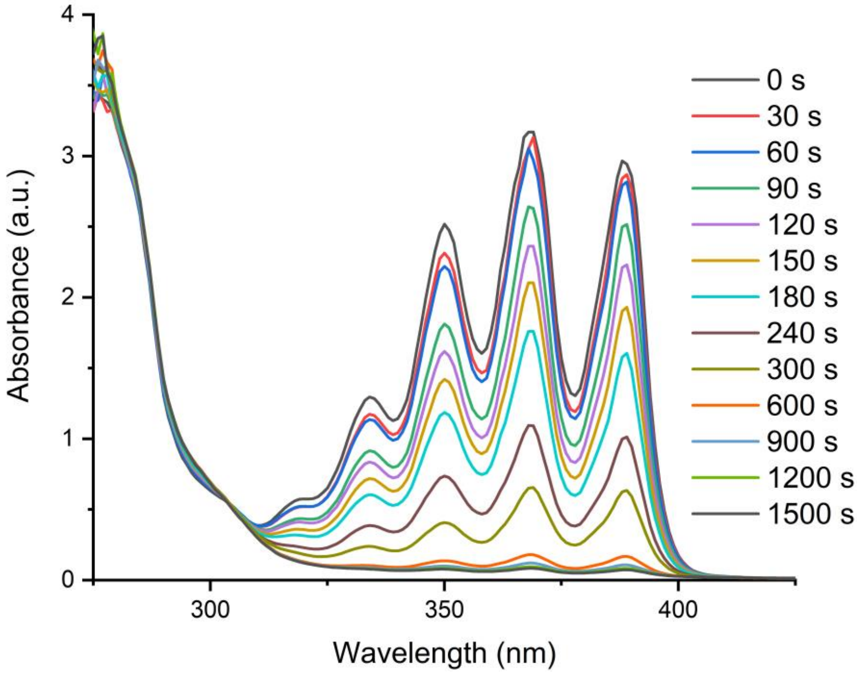

Figure S80 UV-Vis absorption spectra of $\mathbf{3^{An}}$ (5×10^{-4} M in DMSO, 298 K) upon irradiation with 365 nm light for 0 to 1500 s.

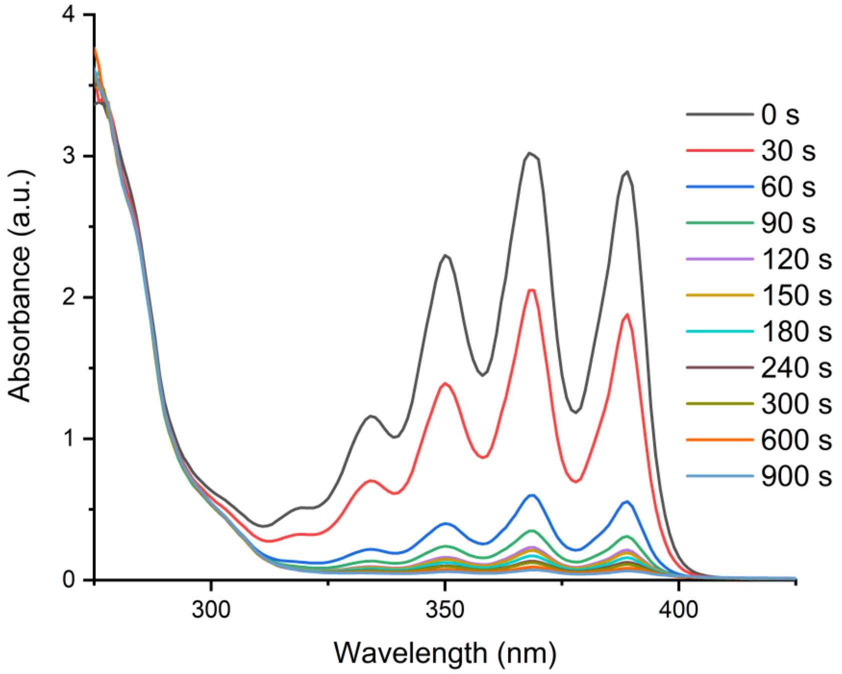

Figure S81 UV-Vis absorption spectra of $\mathbf{4^{An}}$ (2.5×10^{-4} M in DMSO, 298 K) upon irradiation with 365 nm light for 0 to 900 s.

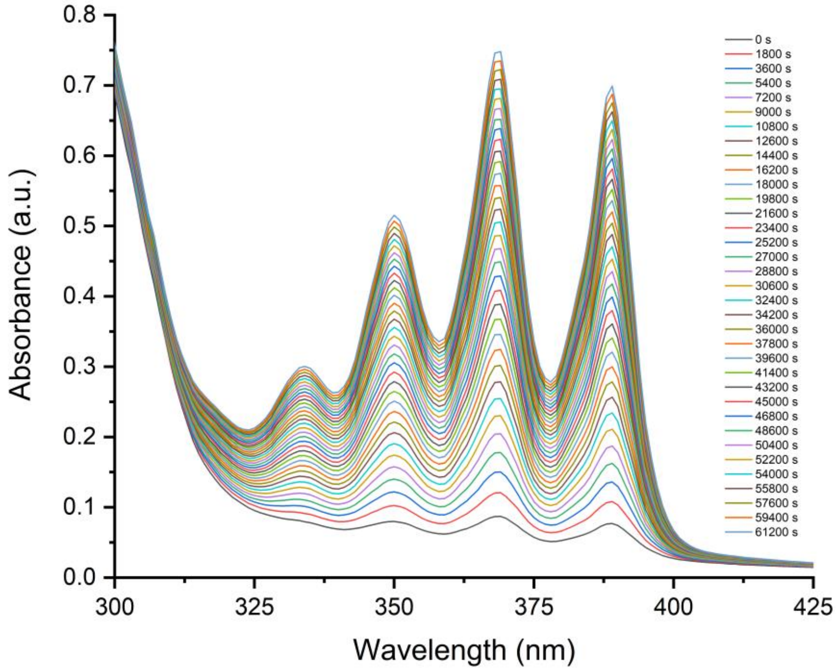

Figure S82 UV-Vis absorption spectra of $\mathbf{3}^{\mathbf{An}}$ (5×10^{-4} M in DMSO, 298 K) following irradiation (0 to 17 h).

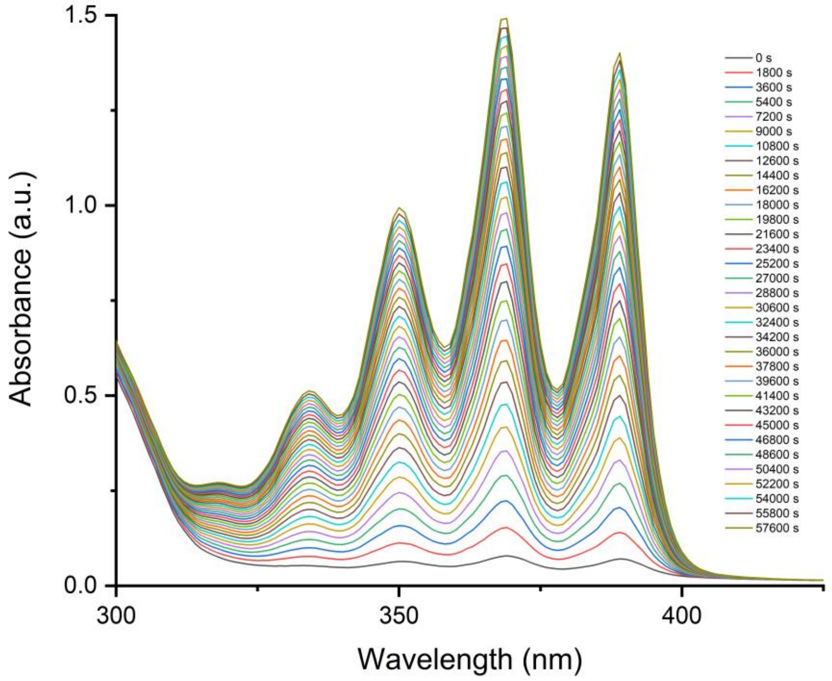

Figure S83 UV-Vis absorption spectra of $\mathbf{4}^{\mathbf{An}}$ (2.5×10^{-4} M in DMSO, 298 K) following irradiation (0 to 16 h).

Table S3 Anthracene photodimerisation of **3^{An}** and **4^{An}** monitored by UV-Vis absorption upon irradiation at 365 nm in N_2-purged DMSO solution (5 × 10^{-4} M with respect to the anthracene moiety).

	Macrocycle		Catenane	
**Time (s)**	Absorbance (a.u.)[a]	Conversion (%)	Absorbance (a.u.)[a]	Conversion (%)
**0**	2.94299	0	2.8902	0
**30**	2.86891	2.5	1.88026	34.9
**60**	2.81724	4.3	0.55625	80.8
**90**	2.51625	14.5	0.30977	89.3
**120**	2.2312	24.2	0.2135	92.6
**150**	1.93141	34.4	0.19282	93.3
**180**	1.60531	45.5	0.15954	94.5
**240**	1.0132	65.6	0.12678	95.6
**300**	0.63523	78.4	0.11188	96.0
**600**	0.16867	94.3	0.08495	97.1
**900**	0.10841	96.3	0.06532	97.7
**1200**	0.08437	97.1	-	-
**1500**	0.07339	97.5	-	-

[a]Absorbance at 389 nm

Table S4 Thermal cycloreversion of **3^{An}** and **4^{An}** monitored by UV-Vis absorption after irradiation in N_2-purged DMSO solution (5 × 10^{-4} M with respect to the anthracene moiety).

	Macrocycle		Catenane	
**Times (s)**	Absorbance (a.u.)[a]	Reversion (%)[b]	Absorbance (a.u.)[a]	Reversion (%)[b]
**0**	0.07705	2.6	0.07103	2.5
**1800**	0.10819	3.7	0.14062	4.9
**3600**	0.13595	4.6	0.20646	7.1
**5400**	0.16245	5.5	0.26979	9.3
**7200**	0.18724	6.4	0.33088	11.4
**9000**	0.21128	7.2	0.38985	13.5
**10800**	0.23436	8.0	0.44620	15.4
**12600**	0.25676	8.7	0.50096	17.3
**1400**	0.27842	9.5	0.55409	19.2
**16200**	0.30004	10.2	0.60503	20.9
**18000**	0.32078	10.9	0.65447	22.6
**19800**	0.34100	11.6	0.70275	24.3
**21600**	0.36092	12.3	0.74975	25.9
**23400**	0.37984	12.9	0.79431	27.5
**25200**	0.39907	13.6	0.83641	28.9
**27000**	0.41774	14.2	0.87940	30.4
**28800**	0.43530	14.8	0.91970	31.8
**30600**	0.45304	15.4	0.95867	33.2
**32400**	0.47059	16.0	0.99670	34.5
**34200**	0.4881	16.6	1.03328	35.8
**36000**	0.50419	17.1	1.0683	37.0
**37800**	0.52016	17.7	1.10150	38.1
**39600**	0.53630	18.2	1.13425	39.2
**41400**	0.55223	18.8	1.16700	40.4
**43200**	0.56668	19.3	1.19642	41.4
**45000**	0.58123	19.7	1.22555	42.4
**46800**	0.59632	20.3	1.25205	43.3
**48600**	0.60980	20.7	1.27957	44.3

**50400**	0.62310	21.2	1.30511	45.2
**52200**	0.63741	21.7	1.33200	46.1
**54000**	0.64985	22.1	1.35642	46.9
**55800**	0.66256	22.5	1.38090	47.8
**57600**	0.67534	22.9	1.40131	48.5
**59400**	0.68740	23.4	-	-
**61200**	0.69897	23.8	-	-

[a]Absorbance at 389 nm

[b]Calculated as percentage of absorbance value pre-irradiation

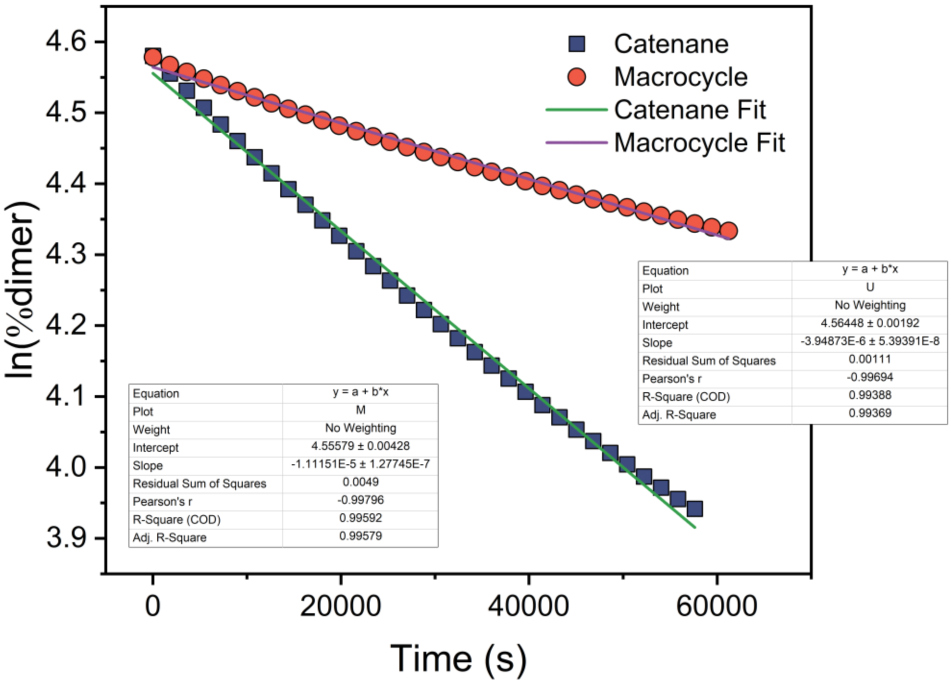

Figure S84 Plot of the thermal reversion of the dimerised **3**^{An} (Macrocycle) and **4**^{An} (Catenane) over time at 298 K. The %dimer was gauged by comparing the initial absorption of the sample at 389 nm prior to photoirradiation to the absorption of the fully photodimerised sample. The rate of thermal reversion, *k*, was determined from the gradient of the linear fits shown in the plot, and the thermal half-life at 298 K using the 1st order relationship $t_{1/2} = \ln(2)/k$.

Quantum Yield: Ferrioxalate actinometry was used to determine the photon flux from a Nichia NCSU276A 365 nm LED (365 nm, 800 mW @ 100% power) operating at 5% power, fitted with a collimating lens, following a previously reported procedure.[11] The cuvette was placed 3.5 cm away from the light source. The photon flux of the 365 nm LED operating at 5% power for our setup was determined to be 1.845×10^{16} photons/s. **3**^{An} non-dimerised ε_{365} 5600 M^{-1} cm^{-1}; **3**^{An} dimerised ε_{365} 145 M^{-1} cm^{-1}.**4**^{An} non-dimerised ε_{365} 5180 M^{-1} cm^{-1}; **4**^{An} dimerised ε_{365} 120 M^{-1} cm^{-1}.

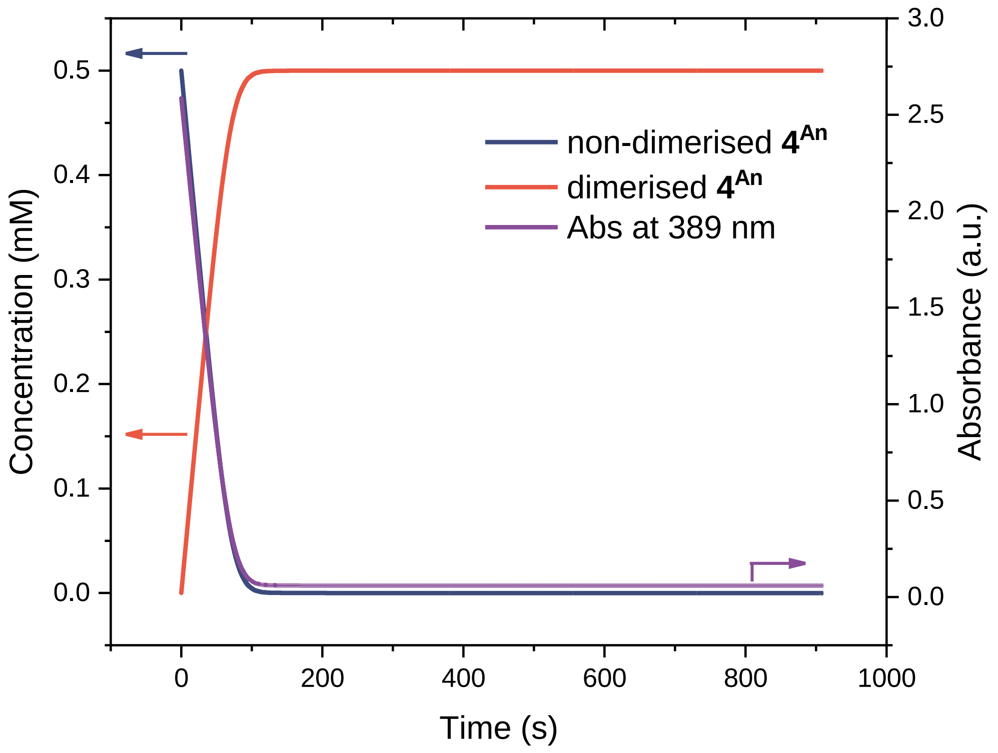

Figure S85 (left) Plot of concentration of non-dimerised and dimerised **4**^{An} as a function of irradiation time with 365 nm light, (right) the UV-vis absorbance at 389 nm as a function of irradiation time with 365 nm light. This was used to calculate the quantum yield of 365 nm irradiation. A quantum yield of 0.71 was measured for this sample.

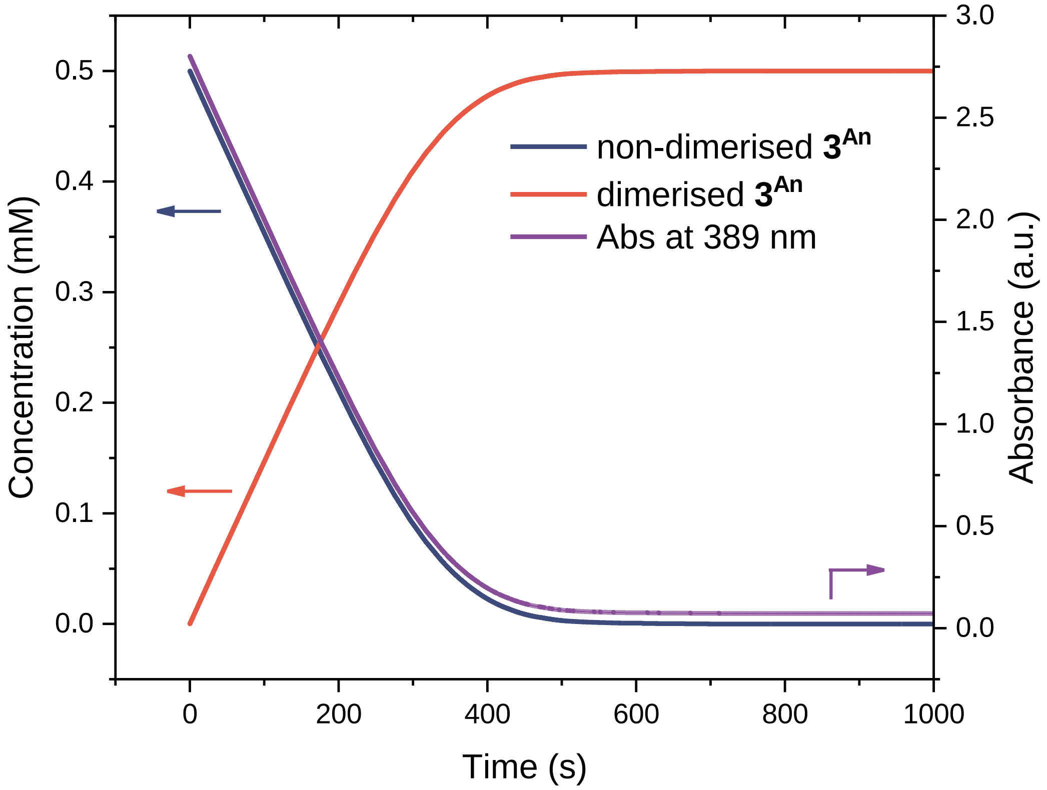

Figure S86 (left) Plot of concentration of non-dimerised and dimerised **3**$^{\mathbf{An}}$ as a function of irradiation time with 365 nm light, (right) the UV-vis absorbance at 389 nm as a function of irradiation time with 365 nm light. This was used to calculate the quantum yield of 365 nm irradiation. A quantum yield of 0.15 was measured for this sample.

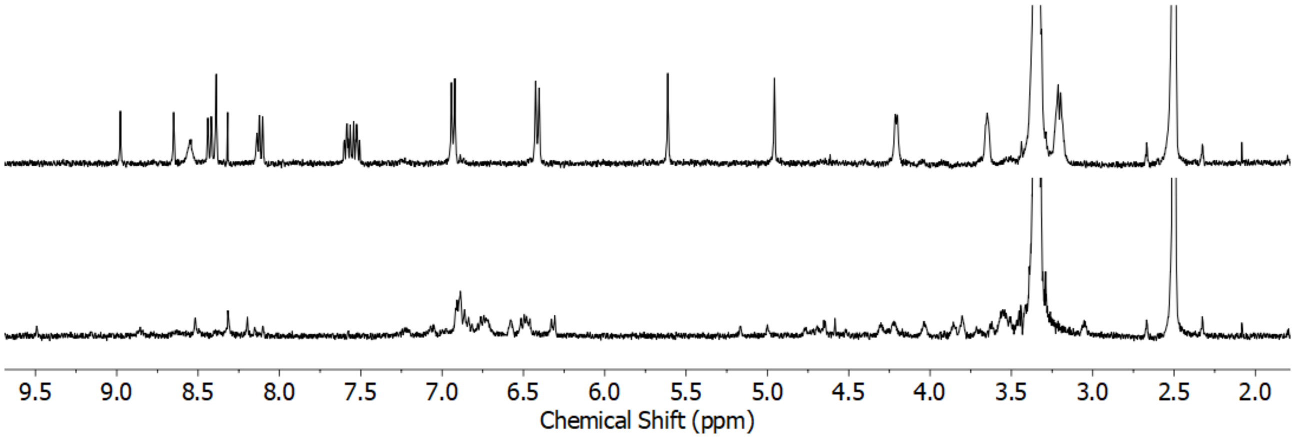

Figure S87 Partial ^{1}H NMR spectra (d_6-DMSO, 400 MHz, 10^{-3} M) of **4**$^{\mathbf{An}}$ before irradiation (top) and after irradiation at 365 nm for 90 minutes (bottom).

6. Computational studies of catenanes

We performed density functional theory (DFT) geometry optimisations to obtain the ground state of $\mathbf{4^{Ph}}$, $\mathbf{4^{Fc}}$, $\mathbf{4^{An}}$ and the HH- and HT-isomers of photodimerised $\mathbf{4^{An}}$. We used the B3LYP hybrid functional[12-15] as implemented in the Gaussian16 code,[16] including Grimme's D3 dispersion corrections.[17] The calculations were all-electron (i.e., no pseudopotentials were employed) and a 6-311G(d,p) basis set was used to expand the wavefunctions.

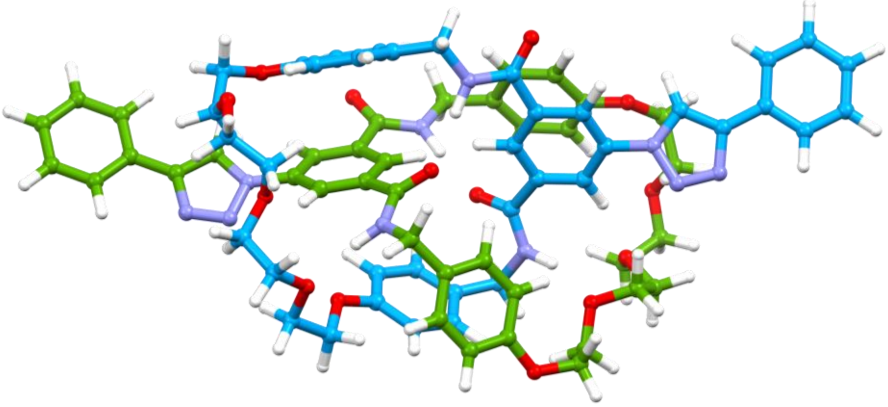

Figure S88 DFT-optimised structure of $\mathbf{4^{Ph}}$.

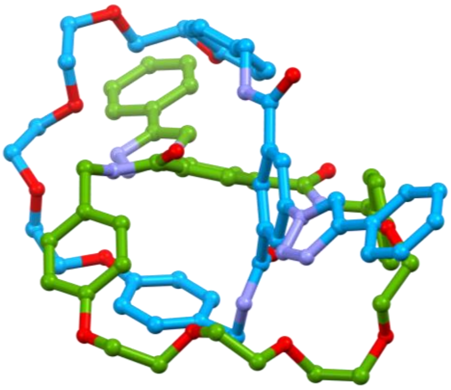

Figure S89 DFT-optimised structure of $\mathbf{4^{Ph}}$. Hydrogen atom omitted for clarity.

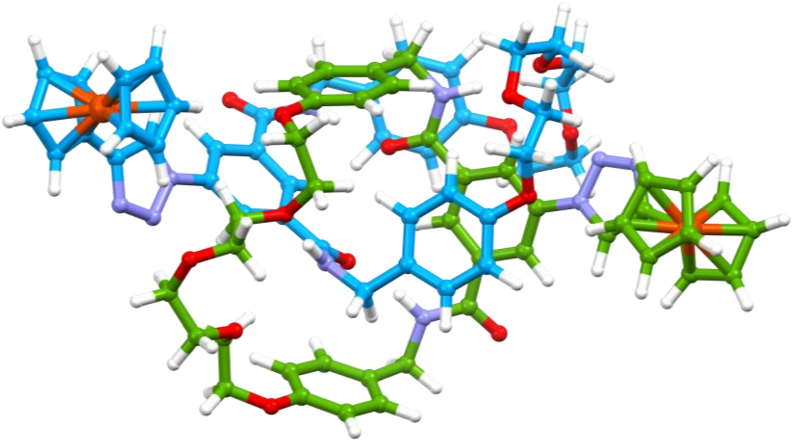

Figure S90 DFT-optimised structure of $\mathbf{4^{Fc}}$.

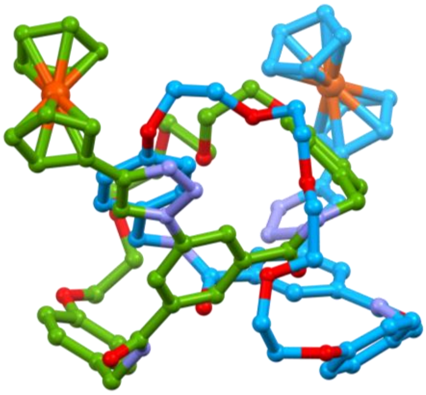

Figure S91 DFT-optimised structure of **4**$^{\mathbf{Fc}}$. Hydrogen atoms omitted for clarity.

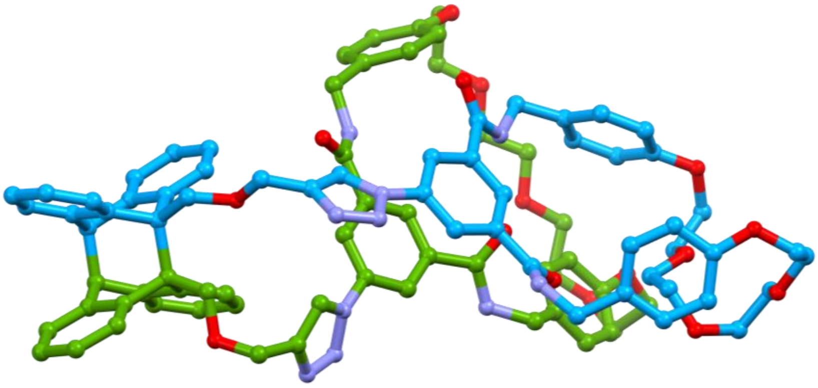

Figure S92 DFT-optimised structure of HH-(**3**$^{\mathbf{An}}$)$_2$. Hydrogen atoms omitted for clarity.

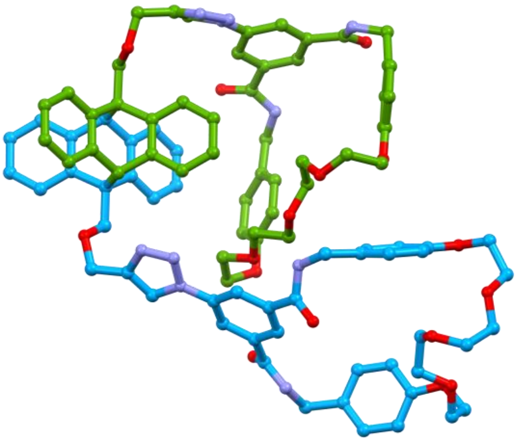

Figure S93 DFT-optimised structure of HT-(**3**$^{\mathbf{An}}$)$_2$. Hydrogen atoms omitted for clarity.

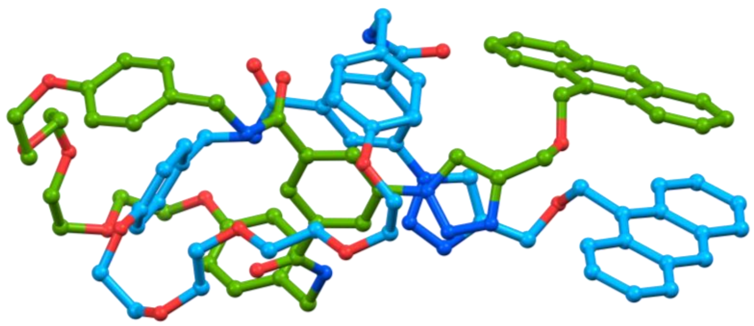

Figure S94 DFT-optimised structure of $\mathbf{4^{An}}$. Hydrogen atoms omitted for clarity.

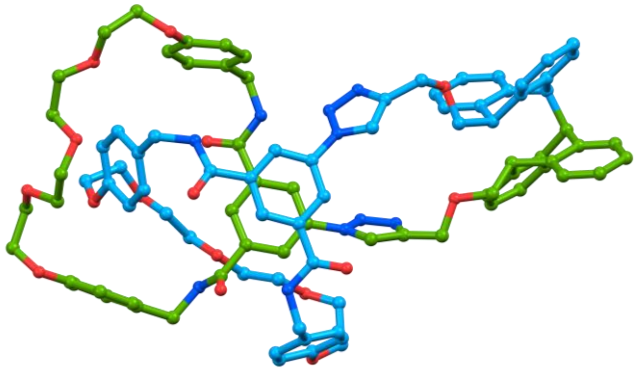

Figure S95 DFT-optimised structure of HH-$\mathbf{4^{An}}$ pretzelane. Hydrogen atoms omitted for clarity. C-C bond lengths across anthracene photodimer bridgehead calculated at 1.620 and 1.678 Å. These values are in good agreement experimental and previously calculated bond lengths for related systems.[18]

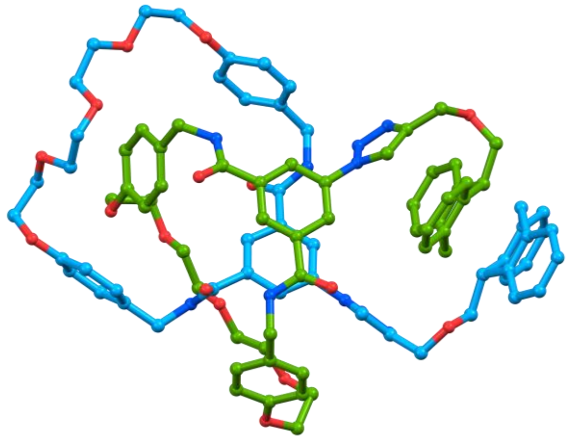

Figure S96 DFT-optimised structure of HT-$\mathbf{4^{An}}$ pretzelane. Hydrogen atoms omitted for clarity. C-C bond lengths across anthracene photodimer bridgehead calculated at 1.636 and 1.640 Å. These values are in good agreement experimental and previously calculated bond lengths for related systems.[18]

Table S5 Calculated relative energies of the HH and HT isomers for the photodimerised adducts of **3**^{An} and **4**^{An}.

Compound	Relative *E* (eV)	Relative *E* (kJ mol^{-1})
HT-(**3**^{An})$_2$	0	0
HH-(**3**^{An})$_2$	0.191	18.4
HT-**4**^{An} pretzelane	0	0
HH-**4**^{An} pretzelane	0.758	73.1

7. References

[1] J. E. M. Lewis, *Chem. Eur. J.*, 2021, **27**, 4454.

[2] N. Ahmad, H. A. Younus,A. H. Chugtai, K. Van Hecke, Z. A. K. Khattak, Z. Gaoke, M. Danish and F. Verpoort, *Catal. Sci. Technol.*, 2018, **8**, 4010.

[3] W.H. Melhuish, *J. Phys. Chem.* 1960, **64**, 762–764.

[4] P.S. Sherin, J. Grilj, Y. P. Tsentalovich and E. Vauthey, *J. Phys. Chem. B*, 2009, **113**, 4953-4962.

[5] B. Dereka and E. Vauthey, *Chem, Sci.*, 2017, **8**, 5057-5066.

[6] J. Maillard, K. Klehs, C. Rumble, E. Vauthey, M. Heilemann and A. Fürstenberg, *Chem. Sci.*, 2021, **12**, 1352-1362.

[7] J.B. Birks, D. J. Dyson, and I. H. Munro, *Proc. Royal. Soc. London. Ser. A Math. Phys. Sci.*, 1963, **275**, 575-588.

[8] P. Conlon, C. J. Yang, Y. Wu, Y. Chen, K. Martinez, Y. Kim, N. Stevens, A. A. Marti, S. Jockusch, N.J. Turro and W. Tan, *J. Am. Chem. Soc.*, 2008, **130**, 336-342.

[9] A. Ruiu, M. Vonlanthen, S.M. Rojas-Montoya, I. González-Méndez and E. Rivera, *Molecules*, 2019, **24**, 4083.

[10] Handbook of Chemist; Nikolsky, B. N., Ed.; State Scientific and Technical Publisher of Chemical Literature: Moscow, 1962; Vol. 1 (in Russian).

[11] K. Stranius and K. Börjesson, *Sci. Rep.*, 2017, **7**, 41145.

[12] A. D. Becke, *J. Chem. Phys*., 1993, 98, 5648-5652.

[13] C. Lee, W. Yang and R.G. Parr, *Phys. Rev. B*, 1988, **37**, 785-789.

[14] S. H. Vosko, L. Wilk and M. Nusair, *Can. J. Phys.*, 1980, **58**, 1200-1211.

[15] P. J. Stephens, F. J. Devlin, C. F. Chabalowski and M. J. Frisch, *J. Phys. Chem.*, 1994, **98**, 11623-11627.

[16] Gaussian 16, Revision C.01, M. J. Frisch, G. W. Trucks, H. B. Schlegel, G. E. Scuseria, M. A. Robb, J. R. Cheeseman, G. Scalmani, V. Barone, G. A. Petersson, H. Nakatsuji, X. Li, M. Caricato, A. V. Marenich, J. Bloino, B. G. Janesko, R. Gomperts, B. Mennucci, H. P. Hratchian, J. V. Ortiz, A. F. Izmaylov, J. L. Sonnenberg, D. Williams-Young, F. Ding, F. Lipparini, F. Egidi, J. Goings, B. Peng, A. Petrone, T. Henderson, D. Ranasinghe, V. G. Zakrzewski, J. Gao, N. Rega, G. Zheng, W. Liang, M. Hada, M. Ehara, K. Toyota, R. Fukuda, J. Hasegawa, M. Ishida, T. Nakajima, Y. Honda, O. Kitao, H. Nakai, T. Vreven, K. Throssell, J. A. Montgomery, Jr., J. E. Peralta, F. Ogliaro, M. J. Bearpark, J. J. Heyd, E. N. Brothers, K. N. Kudin, V. N. Staroverov, T. A. Keith, R. Kobayashi, J. Normand, K. Raghavachari, A. P. Rendell, J. C. Burant, S. S. Iyengar, J. Tomasi, M. Cossi, J. M. Millam, M. Klene, C. Adamo, R. Cammi, J. W. Ochterski, R. L. Martin, K. Morokuma, O. Farkas, J. B. Foresman, and D. J. Fox, Gaussian, Inc., Wallingford CT, 2016.

[17] S. Grimme, J. Antony, S. Ehrlich and H. Krieg, *J. Chem. Phys.*, 2010, **132**, 154104.

[18] C. H. Choi and K. Kertesz, *Chem. Commun.*, 1997, 2199-2200.
